# eCyanation Using 5-Aminotetrazole As a Safer Electrophilic and Nucleophilic Cyanide Source

**DOI:** 10.1021/jacsau.4c00768

**Published:** 2024-10-30

**Authors:** Valerio Morlacci, Marco Milia, Jérémy Saiter, Irene Preet Bhela, Matthew C. Leech, Kevin Lam

**Affiliations:** † School of Science, Faculty of Engineering and Science, University of Greenwich, Chatham Maritime, Chatham, Kent ME4 4TB, United Kingdom

**Keywords:** cyanation reaction, electrochemical cyanation, anodic oxidation, electrosynthesis, flow chemistry, cyanogen bromide, cyanide source

## Abstract

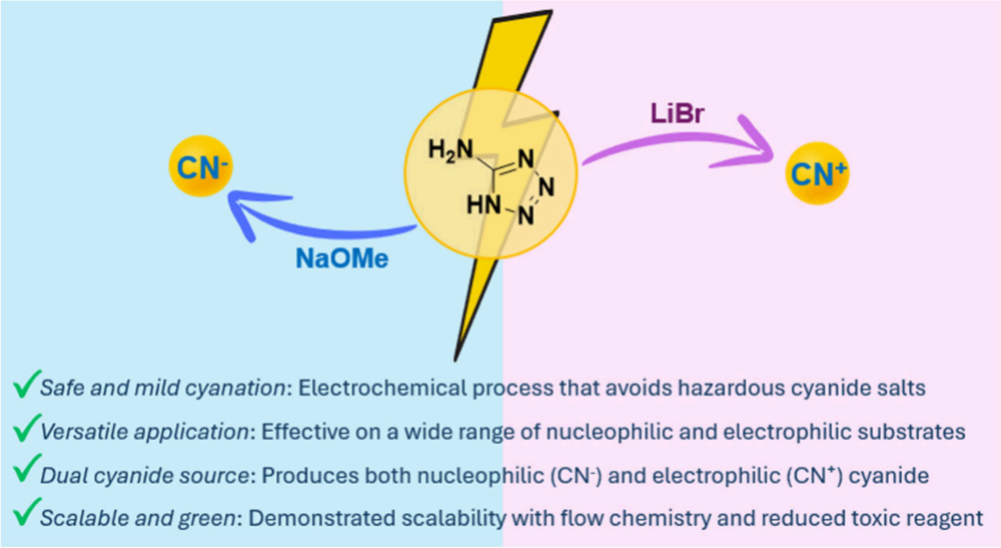

An electrochemical method for carrying out safer cyanation reactions is reported. The use of 5-aminotetrazole as a cyanide source enabled the successful electrogeneration of both electrophilic and nucleophilic cyanide sources. To demonstrate the versatility of the method, a variety of cyanation reactions were carried out, including the synthesis of cyanamides, *N*-heterocycles, and aromatic nitriles, as well as the nucleophilic addition of cyanides to a variety of electrophiles without the need to handle highly toxic cyanide salts. Finally, as a proof of concept for scalability, the cyanation methodology was rapidly transferred to a flow electrosynthesis setup, which demonstrated its potential for large-scale applications.

Cyanation reactions are staple reactions of organic synthesis that exploit the remarkable versatility of the cyano group to forge diverse functional groups, such as amines, amides, carboxylic acids, aldehydes and various N-containing heterocycles.^1^ Indeed, nitriles represent an important class of organic compounds commonly found in pharmaceuticals,^2^ dyes,^3^ and natural products.^4^ However, their preparation often requires harsh conditions and the use of hazardous reagents, such as highly toxic and volatile HCN and TMSCN, which are commonly used in nucleophilic cyanation reactions, or metallic cyanides, such as CuCN, which are used in the Rosenmund–von Braun^5^ and Sandmeyer^6^ reactions to produce aromatic nitriles. When it comes to electrophilic cyanation reactions, BrCN is the most commonly used source of “CN^+^”, which facilitates reactions with N,^7^ O,^8^ S,^9^ and C^10^-centered nucleophiles. However, its use has declined because of its acute toxicity, even in small quantities, and its propensity to be readily absorbed by inhalation or skin contact.^11^ In addition, BrCN requires storage at temperatures between 2 and 8 °C to prevent sublimation, making it impractical and highly hazardous to transport, and it is unsurprisingly on the list of highly restricted substances in some countries.^12^

Over the past decade, considerable efforts have been made to reduce the risks associated with cyanation reactions and, in particular, the handling of toxic cyanide salts where most contamination and accidents are likely to occur. This has led to the development of various alternative nucleophilic and electrophilic sources of cyanide.^13^ Commonly used cyanide surrogates include ferricyanides,^14^ cyanocarbonyls,^15^ and cyanohydrins.^16^ However, these are often synthesized using one of the hazardous cyanide sources previously mentioned. Similarly, sources of electrophilic CN^+^ include N–CN cyanating agents,^17^ cyanates,^18^ cyanosulfonamides,^19^ hypervalent iodine reagents,^20^ and more recently, sulfur-based reagents,^21^ which are also very often prepared using toxic cyanide salts. In addition to the introduction of safer cyanide reagents, novel electrochemical,^22^ photocatalytic,^23^ and flow chemical^24^ processes have emerged to mitigate the harsh conditions typically associated with cyanation reactions.

Electrosynthesis provides a reliable alternative to harsh thermal reactions by offering milder reaction conditions and simple procedures. Consequently, numerous electrochemical studies have been carried out to synthesize both aromatic and aliphatic nitrile derivatives, often using TMSCN or NaCN as the cyanide source.^25^

However, although these methods are more environmentally friendly and safer, they still require the handling and storage of toxic and flammable reagents, which is costly and not without risk.

Therefore, a novel and practical electrochemical method for both the generation of nucleophilic and electrophilic cyanide sources and their subsequent use in cyanation reactions is disclosed in this article. The need to handle toxic and hazardous reagents is avoided, thus minimizing the risks classically associated with such transformations. By anodic oxidation of aminotetrazole and variation of the supporting electrolyte/base system, either an electrophilic or nucleophilic cyanide source, such as BrCN or CN^–^, can be successfully generated in situ (Figure 1). This electrochemical cyanation represents a practical and safer alternative to classical cyanation methods, especially as cyanides are electrogenerated in a controlled manner “on demand.” Based on reported studies of thermal decomposition^26^ and our previous work on anodic oxidation of aminotetrazole derivatives,^27^ our investigation began by exploring the possibility of electrochemically generating an electrophilic cyanide. Indeed, the anodic oxidation of aminotetrazole should result in the formation of cyanide anions, which, in the presence of electrogenerated “Br^+^″ species or Br_2_, should lead to the formation of the desired BrCN. We started our studies using an undivided electrochemical cell and LiBr as the bromide source. To our delight, when the anodic oxidation was carried out in a mixture of acetonitrile and water, the formation of the desired BrCN was identified by ^13^C NMR (see the Supporting Information). Based on this encouraging result, we decided to investigate the possibility of using the electrogenerated BrCN for the production of cyanamides.^17c^

**Figure 1:**
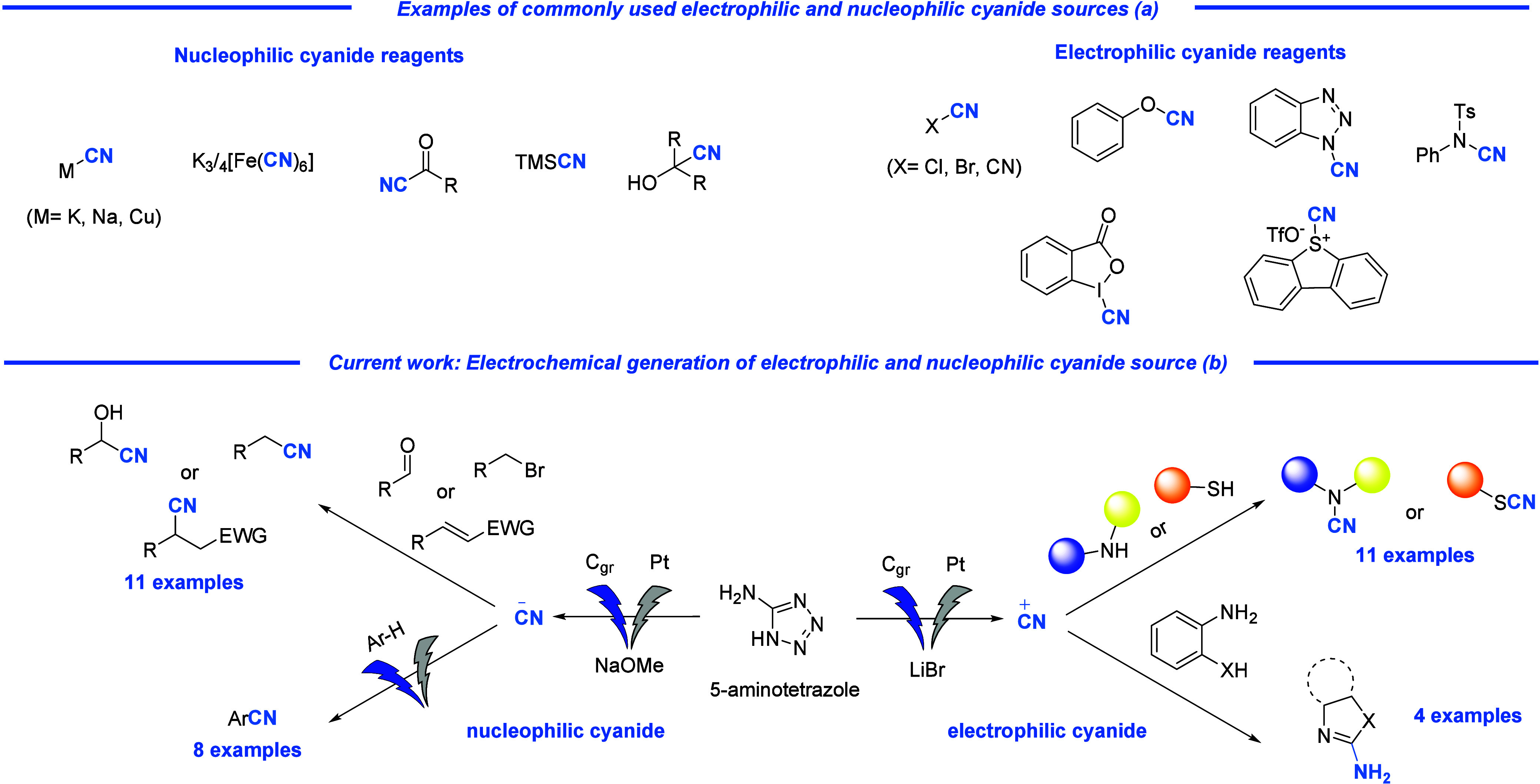
Electrical generation of cyanide sources.

Optimization studies (detailed in the Supporting Information) have shown that cyanogen bromide can be efficiently produced in situ by electrolysis of a solution of 5-aminotetrazole at *J* = 41.7 mA·cm^–2^ for 2.8 *F* in a mixture of CH_3_CN/H_2_O in a 25:1 ratio using a graphite anode and a low hydrogen overvoltage cathode, such as platinum (Table 1).

**Table 1:**
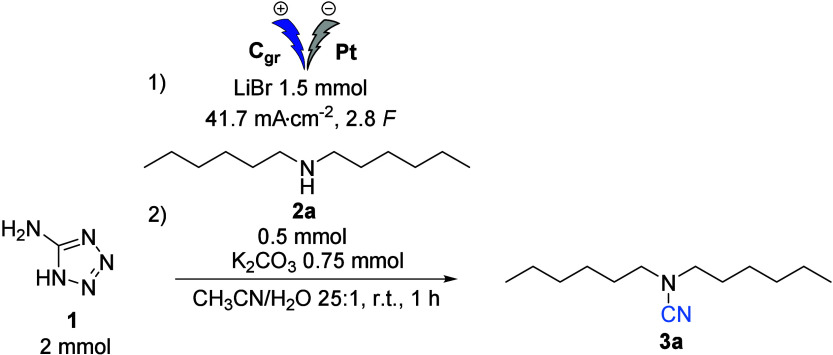
Optimization of Conditions for Cyanation of Amines

entry	deviation from standard conditions	yield %^a^
1	none	86
2	MeOH instead of CH_3_CN/H_2_O 25:1	22
3	no base	75
4	1 mmol of LiBr instead of 1.5 mmol	68
5	2 *F* instead of 2.8 *F*	64

a Isolated yield.

The amine was then added to the cell postelectrolysis and led to the formation of the desired cyanamide with a yield of 75% without base (Entry 3, Table 1). Furthermore, the addition of 1.5 equiv of potassium carbonate as a base in the second step increased the yield to 86% (Entry 1, Table 1) after 1 hour of stirring at room temperature.

With the optimum reaction conditions in hand, the scope of the novel cyanation methodology was investigated (Table 2).

**Table 2:**
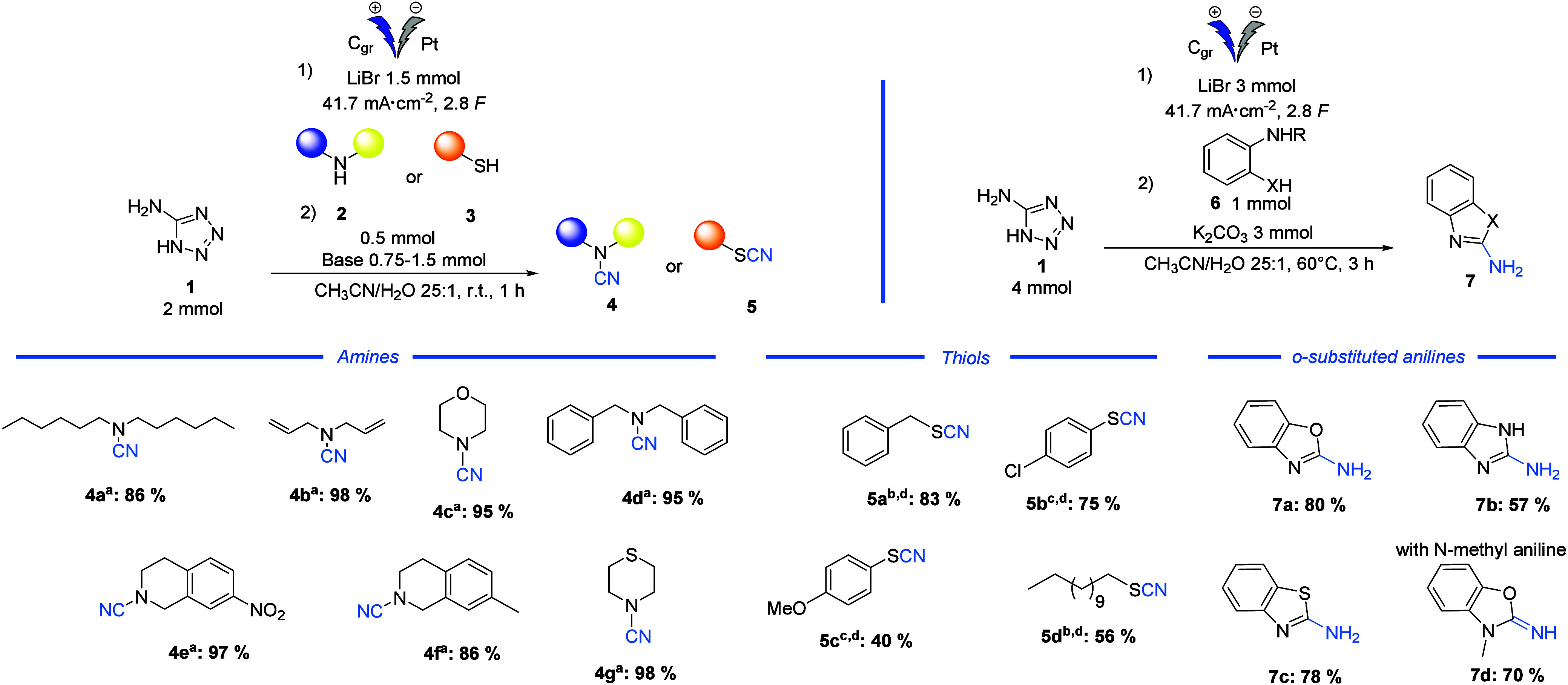
Substrate Scope with an Electrophilic Cyanide Source

a K_2_CO_3_, 0.75 mmol.

b Et_3_N, 1.5 mmol.

c DBU, 1.5 mmol.

d Thiol added at 0 °C.

The reaction showed good to excellent yields over a range of amines (**4a**–**g**) with the desired products obtained by a simple basic aqueous workup, thereby bypassing any need for chromatographic purification.

To further extend the method, the reaction was carried out with thiols as nucleophiles using the same procedure previously optimized for amines with changing of the concentration. In this case, however, Et_3_N and DBU were used as bases as they are known to limit the formation of the disulfide byproduct. Et_3_N proved to be the ideal base for alkyl thiols, which yielded 56% and 83% of the desired thiocyanates **5d** and **5a** using dodecylthiol and benzylthiol, respectively, while DBU was shown to give better yields of the desired cyanated thiols from aromatic thiols, such as *p*-methoxybenzenethiol **3c** and *p*-chlorobenzenethiol **3b**. Finally, we also tested the reaction with ortho-substituted anilines bearing different nucleophilic groups (−OH, −SH, −NH_2_) with the aim of synthesizing heterocyclic compounds. As shown in Table 1, by stirring the solution for 3 h at 60 °C, we successfully obtained the desired heterocycle **7b** in moderate yield starting from phenyl-1,2-diamine. In addition, the use of thiol **7c** and phenol **7a** led to the formation of their corresponding heterocycles in good yields of 78% and 80%, respectively. In particular, the use of 2-(methylamino)phenol was highly effective and led to product **7d** in a yield of 70%.

To further explore the potential of deriving a cyanide source from 5-aminotetrazole, and inspired by the promising results from the electrogeneration of BrCN, we focused the second part of our investigations on the feasibility of generating a nucleophilic cyanide source. We decided to test the anodic synthesis of CN^–^ and use it directly in a subsequent electrochemical aromatic cyanation.^28^ The conditions have been optimized using dimethoxybenzene as a model substrate (full optimization details can be found in the Supporting Information). First, nucleophilic cyanides are prepared by electrolyzing 3 equiv of 5-aminotetrazole with 3 equiv of sodium methoxide in methanol. This reaction is carried out at a current density of *J* = 41.7 mA·cm^–2^ for 2 *F/mol* of tetrazole using a graphite anode and a platinum cathode. For the aromatic cyanation step, the graphite anode is then replaced by a platinum one, 1 equiv of arene is added, and the electrolysis is continued at *J* = 2.8 mA·cm^–2^ for 2.3 *F* of arene, which results in an isolated yield of 83% of the desired aromatic nitrile (Entry 1, Table 3). While it is still possible to keep using graphite as the anode during the electrochemical aromatic cyanation step, it results in a lower nitrile yield of 53% (Entry 2, Table 3). Increasing the current density in the second step to 13.9 mA/cm^2^ also reduces the yield to 71% (Entry 3, Table 3). In addition, this increase in the current intensity reduces the selectivity of the reaction and leads to the formation of numerous byproducts, including the dimer of the arene, as well as its dicyanated product.

**Table 3:**
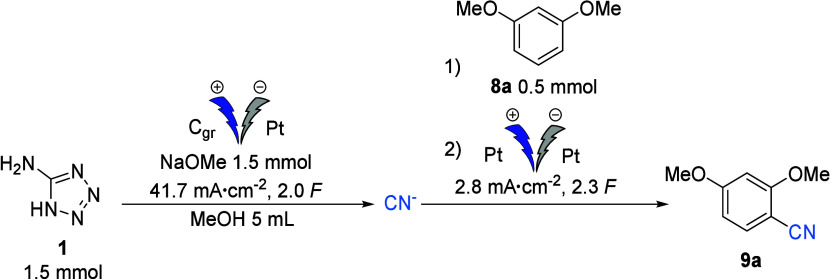
Conditions Optimization of Cyanation of Electron-Rich Aromatic Rings

entry	deviation from standard conditions	yield %^a^
**1**	none	83
2	C_gr_ as anode in the 2° electrolysis	53
3	13.9 mA/cm^2^ instead of 2.8 mA/cm^2^ during the 2° electrolysis	71
4	C_gr_ as cathode and Pt as anode in the 2° electrolysis	55
5	0.5 equiv of NaOMe instead of 3 equiv	10
6	NaOH instead of NaOMe	

a Isolated yield.

Having identified the optimal conditions, the scope and limitations of the one-pot cyanide generation/aromatic cyanation sequence were explored (Table 4).

**Table 4:**
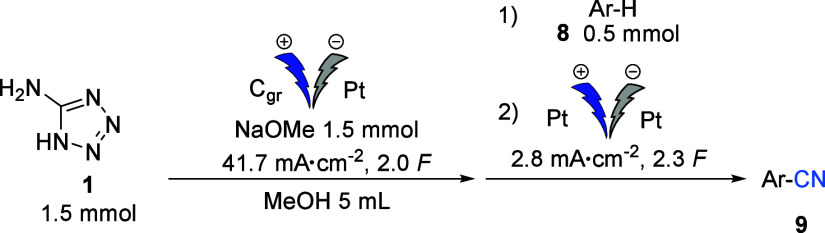

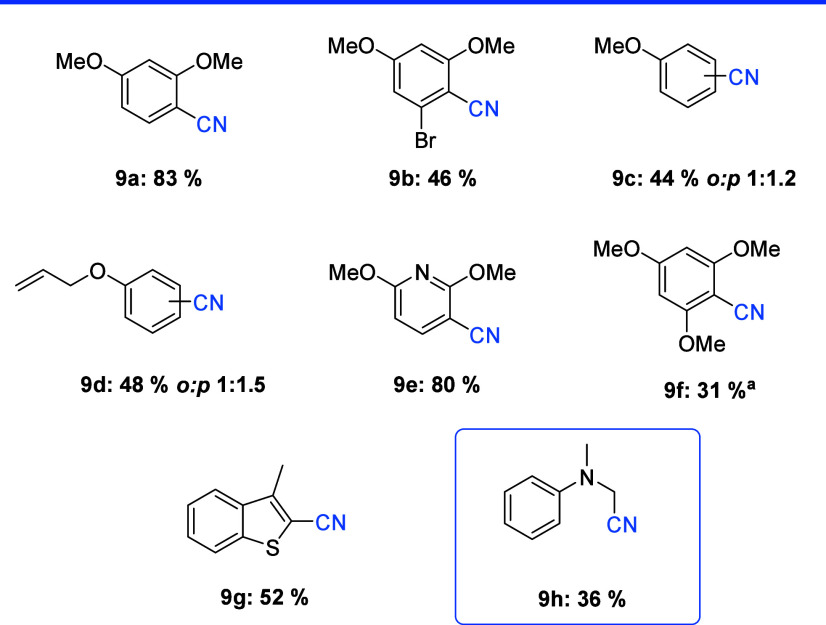
Electrochemical Cyanation of Electron-Rich Aromatic Rings

a Reaction conducted using 13.9 mA·cm^–2^

The electrochemical cyanation reaction was carried out using various substituted aromatic compounds, including 2,5-dimethoxypyridine **8e** and 3-methylbenzothiophene **8g**, which led to the formation of the desired nitriles in moderate to good yields. Using 1,3,5-trimethoxybenzene as the starting material, the corresponding product **9f** was only obtained by increasing the current density to *J* = 13.9 mA·cm^–2^ with a yield of 31%, the main byproduct being the dimer. In addition, under the optimized conditions, the reaction with *N,N*-dimethylaniline **8h** proceeded predominantly via a Shono-type oxidation of the methyl group^29^ to give a yield of 36% of the corresponding aliphatic nitrile product **9h**.

To further demonstrate the versatility of this electrochemical cyanation reaction, we extended its application to nucleophilic cyanation reactions using aldehydes as electrophilic acceptors. Initially, we used the conditions previously developed for the first electrolysis of the aromatic cyanation but increased the amount of the reactant to 1 mmol. After electrolysis, the aldehyde was added, and the solution was stirred for 1 hour at room temperature to give the desired cyanohydrins **11a**–**f** with yields ranging from 68 to 84% (Table 5).

**Table 5:**
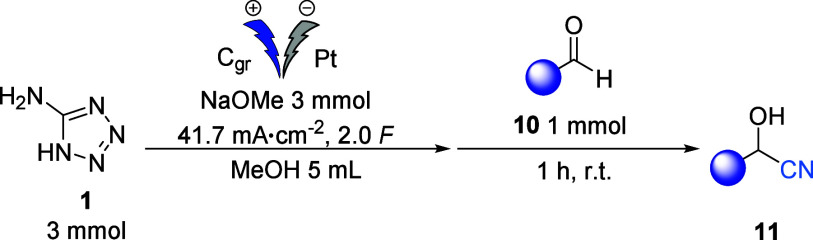

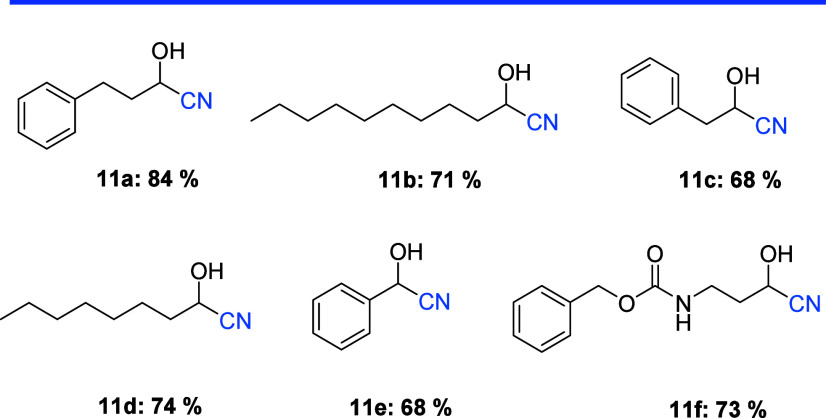
Cyanation Reaction of Aldehydes

Finally, the nucleophilic addition of cyanides to various electrophiles was investigated. These included the synthesis of α-aminonitrile from imine, nucleophilic substitution on benzylic bromides, and Michael addition reactions on chalcone and nitrostyrene derivatives. As shown in Scheme 1, the reactions proceeded successfully with different electrophiles. When benzyl bromide was used, nucleophilic substitution with cyanide carried out at 60 °C for 5 hours gave a 57% yield of the corresponding nitrile. In contrast, the use of the *p*-brominated derivative resulted in a 71% yield of product **13b**.

**Scheme 1:**
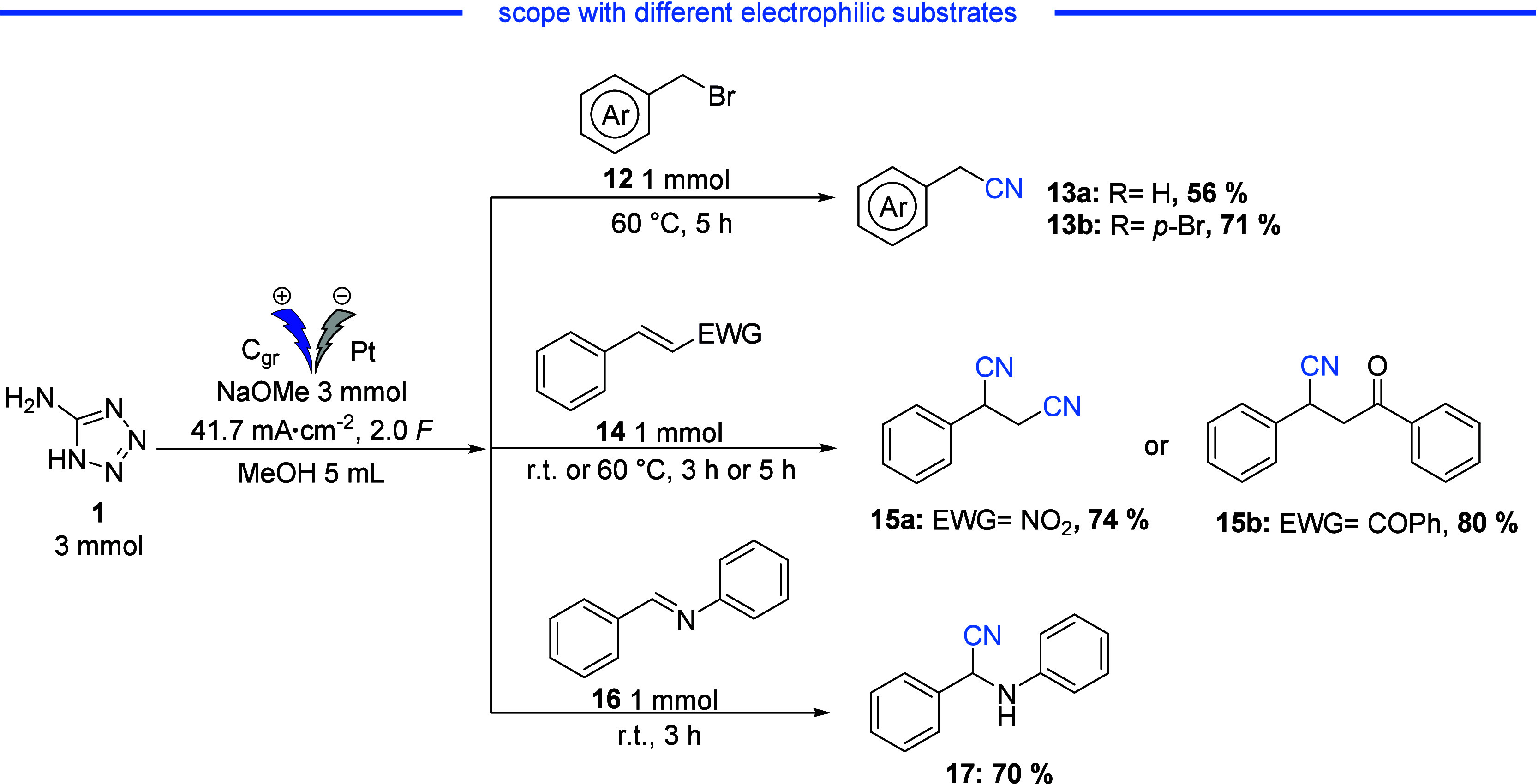
Cyanation Reaction on Different Electrophilic Substrates

Using a Michael acceptor, such as chalcone, as the electrophilic acceptor led to the formation of **15b** with an excellent yield of 80%. Similarly, when starting with nitrostyrene, the cyanation reaction led to the formation of 2-phenylsuccinonitrile **15a** in a yield of 74%.^30^ The reaction was also carried out using *(E)*-*N*,1-diphenylmethanimine **16** as the electrophile and led to the corresponding nitrile **17** in a yield of 70%.

To further demonstrate the scalability and practicality of our cyanide generation from 5-aminotetrazole, we investigated whether our method could be transferred to flow electrochemistry. Indeed, flow chemistry has been shown to be remarkably effective in rapidly scaling up electrochemical reactions^31^ and is particularly ideal when two reactions need to be performed back-to-back, as in our case. In addition, the use of this state-of-the-art method further enhances the practicality and safety of our method as it allows the entire process to take place in a closed system, which avoids any potential exposure to cyanide during the reaction. We first investigated whether the electrolysis of a mixture of lithium bromide and aminotetrazole could be carried out in a flow system. A 0.045 M solution of lithium bromide and 0.06 M aminotetrazole was electrolyzed through an electrochemistry flow cell at a rate of 0.19 mL·min^–1^ and using a current density of 5.5 mA·cm^–2^ with a carbon graphite (C_gr_) anode and a stainless steel cathode. A 5.5 mL aliqout of this electrolyzed solution was added to 2.5 mL of a 0.033 M solution of dibenzylamine and 0.05 M potassium carbonate.

After being stirred for 1 hour at room temperature, complete conversion of the substrate was observed with only the desired cyanamide being formed. The two steps were then coupled in a flow. The solubility of potassium carbonate proved to be a limitation in the development of this process, and eventually a 10:1 acetonitrile/water mixture had to be used to contain both the amine and potassium carbonate (Scheme 2).

**Scheme 2:**
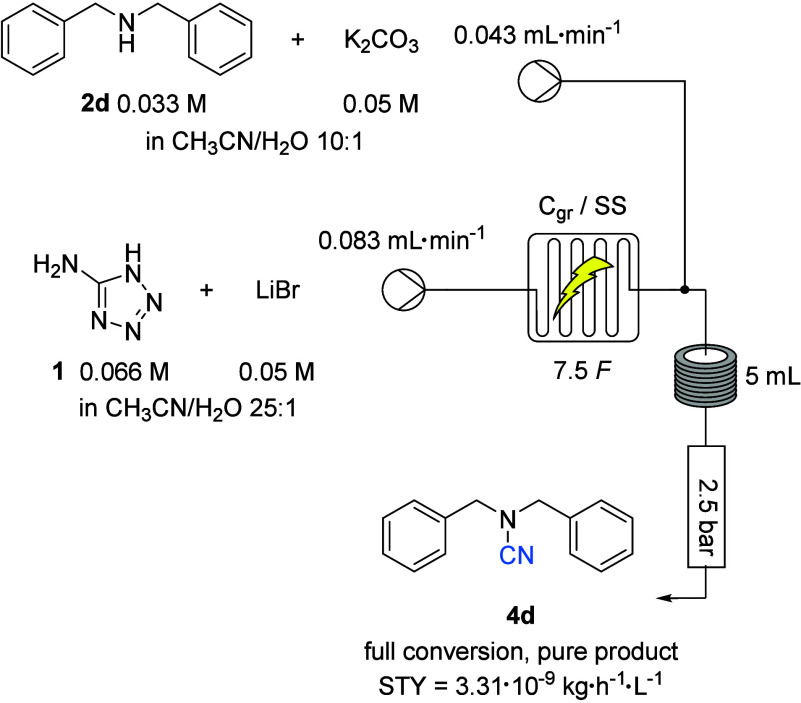
Continuous Flow eCyanation of Amine

The use of a 5 mL reactor and a T-shaped mixer, together with a reduced flow rate of 0.083 mL·min^–1^ for the electrolyzed solution, resulted in a quantitative yield of product after collection for 1 hour and 47 minutes, which corresponded to a space–time yield of 3.31 × 10^–9^ kg·h^–1^·L^–1^ compared to 4.22 × 10^–8^ kg·h^–1^·L^–1^ in batch. Nevertheless, the scale-up of batch electrosynthesis can be challenging and ultimately limited. Therefore, flow methods remain of interest for scale-up purposes.^32^ In addition, our flow method allows the entire conversion to be performed in a closed system, thereby limiting potential risks, especially on larger scales. A possible mechanism for the electrochemical generation of cyanide from 5-aminotetrazole is shown in Scheme 3. After tetrazole deprotonation, the anion is anodically oxidized to form an unstable fulvene, which subsequently loses two nitrogen molecules to form a cyanide anion.^27^

**Scheme 3:**
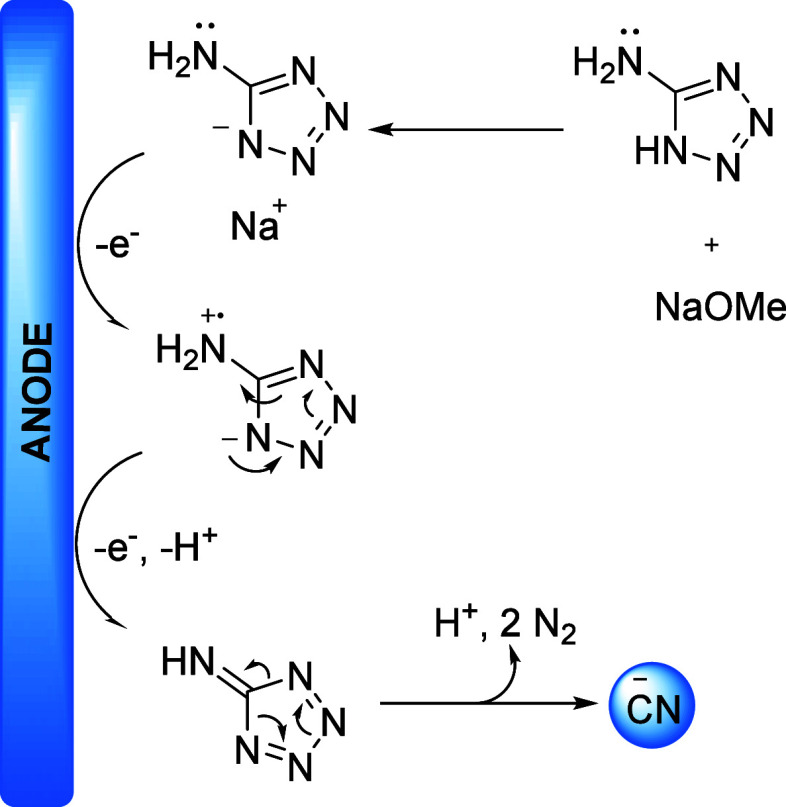
Proposed Mechanism for the Formation of CN^–^

In conclusion, we have successfully developed a mild, practical, and safe electrogeneration of nucleophilic and electrophilic cyanide sources starting from 5-aminotetrazole. The method was found to be effective in various examples of cyanation reactions with good yields. Finally, we have demonstrated the applicability of this procedure by carrying out the reactions using flow electrochemistry with excellent results.

## Supporting Information Available

Experimental procedures, characterization data, NMR spectra (PDF)
