# Supplementary material for: eCyanation Using 5-Aminotetrazole As a Safer Electrophilic and Nucleophilic Cyanide Source
Source: JACS Au. 2024 Oct 30;4(11):4199–205. doi: 10.1021/jacsau.4c00768 (PMC11600158; doi:10.1021/jacsau.4c00768)
Supplement: Supplementary file 1 — au4c00768_si_001.pdf [file au4c00768_si_001.pdf]

eCyanation using 5-aminotetrazole as a safer electrophilic and nucleophilic cyanide source.

Valerio Morlacci,[a] Marco Milia,[‡a] Dr. Jérémy Saiter,[‡a] Irene Preet Bhela,[a] Dr. Matthew C. Leech[a] and Prof. Dr Kevin Lam*[a]

[a] School of Science, Faculty of Engineering and Science, University of Greenwich, Chatham Maritime, Chatham, Kent, ME4 4TB, U.K.

*k.lam@greenwich.ac.uk

‡*M.M. and J.S. contributed equally to this paper*

Abstract: An electrochemical method for carrying out safer cyanation reactions is reported. The use of 5-aminotetrazole as a cyanide source enabled the successful electrogeneration of both electrophilic and nucleophilic cyanide sources. To demonstrate the versatility of the method, a variety of cyanation reactions were carried out, including the synthesis of cyanamides, N-heterocycles, aromatic nitriles, as well as the nucleophilic addition of cyanides to a variety of electrophiles, without the need to handle highly toxic cyanide salts. Finally, as a proof of concept for scalability, the cyanation methodology was rapidly transferred to a flow electrosynthesis setup, demonstrating its potential for larger scale applications.

Contents

Material and methods 2

General Experimental Procedures 2

Batch and Flow Electrochemical Reaction Setup 3

Optimization studies for electro-generation of cyanogen bromide 4

Optimization studies for cyanation reaction on aromatic rings 5

^{13}C NMR Spectra of BrCN 6

Continuous flow eCyanation of amines 7

General procedure for the synthesis of cyanamide derivatives 8

General procedure for the synthesis of thiocyanate derivatives 11

General procedure for the synthesis of heterocycles derivatives 12

General procedure for the cyanation of elettron-rich aromatic rings 14

General procedure for the cyanation reactions on aldehydes 18

General procedure for the cyanation reactions on electrophilic compounds 20

References 24

NMR Spectra 25

Material and methods

General Experimental Procedures

All reactions were carried out under aerobic conditions unless otherwise stated. All solvents and commercially available reagents were purchased from standard vendors and used without further purification unless otherwise stated. Electrolyses were performed using an IKA Electrasyn 2.0 using carbon graphite (**C$_{gr}$**) working electrode (**WE**) and platinum foil (**Pt**) counter electrode (**CE**) using a variable stirring rate between 400 - 1500 rpm. For the aromatic cyanation reactions the electrolyses were performed using an IKA Electrasyn 2.0 using platinum foil (**Pt**) working electrode (**WE**) and platinum foil (**Pt**) counter electrode (**CE**) using a variable stirring rate between 400 - 600 rpm. Analytical thin-layer chromatography (TLC) was performed using silica gel plates (0.25 mm thickness) on aluminium support. Visualization was accomplished by irradiation with a UV lamp and/or staining with either $KMnO_4$ or ninhydrin. Column chromatography was performed over Silica gel 60 Å (40-63µ mesh) using a CombiFlash Rf Lumen automatic flash chromatography system. Residual solvent was removed using a static oil pump (< 10 mbar). The cooling of reaction mixtures was achieved using an ice bath (0 °C).

NMR spectra were obtained using a JEOL ECZR 400 (^{1}H 399.78 MHz; ^{13}C 100.53 MHz) or ECA 500 (^{1}H 500.16 MHz; ^{13}C 125.77 MHz) spectrometer and are reported relative to the residual solvent resonances. All heteronuclear NMR spectra were ^{1}H decoupled and recorded at room temperature unless otherwise stated. Data for ^{1}H NMR spectra are reported as follows: chemical shift (δ, ppm), coupling constant (Hz), multiplicity (s, singlet; d, doublet; t, triplet; m, multiplet; br, broad) and integration. Data for ^{13}C and ^{19}F NMR are reported in terms of chemical shift (δ, ppm). IR spectra were recorded on a Perkin Elmer Spectrum Two instrument as neat samples.

High Resolution Mass Spectrometry (HRMS) data were obtained by Dr. Iain Goodall and Bini Claringbold of the University of Greenwich Mass Spectrometry Service using a Waters Synapt G2 hybrid Quadrupole-orthogonal acceleration time-of-flight configuration (Waters, Manchester, UK) operating in Resolution Mode (M/ΔM ≥ 18,000), fitted with a Waters Acquity UPLC binary solvent chromatographic pump system. The column used was a reversed-phase Acquity BEH C18 2.1 x 50 mm, 1.7-micron bead, running a 3- minute separation with an A:B eluent mixture comprising of either deionised water with 0.1% (v:v) formic acid and acetonitrile with 0.1% (v:v) formic acid (negative mode) respectively or deionised water with 0.1% (v:v) ammonium hydroxide and acetonitrile with 0.1% (v:v) ammonium hydroxide (positive mode) respectively. Mass calibration of the instrument was performed using sodium formate cluster ions, and an orthogonal Lock-SprayTM ESI probe was used with a lock mass calibrant, leucine-enkephalin. The pseudomolecular leucineenkephalin ion at *m/z* = 554.2615 (Negative Ion Mode), and *m/z* = 556.2771 (Positive Ion Mode), was used as the internal mass correction calibrant. Additional samples were analyzed on a Thermo LTQ Orbitrap XL coupled with a heated electrospray source (HESI). The capillary temperature was set to 275 °C and a voltage of 21 V. The sheath gas and auxiliary gas flow were set to 10 and 5 L h^{-1} respectively and the source current and voltage set to 100 µA and 5 kV. A solution of analyte (0.1 mg/ml) and sodium formate (1% v/v) in acetonitrile was added by direct infusion (10 µL/min) into the mass spectrometer using a Hamilton syringe (250 µL).

Flow chemistry experiments have been realized using an Asia® Electrochemistry Flow Chemistry System including Asia® Syringe Pump, FLUX Electrochemistry reactor with a carbon gasket electrode (WE) and a stainless-steel electrode (CE) (internal volume of the cell = 225 µL) and Pressure Controller. PTFE tubbings of 0.5 mm internal diameter have been used. Yields have been evaluated after reaching steady state.

Batch and Flow Electrochemical Reaction Setup

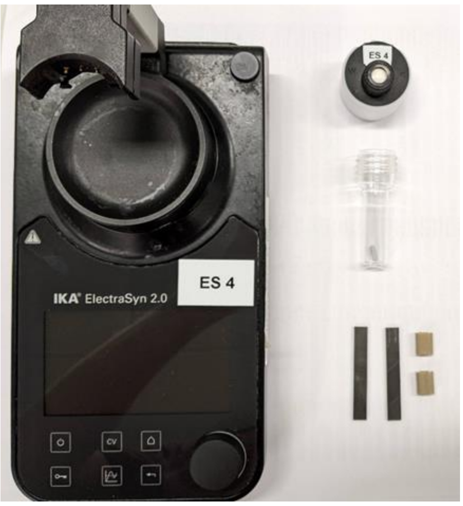

Figure S1

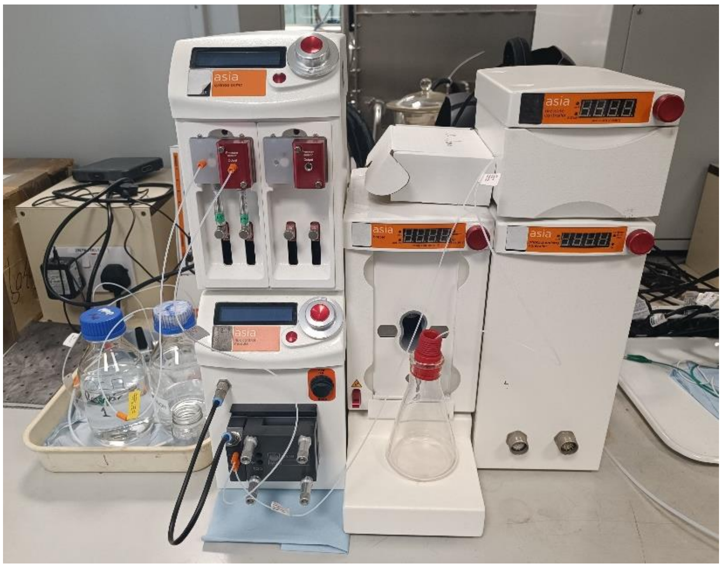

Figure S2

Optimization studies for electro-generation of cyanogen bromide

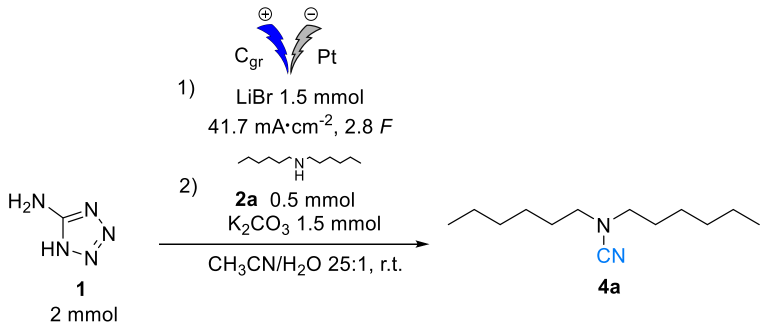

Entry	Deviation from optimised conditions	Yield
**1**	none	86 %
**2**	5 mL instead of 10 mL	76 %
**3**	No base	75 %
**4**	2 *F* instead of 2.8 *F*	64 %
**5**	CH_3CN/H_2O 1:1 instead of CH_3CN/H_2O 25:1	25 %
**6**	MeOH instead of CH_3CN/H_2O 25:1	22 %
**7**	EtOH instead of CH_3CN/H_2O 25:1	-
**8**	$NaHCO_3$ instead of K_2CO_3	82 %
**9**	1 mmol LiBr instead of 1.5 mmol	68 %

Table S1

Optimization studies for cyanation reaction on aromatic rings

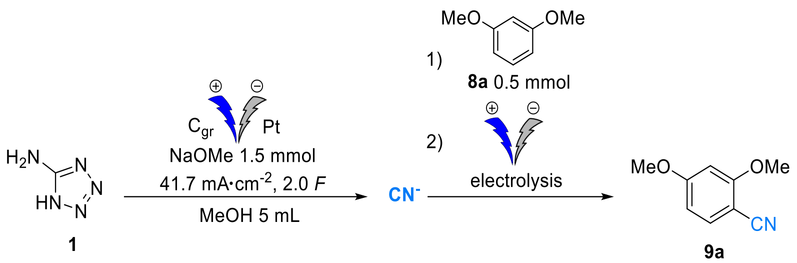

Entry	AT (eq)	NaOMe (eq)	1 electrol.	2 electrol.	**Yield (%)**
1	5	0.5	**w** Cgr/Pt **c** 100 mA, 2 *F*	**w** Cgr/Pt **c** 25 mA, 5.5 *F*	10 %
2	5	0.5[a]	**w** Cgr/Pt **c** 100 mA, 2 *F*	**w** Cgr/Pt **c** 25 mA, 5.5 *F*	<5%
3[b]	5	0.5	**w** Cgr/Pt **c** 100 mA, 2 *F*	**w** Pt/Pt **c** 25 mA, 5.5 *F*	-
4	3	2	**w** Cgr/Pt **c** 100 mA, 2 *F*	**w** Cgr/Pt **c** 100 mA, 2.3 *F*	19 %
5	3	2	**w** Cgr/Pt **c** 100 mA, 2 *F*	**w** Pt/Pt **c** 25 mA, 2.3 *F*	63 %
6	3	2	**w** Cgr/Pt **c** 100 mA, 2 *F*	**w** Cgr/Pt **c** 25 mA, 5.5 *F*	53 %
7[c]	3	3	**w** Cgr/Pt **c** 100 mA, 2 *F*	**w** Pt/Pt **c** 25 mA, 2.3 *F*	71 %
8	3	3	**w** Cgr/Pt **c** 100 mA, 2 *F*	**w** Pt/Pt **c** 5 mA, 2.3 *F*	**83 %**
9	3	3	**w** Cgr/Pt **c** 100 mA, 2 *F*	**w** Pt/Cgr **c** 25 mA, 5.5 *F*	55 %
10	3	3[d]	**w** Cgr/Pt **c** 100 mA, 2 *F*	**w** Pt/Pt **c** 25 mA, 2.3 *F*	-

Table S2

[a] NaOMe in solution of MeOH 5.4M; [b] Dimethoxybenzene present during the first electrolysis; [c] 1 mmol instead 0.5 mmol; [d] NaOH instead NaOMe

^{13}C NMR Spectra of BrCN

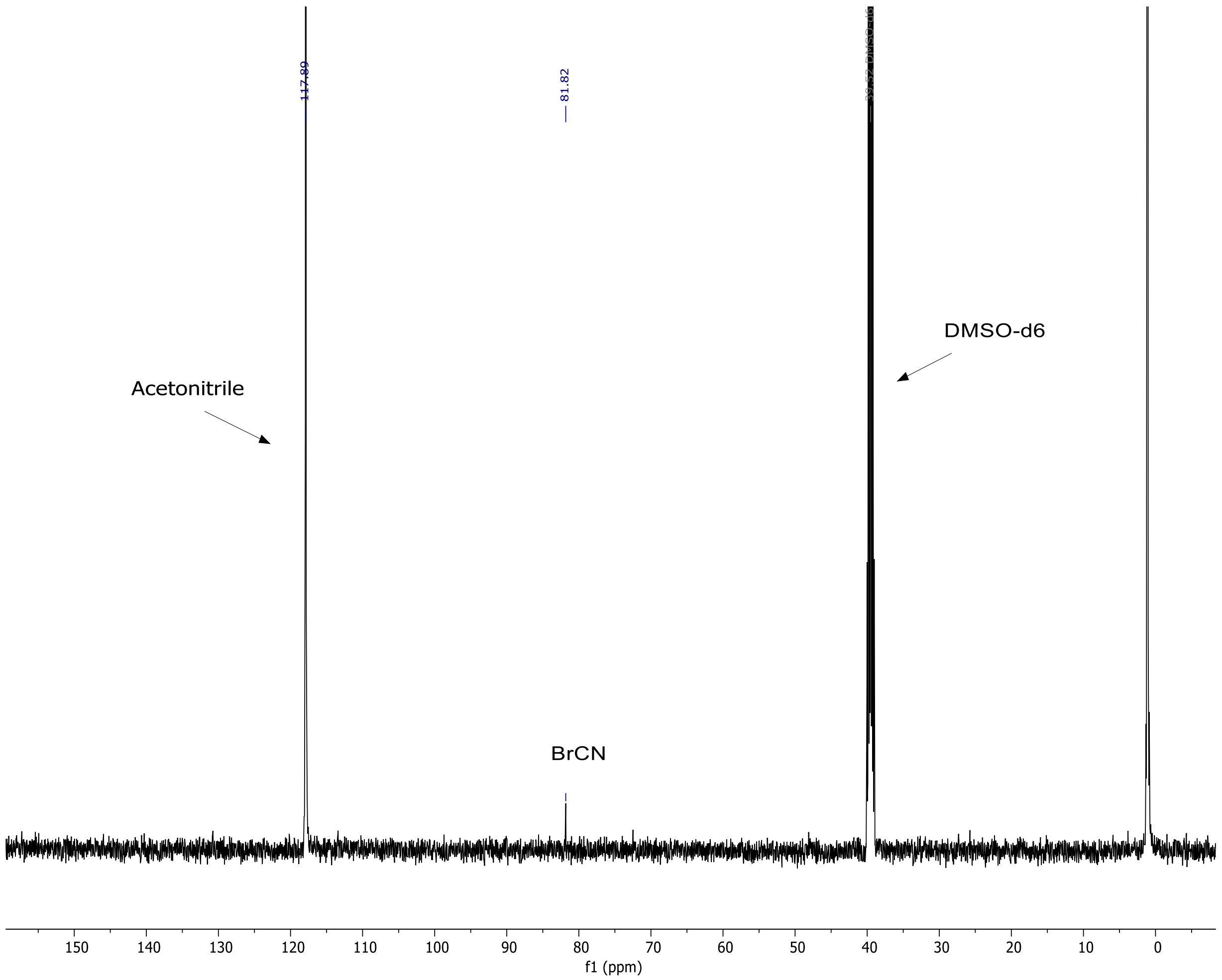

Figure S3: ^{13}C NMR spectra of BrCN, after the electrolysis, done in DMSO-d_6 and compared with the spectra reported in literature.[1]

Continuous flow eCyanation of amines

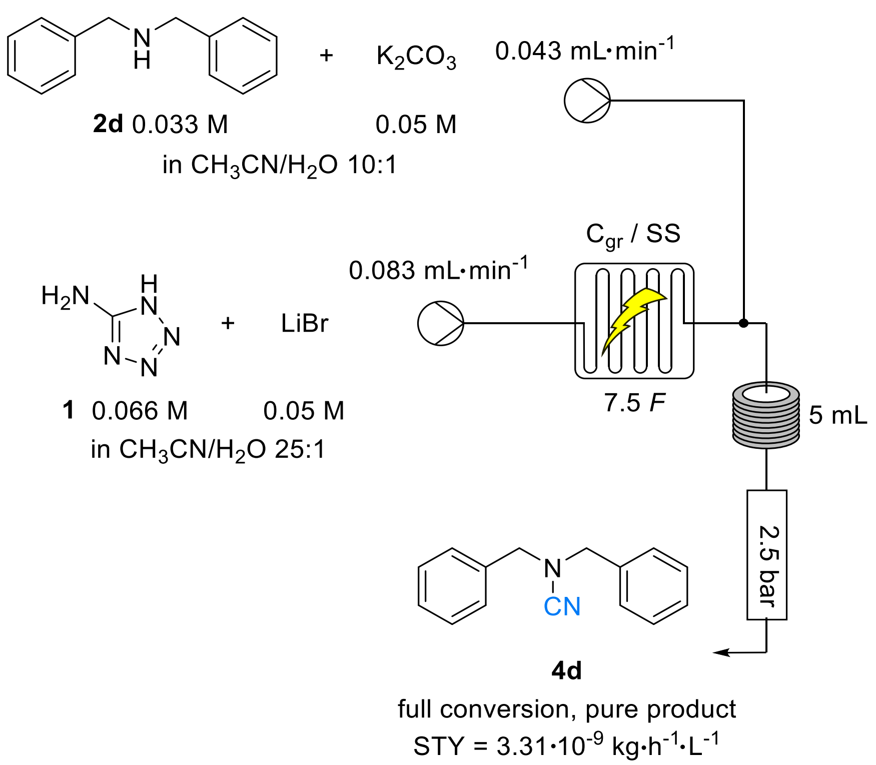

Scheme S1

A solution LiBr (3 equiv., 0.05 M) and 5-aminotetrazole (4 equiv., 0.06 M) in a 25:1 mixture of CH_3CN/H_2O was pumped at 0.083 mL.min^{-1} through an Asia® FLUX reactor (225 µL, 50 mA) followed by a T-shaped mixer. A solution of substrate (1 equiv., 0.033 M) and K_2CO_3 (1.5 equiv., 0.05 M) in 10:1 mixture of CH_3CN/H_2O was pumped through the T-shaped mixer (180° from the first solution inlet). To the outlet of the mixer is plugged a 5 mL reactor followed by an Asia® Pressure Controller set at 2.5 bar. After the steady state was reached, the reaction mixture was collected for 1h 47 and then quenched with a saturated aqueous solution of $NaHCO_3$, extracted with AcOEt and dried with Na_2SO_4. The solvent is removed under reduced pressure to afford the desired product without further purification (0.15 mmol, 33 mg, 99 %).

General procedure for the synthesis of cyanamide derivatives

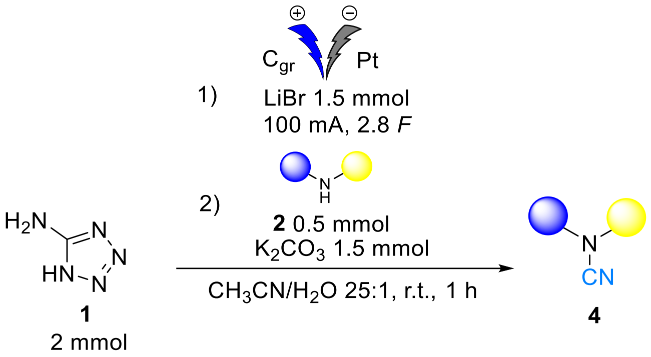

General procedure A: 2 mmol of 5-aminotetrazole (170 mg), 1.5 mmol of LiBr (130 mg) and 5 mL of CH_3CN/H_2O 25:1 are added to a 5 mL one compartment Electrasyn vial. The solution is then electrolyzed with a 41.7 mA.cm^{-2} current density for 2.8 *F* using a carbon graphite anode (WE) and a platinum cathode (CE). At the end of the electrolysis, 0.5 mmol of amine and 0.75 mmol of K_2CO_3 (104 mg) are added and the solution is stirred for 1h at r.t. The reaction mixture is then quenched with a saturated aqueous solution of $NaHCO_3$, extracted with AcOEt and dried with Na_2SO_4. The solvent is removed under reduced pressure to afford the desired product without further purification.

N,N-dihexylcyanamide

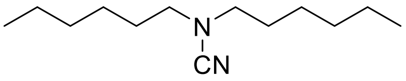

4a

Prepared according to the general procedure **A** using dihexylamine (93 mg, 0.5 mmol). The crude mixture was extracted to afford product **4a** as colorless oil (90 mg, 0.43 mmol, 86 %).

^{1}H NMR (400 MHz,$CDCl_3$): δ= 3.00 – 2.92 (m, 4H), 1.69 – 1.57 (m, 4H), 1.32 (m, 12H), 0.96 – 0.84 (m, 6H).

^{13}C NMR (101 MHz, $CDCl_3$): δ= 118.1, 51.6, 31.5, 27.7, 26.2, 22.6, 14.1.

HRMS (ESI) m/z: $[M+H]^+$ calcd for $C_{13}H_{27}N_2$ 211.2174, found 211.2175

Spectral data are in good agreement with previously published data.[2]

N,N-diallylcyanamide

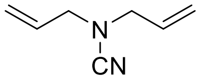

4b

Prepared according to the general procedure **A** using diallylamine (49 mg, 0.5 mmol). The crude mixture was extracted to afford product **4b** as colorless oil (60 mg, 0.49 mmol, 98 %).

^{1}H NMR (500 MHz, $CDCl_3$): δ= 5.90 – 5.78 (m, 2H), 5.39 – 5.28 (m, 4H), 3.62 (m, 4H).

^{13}C NMR (126 MHz, $CDCl_3$): δ= 131.1, 120.7, 117.7, 53.5.

HRMS (ESI) m/z: $[M+H]^+$ calcd for $C_7H_{11}N_2$ 123.0922, found 123.0931

Spectral data are in good agreement with previously published data.[3]

Morpholine-4-carbonitrile

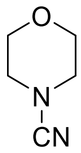

4c

Prepared according to the general procedure **A** using morpholine (44 mg, 0.5 mmol). The crude mixture was extracted to afford product **4c** as colorless oil (53 mg, 0.47 mmol, 95 %).

^{1}H NMR (400 MHz,$CDCl_3$): δ= 3.77 – 3.70 (m, 4H), 3.27 – 3.20 (m, 4H).

^{13}C NMR (101 MHz,$CDCl_3$): δ= 117.4, 65.8, 49.0.

HRMS (ESI) m/z: $[M+H]^+$ calcd for $C_5H_9N_2O$ 113.0715, found 113.0714

Spectral data are in good agreement with previously published data.[4]

N,N-dibenzylcyanamide

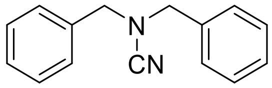

4d

Prepared according to the general procedure **A** using dibenzylamine (99 mg, 0.5 mmol). The crude mixture was extracted to afford product **4d** as colorless oil (106 mg, 0.48 mmol, 95 %).

^{1}H NMR (500 MHz, $CDCl_3$): δ= 7.39 – 7.32 (m, 6H), 7.30 – 7.27 (m, 4H), 4.09 (s, 4H).

^{13}C NMR (126 MHz, $CDCl_3$): δ= 134.5, 129.1, 128.8, 128.8, 118.5, 54.4.

HRMS (ESI) m/z: $[M+H]^+$ calcd for $C_{15}H_{15}N_2$ 223.1235, found 223.1248

Spectral data are in good agreement with previously published data.[3]

7-nitro-3,4-dihydroisoquinoline-2(1H)-carbonitrile

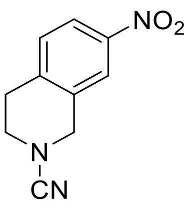

4e

Prepared according to the general procedure **A** using 7-nitro-1,2,3,4-tetrahydroisoquinoline (89 mg, 0.5 mmol). The crude mixture was extracted to afford product **4e** as colorless oil (99 mg, 0.49 mmol, 97 %).

^{1}H NMR (500 MHz,CDCl$_3$): δ= 8.07 (m, 1H), 7.96 (d, J = 2.3 Hz, 1H), 7.33 (d, J = 8.4 Hz, 1H), 4.51 (s, 2H), 3.54 (t, J = 5.8 Hz, 2H), 3.08 (t, J = 5.9 Hz, 2H).

^{13}C NMR (126 MHz,CDCl$_3$): δ= 146.8, 140.3, 132.4, 130.6, 122.4, 121.5, 117.2, 50.0, 46.4, 28.0.

HRMS (ESI) m/z: $[M+H]^+$ calcd for $C_{10}H_{10}N_3O_2$ 204.0773, found 204.0779

7-methyl-3,4-dihydroisoquinoline-2(1H)-carbonitrile

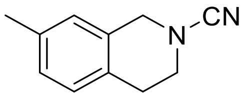

4f

Prepared according to the general procedure **A** using 7-methyl-1,2,3,4-tetrahydroisoquinoline (74 mg, 0.5 mmol). The crude mixture was extracted to afford product **4f** as colorless oil (74 mg, 0.43 mmol, 86 %).

^{1}H NMR (500 MHz,CDCl$_3$): δ= 7.02 (m, 2H), 6.85 (s, 1H), 4.37 (s, 2H), 3.47 (t, J = 5.9 Hz, 2H), 2.91 (t, J = 5.9 Hz, 2H), 2.31 (s, 3H).

^{13}C NMR (126 MHz, CDCl$_3$): δ= 136.5, 130.6, 129.6, 129.1, 128.1, 126.5, 118.2, 50.1, 47.0, 27.4, 21.1.

HRMS (ESI) m/z: $[M+H]^+$ calcd for $C_{11}H_{13}N_2$ 173.1079, found 173.1085

Thiomorpholine-4-carbonitrile

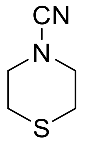

4g

Prepared according to the general procedure **A** using thiomorpholine (52 mg, 0.5 mmol). The crude mixture was extracted to afford product **4g** as colorless oil (63 mg, 0.49 mmol, 98 %).

^{1}H NMR (500 MHz, DMSO-d$_6$): δ= 3.47 – 3.36 (m, 4H), 2.70 – 2.61 (m, 4H).

^{13}C NMR (126 MHz, DMSO-d$_6$): δ= 117.5, 50.3, 25.4.

HRMS (ESI) m/z: $[M+H]^+$ calcd for $C_5H_9N_2S$ 129.0486, found 129.0496

Spectral data are in good agreement with previously published data.[3]

General procedure for the synthesis of thiocyanate derivatives

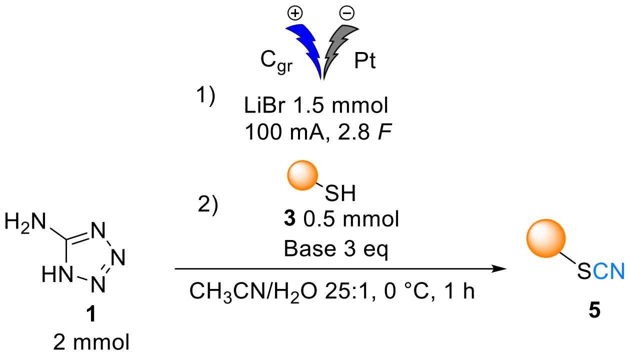

General procedure B: 2 mmol of 5-aminotetrazole (170 mg), 1.5 mmol of LiBr (130 mg) and 5 mL of CH_3CN/H_2O 25:1 are added to a 5 mL one compartment Electrasyn vial. The solution is then electrolyzed with a 41.7 mA.cm^{-2} current density for 2.8 *F* using a carbon graphite anode (WE) and a platinum cathode (CE). At the end of the electrolysis, 0.5 mmol of thiol and 1.5 mmol of Base (Et_3N or DBU) are added dropwise at 0 °C and the solution is stirred at r.t. for 1h. The reaction mixture is then quenched with a saturated aqueous solution of $NaHCO_3$, extracted with AcOEt and dried with Na_2SO_4. The solvent is removed under reduced pressure to afford the desired product without further purification.

(thiocyanatomethyl)benzene

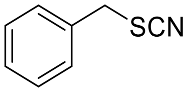

5a

Prepared according to the general procedure **B** using phenylmethanethiol (62 mg, 0.5 mmol) and Et_3N (152 mg, 1.5 mmol). The crude mixture was purified by flash column chromatography to afford product **5a** as colorless oil (62 mg, 0.42 mmol, 83 %).

^{1}H NMR (500 MHz, $CDCl_3$): δ= 7.45 – 7.31 (m, 5H), 4.17 (s, 2H).

^{13}C NMR (126 MHz, $CDCl_3$): δ= 134.5, 129.3, 129.1, 129.1, 112.1, 38.5.

Spectral data are in good agreement with previously published data.[5]

1-chloro-4-thiocyanatobenzene

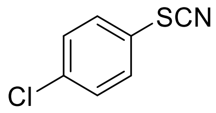

5b

Prepared according to the general procedure **B** using 4-chlorobenzenethiol (72 mg, 0.5 mmol) and DBU (228 mg, 1.5 mmol). The crude mixture was purified by flash column chromatography to afford product **5b** as colorless oil (64 mg, 0.38 mmol, 75 %).

^{1}H NMR (400 MHz, $CDCl_3$): δ= 7.52 – 7.42 (m, 2H), 7.46 – 7.36 (m, 2H).

^{13}C NMR (101 MHz, $CDCl_3$): δ= 136.3, 131.6, 130.6, 122.8, 110.12.

HRMS (ESI) m/z: $[M+H]^+$ calcd for C_7H_5NSCl 169.9831, found 169.9818

Spectral data are in good agreement with previously published data.[6]

1-methoxy-4-thiocyanatobenzene

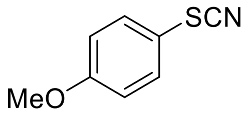

5c

Prepared according to the general procedure **B** using 4-methoxybenzenethiol (70 mg, 0.5 mmol) and DBU (228 mg, 1.5 mmol). The crude mixture was purified by flash column chromatography to afford product **5c** as colorless oil (33 mg, 0.2 mmol, 40 %).

^{1}H NMR (400 MHz, $CDCl_3$): δ= 7.54 – 7.44 (m, 2H), 6.99 – 6.88 (m, 2H), 3.82 (s, 3H).

^{13}C NMR (101 MHz, $CDCl_3$): δ= 161.4, 134.0, 116.0, 114.0, 111.7, 55.6.

HRMS (ESI) m/z: $[M+H]^+$ calcd for C_8H_8NOS 166.0327, found 166.0326

Spectral data are in good agreement with previously published data.[6]

1-thiocyanatododecane

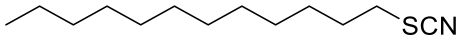

5d

Prepared according to the general procedure **B** using dodecane-1-thiol (101 mg, 0.5 mmol) and Et_3N (152 mg, 1.5 mmol). The crude mixture was purified by flash column chromatography to afford product **5d** as colorless oil (64 mg, 0.28 mmol, 56 %).

^{1}H NMR (400 MHz, $CDCl_3$): δ= 2.96 – 2.89 (m, 2H), 1.80 (m, 2H), 1.41 (m, 2H), 1.35 – 1.19 (m, 16H), 0.86 (t, J = 6.8 Hz, 3H).

^{13}C NMR (101 MHz, $CDCl_3$): δ= 112.6, 34.2, 32.0, 30.0, 29.7, 29.6, 29.5, 29.4, 29.0, 28.1, 22.8, 14.2.

Spectral data are in good agreement with previously published data.[5]

General procedure for the synthesis of heterocycles derivatives

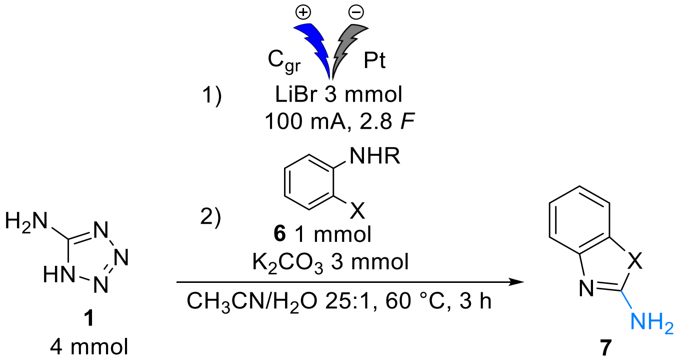

General procedure C: 2 mmol of 5-aminotetrazole (340 mg), 1.5 mmol of LiBr (260 mg) and 5 mL of CH_3CN/H_2O are added to a 5 mL one compartment Electrasyn vial. The solution is then electrolyzed with a 41.7 $mA.cm^{-2}$ current density for 2.8 F using a carbon graphite anode (WE) and a platinum cathode (CE). At the end of the electrolysis, 1 mmol of amine and 3 mmol of K_2CO_3 (414 mg) are added and the solution is

stirred for 3h at 60 °C. The reaction mixture is then quenched with a saturated aqueous solution of $NaHCO_3$, extracted with AcOEt, dried with Na_2SO_4 and the solvent is removed under reduced pressure. The crude mixture is purified via flash column chromatography using Hexane/AcOEt (from 9:1 to 0:10) as eluent to afford the desired product.

Benzo[d]oxazol-2-amine

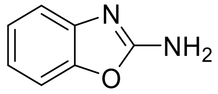

7a

Prepared according to the general procedure **C** using 2-aminophenol (109 mg, 1 mmol). The crude mixture was purified by flash column chromatography to afford product **7a** as yellow oil (107 mg, 0.80 mmol, 80 %).

^{1}H NMR (500 MHz, $CDCl_3$): δ= 7.33 (m, 1H), 7.30 – 7.22 (m, 1H), 7.17 (m, 1H), 7.06 (m, 1H), 6.06 (s, 2H).

^{13}C NMR (126 MHz,$CDCl_3$): δ= 162.3, 148.7, 142.7, 124.1, 121.3, 116.4, 109.1.

HRMS (ESI) m/z: $[M+H]^+$ calcd for $C_7H_7N_2O$ 135.0558, found 135.0558

Spectral data are in good agreement with previously published data.[7]

1H-benzo[d]imidazol-2-amine

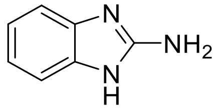

7b

Prepared according to the general procedure **C** using benzene-1,2-diamine (108 mg, 1 mmol). The crude mixture was purified by flash column chromatography to afford product **7b** as brown solid (76 mg, 0.57 mmol, 57 %).

^{1}H NMR (500 MHz, DMSO-d_6): δ= 7.15 – 7.10 (m, 2H), 6.92 – 6.87 (m, 2H), 6.44 (s, 2H).

^{13}C NMR (126 MHz, DMSO-d_6): δ= 155.1, 138.0, 120.1, 112.0.

HRMS (ESI) m/z: $[M+H]^+$ calcd for $C_7H_8N_3$ 134.0718, found 134.0720

Spectral data are in good agreement with previously published data.[8]

Benzo[d]thiazol-2-amine

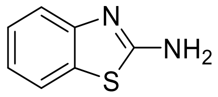

7c

Prepared according to the general procedure **C** using 2-aminobenzenethiol (125 mg, 1 mmol). The crude mixture was purified by flash column chromatography to afford product **7c** as yellow oil (117 mg, 0.78 mmol, 78 %).

^{1}**H NMR** (500 MHz, DMSO-d_6): δ= 7.60 (d, J = 7.6 Hz, 1H), 7.47 (s, 2H), 7.30 (d, J = 7.9 Hz, 1H), 7.16 (t, J = 7.5 Hz, 1H), 6.96 (t, J = 7.4 Hz, 1H).

^{13}**C NMR** (126 MHz, $CDCl_3$): δ= 166.3, 152.1, 131.6, 126.1, 122.4, 121.0, 119.2.

HRMS (ESI) m/z: $[M+H]^+$ calcd for $C_7H_7N_2S$ 151.0330, found 151.0331

Spectral data are in good agreement with previously published data.[9]

3-methylbenzo[d]oxazol-2(3H)-imine

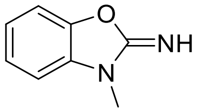

7d

Prepared according to the general procedure **C** using 2-(methylamino)phenol (123 mg, 1 mmol). The crude mixture was purified by flash column chromatography to afford product **7d** as colorless oil (104 mg, 0.70 mmol, 70 %).

^{1}**H NMR** (500 MHz, DMSO-d_6): δ= 7.14 – 7.02 (m, 2H), 6.98 (m, 1H), 6.92 (m, 1H), 3.24 (s, 3H).

^{13}**C NMR** (126 MHz, DMSO-d_6): δ= 155.7,/ 143.8, 133.7, 123.3, 120.4, 108.1, 107.1, 28.3.

HRMS (ESI) m/z: $[M+H]^+$ calcd for $C_8H_9N_2O$ 149.0715, found 149.0715

General procedure for the cyanation of elettron-rich aromatic rings

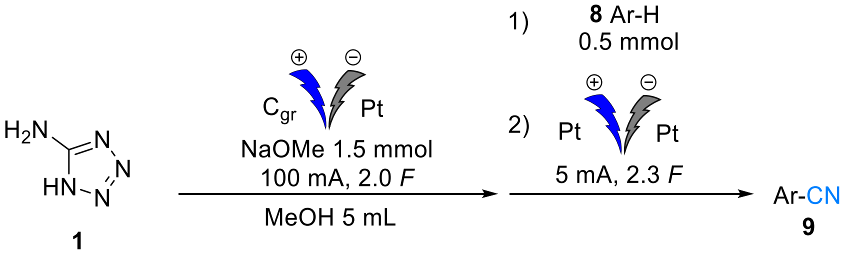

General procedure D: 1.5 mmol of 5-aminotetrazole (128 mg), 1.5 mmol of NaOMe (81 mg) and 5 mL of MeOH are added to a 5 mL one compartment Electrasyn vial. The solution is then electrolyzed with a 41.7 $mA.cm^{-2}$ current density for 2.0 *F* using a carbon graphite anode (WE) and a platinum cathode (CE). At the end of the electrolysis, 0.5 mmol of aromatic compound are added and the solution is electrolyzed with 2.8 $mA.cm^{-2}$ current density for 2.3 *F* using a platinum anode (WE) and a platinum cathode (CE). The reaction mixture is then quenched with a saturated aqueous solution of $NaHCO_3$, extracted with Et_2O, dried with Na_2SO_4 and the solvent is removed under reduced pressure. The crude mixture is purified via flash column chromatography using Hexane/AcOEt (from 9:1 to 4:6) as eluent to afford the desired product.

2,4-dimethoxybenzonitrile

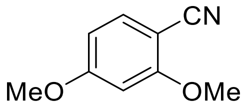

9a

Prepared according to the general procedure **D** using 1,3-dimethoxybenzene (69 mg, 0.5 mmol). The crude mixture was purified by flash column chromatography to afford product **9a** as white solid (68 mg, 0.42 mmol, 83 %).

^{1}H NMR (500 MHz,$CDCl_3$): δ= 7.25 (d, J = 8.6 Hz, 1H), 6.30 (dd, J = 8.6, 2.3 Hz, 1H), 6.24 (d, J = 2.3 Hz, 1H), 3.68 (s, 3H), 3.64 (s, 3H).

^{13}C NMR (126 MHz, $CDCl_3$): δ= 164.8, 163.0, 135.0, 117.1, 105.9, 98.6, 94.1, 56.1, 55.8.

HRMS (ESI) m/z: $[M+H]^+$ calcd for $C_9H_{10}NO_2$ 164.0712, found 164.0711

Spectral data are in good agreement with previously published data.[10]

2-bromo-4,6-dimethoxybenzonitrile

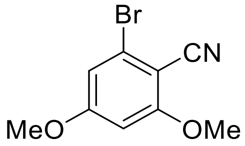

9b

Prepared according to the general procedure **D** using 1-bromo-3,5-dimethoxybenzene (109 mg, 0.5 mmol). The crude mixture was purified by flash column chromatography to afford product **9b** as white solid (56 mg, 0.23 mmol, 46 %).

^{1}H NMR (500 MHz, $CDCl_3$): δ= 6.75 (d, J = 2.1 Hz, 1H), 6.40 (d, J = 2.2 Hz, 1H), 3.89 (s, 3H), 3.85 (s, 3H).

^{13}C NMR (126 MHz, $CDCl_3$): δ= 164.4, 164.0, 127.5, 115.4, 110.3, 98.3, 97.7, 56.5, 56.2.

HRMS (ESI) m/z: $[M+H]^+$ calcd for $C_9H_9NO_2Br$ 241.9817, found 241.9837

Spectral data are in good agreement with previously published data.[11]

2-methoxybenzonitrile/ 4-methoxybenzonitrile

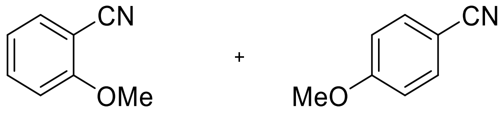

9c **9c'**

Prepared according to the general procedure **D** using anisole (54 mg, 0.5 mmol). The crude mixture was purified by flash column chromatography to afford product **9c** as mixture of isomers (*o*:*p* 1:1.2) and as colorless oil (29 mg, 0.22 mmol, 44 %).

^{1}H NMR (400 MHz, $CDCl_3$): δ= 7.63 – 7.53 (m, 4H, major + minor), 7.06 – 6.95 (m, 4H, major + minor), 3.95 (s, 3H, minor), 3.88 (s, 3H, major).

^{13}C NMR (101 MHz, $CDCl_3$): δ= 163.0, 161.4, 134.5, 134.1, 133.9, 120.9, 119.4, 116.6, 114.9, 111.4, 104.1, 101.9, 56.1, 55.7.

HRMS (ESI) m/z: $[M+H]^+$ calcd for C_8H_8NO 134.0606, found 134.0619

Spectral data are in good agreement with previously published data.[11]

2-(allyloxy)benzonitrile/ 4-(allyloxy)benzonitrile

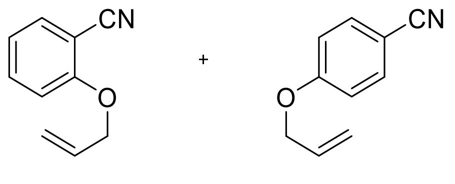

9d **9d'**

Prepared according to the general procedure **D** using (allyloxy)benzene (67 mg, 0.5 mmol). The crude mixture was purified by flash column chromatography to afford product **9d** as mixture of isomers (*o*:*p* 1:1.5) and as colorless oil (38 mg, 0.24 mmol, 48 %).

^{1}H NMR (500 MHz,$CDCl_3$): δ= 7.65 – 7.48 (m, 4H, major + minor), 7.04 – 6.96 (m, 4H, major + minor), 6.06 (m, 2H, major + minor), 5.47 (m, 1H, minor), 5.35 (m, 1H, major), 4.68 (dt, J = 5.0, 1.6 Hz, 1H, minor), 4.61 (dt, J = 5.3, 1.5 Hz, 1H, major).

^{13}C NMR (126 MHz,$CDCl_3$): δ= 162.0, 160.4, 134.3, 134.1, 134.0, 132.2, 132.0, 121.0, 119.3, 118.6, 118.4, 116.6, 115.6, 114.9, 112.8, 104.2, 102.4, 69.6, 69.1.

HRMS (ESI) m/z: $[M+H]^+$ calcd for $C_{10}H_{10}NO$ 160.0762, found 160.0777

Spectral data are in good agreement with previously published data.[11]

2,6-dimethoxynicotinonitrile

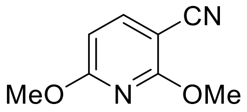

9e

Prepared according to the general procedure **D** using 2,6-dimethoxypyridine (70 mg, 0.5 mmol).). The crude mixture was purified by flash column chromatography to afford product **9e** as white solid (66 mg, 0.40 mmol, 80 %).

^{1}H NMR (400 MHz, $CDCl_3$): δ= 7.67 (d, J = 8.3 Hz, 1H), 6.33 (d, J = 8.3 Hz, 1H), 4.01 (s, 3H), 3.95 (s, 3H).

^{13}C NMR (101 MHz, $CDCl_3$): δ= 165.8, 164.9, 144.3, 116.3, 102.9, 86.8, 54.4, 54.2.

HRMS (ESI) m/z: $[M+H]^+$ calcd for $C_8H_9N_2O_2$ 165.0664, found 165.0680

Spectral data are in good agreement with previously published data.[11]

2,4,6-trimethoxybenzonitrile

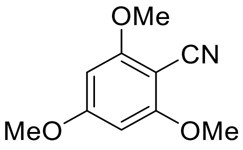

9f

Prepared according to the general procedure **D** using 1,3,5-trimethoxybenzene (84 mg, 0.5 mmol) with 25 mA. The crude mixture was purified by flash column chromatography to afford product **9f** as white solid (30 mg, 0.16 mmol, 31 %).

^{1}H NMR (400 MHz, $CDCl_3$): δ= 6.05 (s, 2H), 3.86 (s, 6H), 3.83 (s, 3H).

^{13}C NMR (101 MHz, $CDCl_3$): δ= 165.5, 163.9, 114.7, 90.5, 84.2, 56.2, 55.8.

HRMS (ESI) m/z: $[M+H]^+$ calcd for $C_{10}H_{12}NO_3$ 194.0817, found 194.0824

Spectral data are in good agreement with previously published data.[12]

3-methylbenzo[b]thiophene-2-carbonitrile

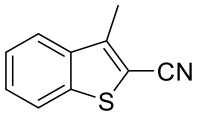

9g

Prepared according to the general procedure **D** using 3-methylbenzo[b]thiophene (74 mg, 0.5 mmol). The crude mixture was purified by flash column chromatography to afford product **9g** as colorless oil (45 mg, 0.26 mmol, 52 %).

^{1}H NMR (500 MHz, $CDCl_3$): δ= 7.87 – 7.76 (m, 2H), 7.51 (m, 2H), 2.64 (s, 3H).

^{13}C NMR (126 MHz, $CDCl_3$): δ= 145.3, 141.0, 137.7, 128.0, 125.5, 123.7, 122.8, 114.7, 105.8, 13.9.

HRMS (ESI) m/z: $[M+H]^+$ calcd for $C_{10}H_8NS$ 174.0377, found 174.0387

Spectral data are in good agreement with previously published data.[11]

2-(methyl(phenyl)amino)acetonitrile

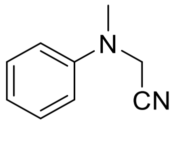

9h

Prepared according to the general procedure **D** using *N,N*-dimethylaniline (61 mg, 0.5 mmol). The crude mixture was purified by flash column chromatography to afford product **9h** as colorless oil (26 mg, 0.18 mmol, 36 %).

^{1}H NMR (500 MHz, $CDCl_3$): δ= 7.36 – 7.26 (m, 2H), 6.98 – 6.88 (m, 1H), 6.90 – 6.84 (m, 2H), 4.17 (s, 2H), 3.01 (s, 3H).

^{13}C NMR (126 MHz, $CDCl_3$): δ= 147.9, 129.6, 120.3, 115.6, 115.0, 42.4, 39.4.

Spectral data are in good agreement with previously published data.[13]

General procedure for the cyanation reactions on aldehydes

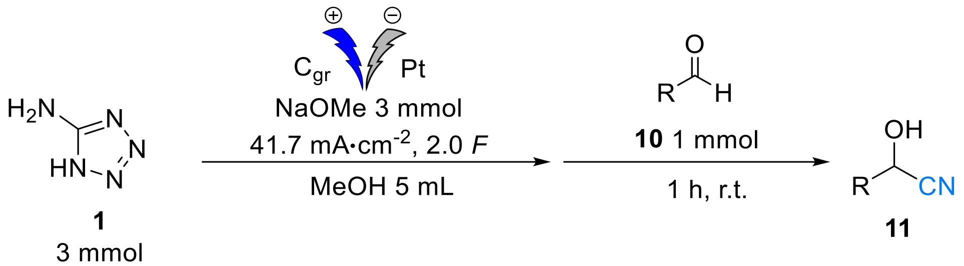

General procedure E: 3 mmol of 5-aminotetrazole (255 mg), 3 mmol of NaOMe (162 mg) and 5 mL of MeOH are added to a 5 mL one compartment Electrasyn vial. The solution is then electrolyzed with a 41.7 $mA.cm^{-2}$ current density for 2.0 *F* using a carbon graphite anode (WE) and a platinum cathode (CE). At the end of the electrolysis, 1 mmol of aldehyde are added and the solution is stirred at r.t, for 1h. The reaction mixture is then quenched with a saturated aqueous solution of $NaHCO_3$, extracted with AcOEt, dried with Na_2SO_4 and the solvent is removed under reduced pressure. The crude mixture is purified via flash column chromatography using Hexane/AcOEt as eluent to afford the desired product. (**Caution:** the product obtained could generate HCN)

2-hydroxy-4-phenylbutanenitrile

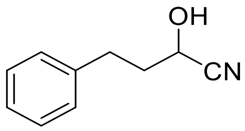

11a

Prepared according to the general procedure **E** using 3-phenylpropanal (134 mg, 1 mmol). The crude mixture was purified by flash column chromatography to afford product **11a** as colorless oil (135 mg, 0.84 mmol, 84 %).

^{1}H NMR (400 MHz, $CDCl_3$): δ= 7.33 – 7.23 (m, 2H), 7.25 – 7.13 (m, 3H), 4.37 (m, 1H), 3.25 (d, J = 6.0 Hz, 1H), 2.80 (m, 2H), 2.20 – 2.08 (m, 2H).

^{13}C NMR (101 MHz, $CDCl_3$): δ= 139.7, 128.9, 128.6, 126.7, 120.1, 60.5, 36.6, 30.8.

HRMS (ESI) m/z: $[M+H]^+$ calcd for $C_{10}H_{12}NO$ 162.0919, found 162.0929

Spectral data are in good agreement with previously published data.[14]

2-hydroxyundecanenitrile

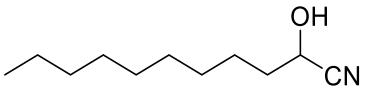

11b

Prepared according to the general procedure **E** using decanal (156 mg, 1 mmol). The crude mixture was purified by flash column chromatography to afford product **11b** as colorless oil (130 mg, 0.71 mmol, 71 %).

^{1}H NMR (500 MHz, $CDCl_3$): δ= 4.44 (t, J = 6.8 Hz, 1H), 3.06 (s, 1H), 1.89 – 1.77 (m, 2H), 1.53 – 1.41 (m, 2H), 1.39 – 1.18 (m, 12H), 0.91 – 0.81 (m, 3H).

^{13}C NMR (126 MHz, $CDCl_3$): δ= 120.3, 61.4, 35.2, 32.0, 29.5, 29.5, 29.3, 29.0, 24.7, 22.8, 14.2.

HRMS (ESI) m/z: $[M+H]^+$ calcd for $C_{11}H_{22}NO$ calcd 184.1701, found 184.1720

Spectral data are in good agreement with previously published data.[15]

2-hydroxy-3-phenylpropanenitrile

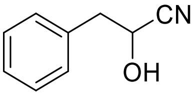

11c

Prepared according to the general procedure **E** using 2-phenylacetaldehyde (120 mg, 1 mmol). The crude mixture was purified by flash column chromatography to afford product **11c** as colorless oil (100 mg, 0.68 mmol, 68 %).

^{1}H NMR (500 MHz, $CDCl_3$): δ= 7.41 – 7.21 (m, 5H), 4.59 (t, J = 6.6 Hz, 1H), 3.09 (d, J = 6.7 Hz, 2H).

^{13}C NMR (126 MHz, $CDCl_3$): δ= 134.1, 129.8, 129.0, 127.8, 119.6, 62.2, 41.3.

HRMS (ESI) m/z: $[M+H]^+$ calcd for $C_9H_{10}NO$ 148.0762, found 148.0763

Spectral data are in good agreement with previously published data.[16]

2-hydroxynonanenitrile

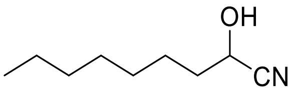

11d

Prepared according to the general procedure **E** using octanal (128 mg, 1 mmol). The crude mixture was purified by flash column chromatography to afford product **11d** as colorless oil (115 mg, 0.74 mmol, 74 %).

^{1}H NMR (400 MHz, $CDCl_3$): δ= 4.44 (t, J = 6.8 Hz, 1H), 3.46 (s, 1H), 1.87 – 1.72 (m, 2H), 1.57 – 1.38 (m, 2H), 1.38 – 1.15 (m, 8H), 0.92 – 0.77 (m, 3H).

^{13}C NMR (101 MHz, $CDCl_3$): δ= 120.3, 61.3, 35.2, 31.8, 29.1, 29.0, 24.7, 22.7, 14.1.

HRMS (ESI) m/z: $[M+NH_4]^+$ calcd for $C_9H_{21}N_2O$ 173.1654, found 173.1670

Spectral data are in good agreement with previously published data.[17]

2-hydroxy-2-phenylacetonitrile

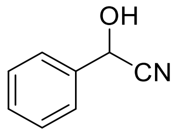

11e

Prepared according to the general procedure **E** using benzaldehyde (106 mg, 1 mmol). The crude mixture was purified by flash column chromatography to afford product **11e** as colorless oil (90 mg, 0.68 mmol, 68 %).

^{1}H NMR (400 MHz, $CDCl_3$): δ= 7.51 – 7.45 (m, 2H), 7.41 (m, 3H), 5.47 (s, 1H), 4.00 (s, 1H).

^{13}C NMR (101 MHz, $CDCl_3$): δ= 135.3, 129.9, 129.3, 126.8, 119.1, 63.5.

HRMS (ESI) m/z: $[M+H]^+$ calcd for C_8H_8NO 134.0606, found 134.0620

Spectral data are in good agreement with previously published data.[18]

benzyl (3-cyano-3-hydroxypropyl)carbamate

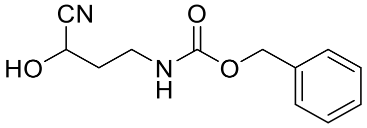

11f

Prepared according to the general procedure **E** using benzyl (3-cyano-3-hydroxypropyl)carbamate (207 mg, 1 mmol). The crude mixture was purified by flash column chromatography to afford product **11f** as colorless oil (170 mg, 0.73 mmol, 73 %).

^{1}H NMR (500 MHz, $CDCl_3$): δ= 7.33 (m, 5H), 5.38 (t, J = 6.3 Hz, 1H), 5.09 (s, 2H), 4.81 (s, 1H), 4.50 (m, 1H), 3.46 – 3.28 (m, 2H), 1.97 (m, 2H).

^{13}C NMR (126 MHz, $CDCl_3$): δ= 157.7, 136.1, 128.7, 128.4, 128.2, 120.0, 67.4, 58.6, 36.5, 35.3.

HRMS (ESI) m/z: $[M+H]^+$ calcd for $C_{12}H_{15}N_2O_3$ 235.1083, found 235.1097

General procedure for the cyanation reactions on electrophilic compounds

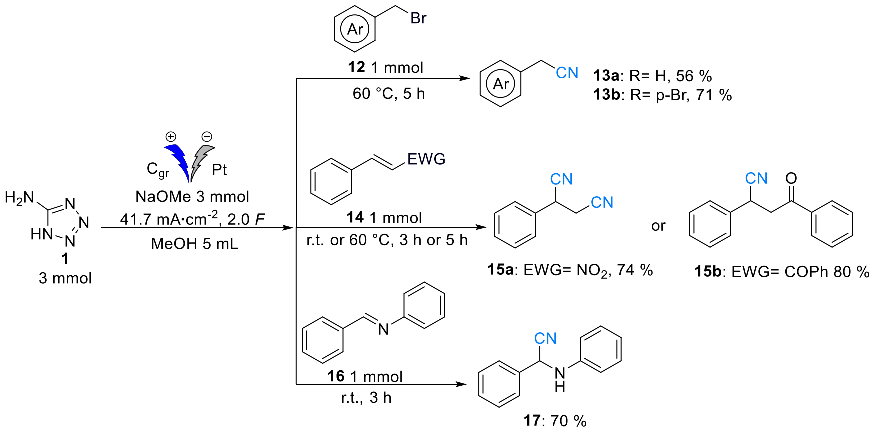

General procedure F: 3 mmol of 5-aminotetrazole (255 mg), 3 mmol of NaOMe (162 mg) and 5 mL of MeOH are added to a 5 mL one compartment Electrasyn vial. The solution is then electrolyzed with a 41.7 $mA.cm^{-2}$ current density for 2.0 *F* using a carbon graphite anode (WE) and a platinum cathode (CE). At the end of the electrolysis, 1 mmol of electrophile are added and the solution is stirred at r.t. or 60 °C for 3h-5h. The reaction mixture is then quenched with a saturated aqueous solution of $NaHCO_3$, extracted with AcOEt, dried with Na_2SO_4 and the solvent is removed under reduced pressure. The crude mixture is purified via flash column chromatography using Hexane/AcOEt as eluent to afford the desired product.

2-phenylacetonitrile

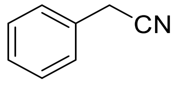

13a

Prepared according to the general procedure **F** using (bromomethyl)benzene (171 mg, 1 mmol) at 60 °C for 5h. The crude mixture was purified by flash column chromatography to afford product **13a** as colorless oil (66 mg, 0.56 mmol, 56 %).

^{1}H NMR (500 MHz, $CDCl_3$): δ= 7.44 – 7.27 (m, 5H), 3.74 (s, 2H).

^{13}C NMR (126 MHz, $CDCl_3$): δ= 130.0, 129.2, 128.1, 128.0, 118.0, 23.7.

HRMS (ESI) m/z: $[M+H]^+$ calcd for C_8H_8N calcd 118.0657, found 118.0667

Spectral data are in good agreement with previously published data.[19]

2-(4-bromophenyl)acetonitrile

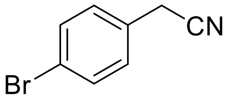

13b

Prepared according to the general procedure **F** using 1-bromo-4-(bromomethyl)benzene (250 mg, 1 mmol) at 60 °C for 5h. The crude mixture was purified by flash column chromatography to afford product **13b** as colorless oil (139 mg, 0.71 mmol, 71 %).

^{1}H NMR (500 MHz, $CDCl_3$): δ= 7.52 – 7.41 (m, 2H), 7.23 – 7.12 (m, 2H), 3.69 (s, 2H)

^{13}C NMR (126 MHz, $CDCl_3$): δ= 132.3, 129.7, 129.0, 122.2, 117.4, 23.2.

HRMS (ESI) m/z: $[M+H]^+$ calcd for C_8H_7NBr calcd 195.9762, found 195.9774

Spectral data are in good agreement with previously published data.[20]

2-phenylsuccinonitrile

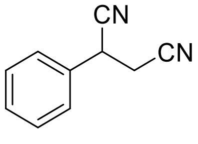

15a

Prepared according to the general procedure **F** using 2-nitrovinylbenzene (149 mg, 1 mmol) at r.t. for 3h. The crude mixture was purified by flash column chromatography to afford product **15a** as yellow solid (115 mg, 0.74 mmol, 74 %).

^{1}H NMR (500 MHz, $CDCl_3$): δ= 7.32 – 7.23 (m, 5H), 4.02 (t, *J* = 6.8 Hz, 1H), 2.87 – 2.74 (m, 2H).

^{13}C NMR (126 MHz, $CDCl_3$): δ= 132.3, 129.7, 129.6, 127.3, 118.0, 115.5, 34.0, 24.7.

HRMS (ESI) m/z: $[M-H]^-$ calcd for $C_{10}H_7N_2$ 155.0609, found 155.0618

Spectral data are in good agreement with previously published data.[21]

4-oxo-2,4-diphenylbutanenitrile

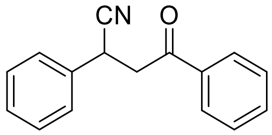

15b

Prepared according to the general procedure **F** using chalcone (208 mg, 1 mmol) at 60 °C for 5h. The crude mixture was purified by flash column chromatography to afford product **15b** as yellow solid (188 mg, 0.80 mmol, 80 %).

^{1}H NMR (400 MHz, $CDCl_3$): δ= 7.95 – 7.89 (m, 2H), 7.62 – 7.55 (m, 1H), 7.48 – 7.30 (m, 7H), 4.55 (dd, J = 8.0, 5.9 Hz, 1H), 3.72 (dd, J = 18.0, 8.0 Hz, 1H), 3.50 (dd, J = 18.0, 5.9 Hz, 1H).

^{13}C NMR (101 MHz, $CDCl_3$): δ= 194.8, 135.7, 135.4, 134.1, 129.4, 129.0, 128.5, 128.2, 127.6, 120.8, 44.6, 32.0.

HRMS (ESI) m/z: $[M+H]^+$ calcd for $C_{16}H_{14}NO$ 236.1075, found 236.1091

Spectral data are in good agreement with previously published data.[22]

2-phenyl-2-(phenylamino)acetonitrile

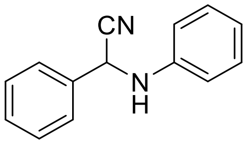

17

Prepared according to the general procedure **F** using (E)-*N*,1-diphenylmethanimine (181 mg, 1 mmol) at r.t. for 3h. The crude mixture was purified by flash column chromatography to afford product **17** as white solid (146 mg, 0.70 mmol, 70 %).

^{1}H NMR (500 MHz, $CDCl_3$): δ= 7.62 – 7.51 (m, 2H), 7.49 – 7.37 (m, 3H), 7.31 – 7.18 (m, 2H), 6.89 (m, 1H), 6.80 – 6.69 (m, 2H), 5.40 (s, 1H), 4.06 (s, 1H).

^{13}C NMR (126 MHz, $CDCl_3$): δ= 144.8, 134.0, 129.6, 129.6, 129.4, 127.3, 120.3, 118.3, 114.3, 50.3.

HRMS (ESI) m/z: $[M+H]^+$ calcd for $C_{14}H_{13}N_2$ 209.1079, found 209.1097

Spectral data are in good agreement with previously published data.[23]

References

1) Schmidt, B.; Schröder, B.; Sonnenberg, K.; Steinhauer, S.; Riedel, S. *Angew. Chem. Int. Ed.* **2019**, *58*, 10340–10344.
2) Fukumoto, K.; Oya, T.; Itazaki, M.; Nakazawa, H. *J. Am. Chem. Soc.* **2008**, *1*, 38–39.
3) Teng, F.; Yu, J. -T.; Jiang, Y.; Yang, H.; Cheng, J. *Chem. Commun.* **2014**, *50*, 8412–8415.
4) Al-Awadi, N. A.; Abdelkhalik, M. M.; El-Dusouqui O. M. E.; Elnagdi, M. H. *J. Heterocyclic Chem.* **2010**, *47*, 207.
5) Frei, R.; Courant, T.; Wodrich, M. D.; Waser, J. *Chem. Eur. J.* **2014**, *21*, 2662–2668.
6) Grundke, C.; Groß, J.; Vierengel, N.; Sirleaf, J.; Schmitz, M.; Krieger, L.; Opatz, T. *Org. Biomol. Chem.* **2023**, *21*, 644–650.
7) Loreau, O.; Camus, N.; Taran, F.; Audisio, D. *Synlett* **2016**, *12*, 1798–1802.
8) Vlaar, T.; Cioc, R. C.; Mampuys, P.; Maes, B. U. W.; Orru, R. V. A.; Ruijter, E. *Angew. Chem. Int. Ed.* **2012**, *52*, 13058–13061.
9) Ramana, T.; Saha, P.; Das, M.; Punniyamurthy, T. *Org. Lett.* **2010**, *12*, 84–87.
10) Shen, T.; Wang, T.; Qin, C.; Jiao, N. *Angew. Chem. Int. Ed.* **2013**, *52*, 6677–6680.
11) Hayrapetyan, D.; Rit, R. K.; Kratz, M.; Tschulik, K.; Gooßen, L. J. *Chem. Eur. J.* **2018**, *24*, 11288–11291.
12) Talavera, G.; Peña, J.; Alcarazo, M. *J. Am. Chem. Soc.* **2015**, *27*, 8704–8707.
13) Zhang, C.; Liu, C.; Shao, Y.; Bao, X.; Wan, X. *Chem. Eur. J.* **2013**, *19*, 17917–17925.
14) Scharfbier, J.; Hazrati, H.; Irran, E.; Oestreich, M. *Org. Lett.* **2017**, *19*, 6562–6565.
15) Błocka, E.; Bosiak, M. J.; Wełniak, M.; Ludwiczak, A.; Wojtczak, A. *Tetrahedron Asymmetry* **2014**, *25*, 554–562.
16) Dickschat, A.; Studer, A. *Org. Lett.* **2010**, *12*, 3972–3974.
17) Kondoh, A.; Arlt, A.; Gabor, B.; Fürstner, A. *Chem. Eur. J.* **2013**, *19*, 7731–7738.
18) Pavel, B.; Jan, O.; David. S. *RSC Adv.* **2020**, *10*, 25029–25045.
19) Shipilovskikh, S. A.; Vaganov, V. Y.; Denisova, E. I.; Rubtsov, A. E.; Malkov, A. V. *Org. Lett.* **2018**, *20*, 728–731.
20) Ezawa, M.; Togo, H. *Eur. J. Org. Chem.* **2017**, *16*, 2379–2384.
21) Kiyokawa, K.; Nagata, T.; Hayakawa, J.; Minakata, S. *Chem. Eur. J.* **2015**, *21*, 1280–1285.
22) Kim, S. H.; Kim, K. H.; Kim, J. N. *Adv. Synth. Cat.* **2011**, *18*, 3335–3339.
23) Grundke, C.; Opatz, T. *Green Chem.* **2019**, *21*, 2362–2366.

NMR Spectra

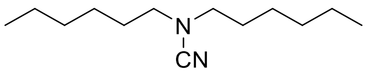

4a

^{1}H NMR, 400 MHz, $CDCl_3$

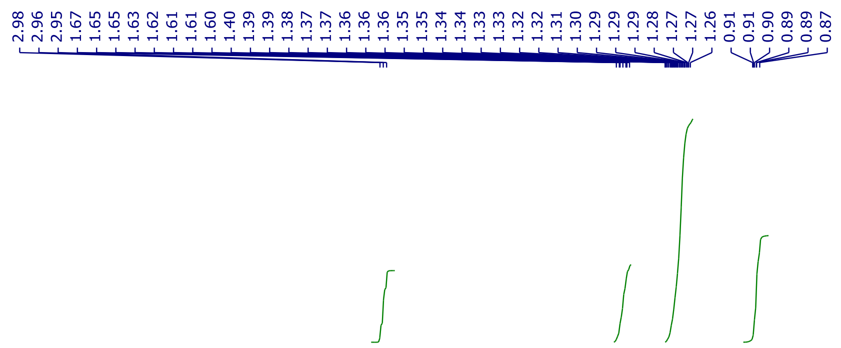

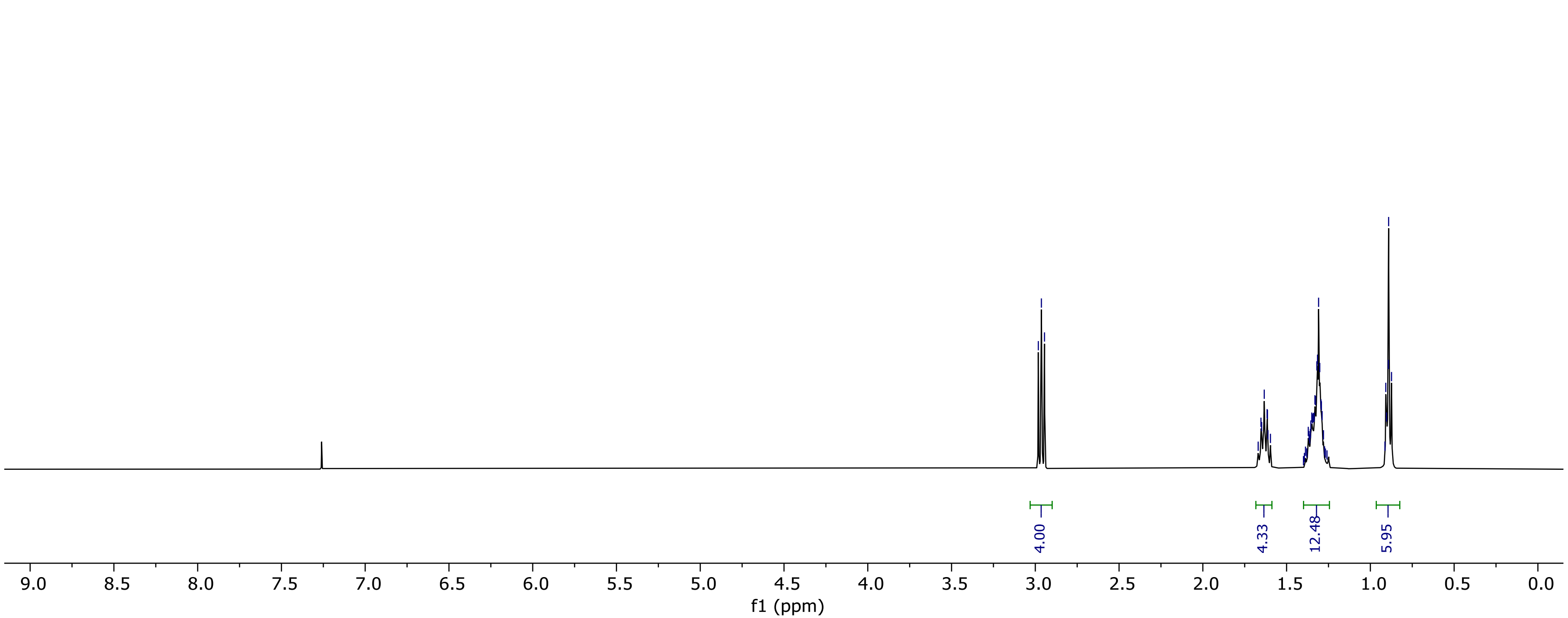

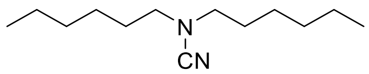

4a

^{13}C NMR, 101 MHz, $CDCl_3$

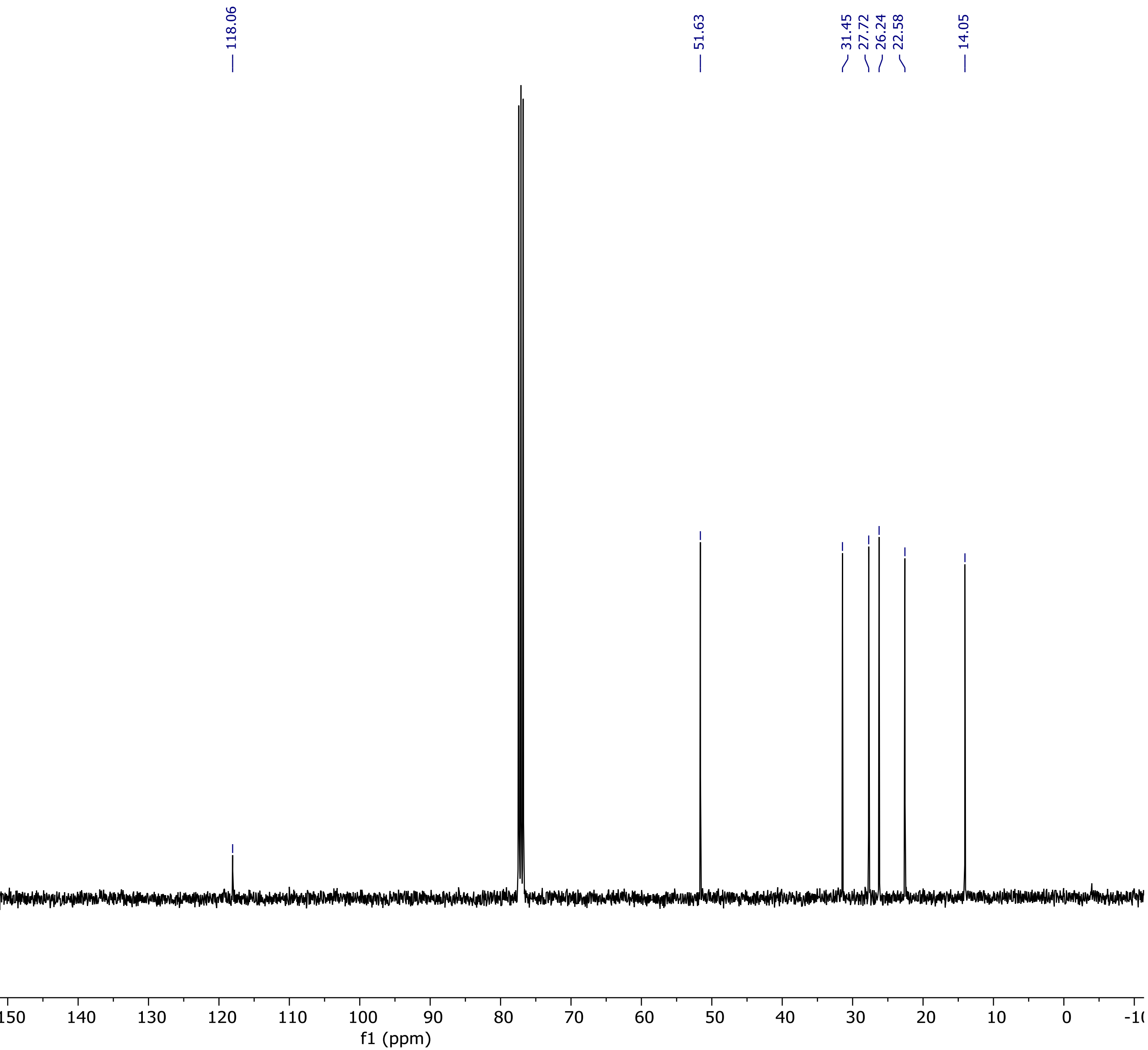

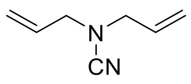

4b

^{1}H NMR, 500 MHz, $CDCl_3$

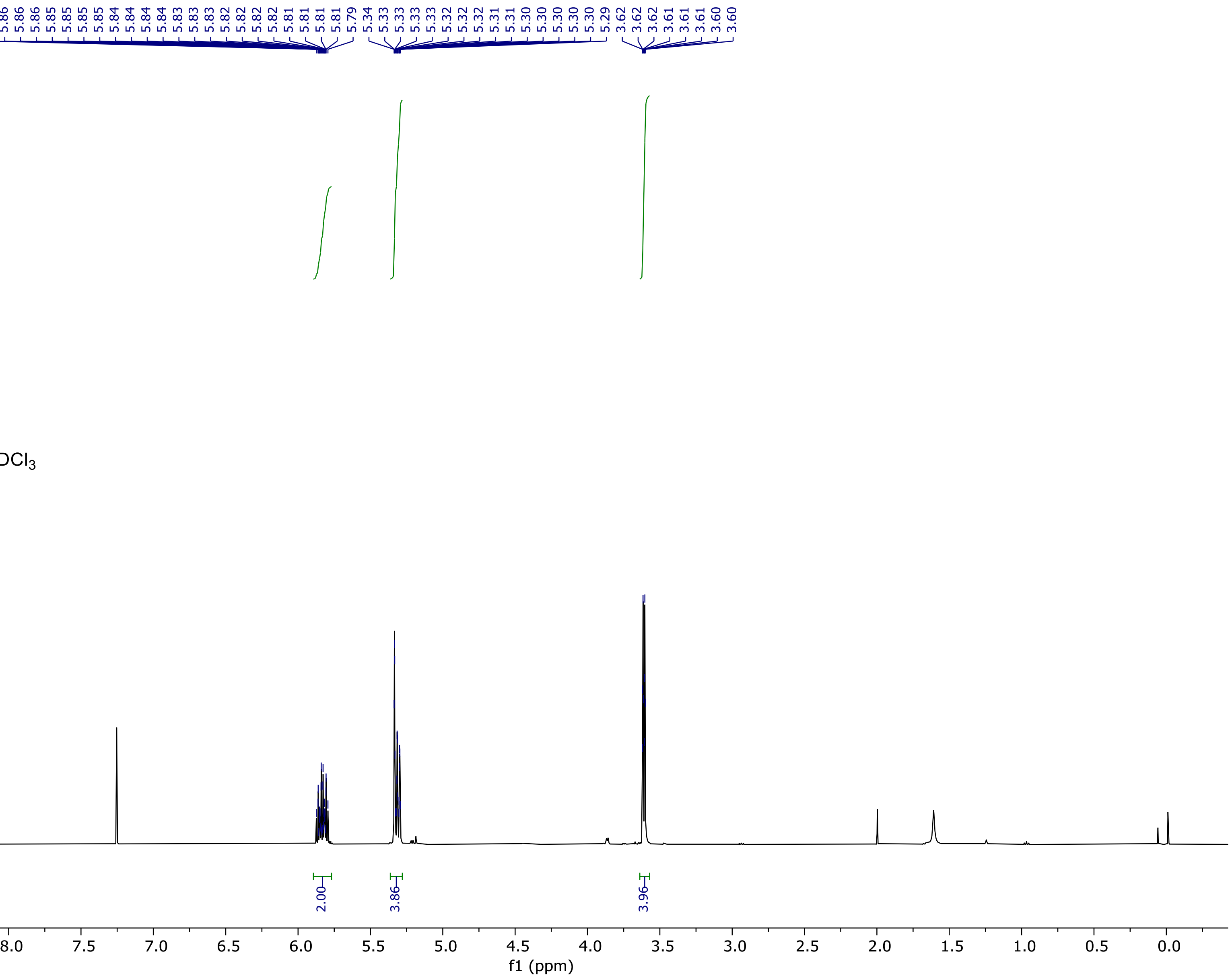

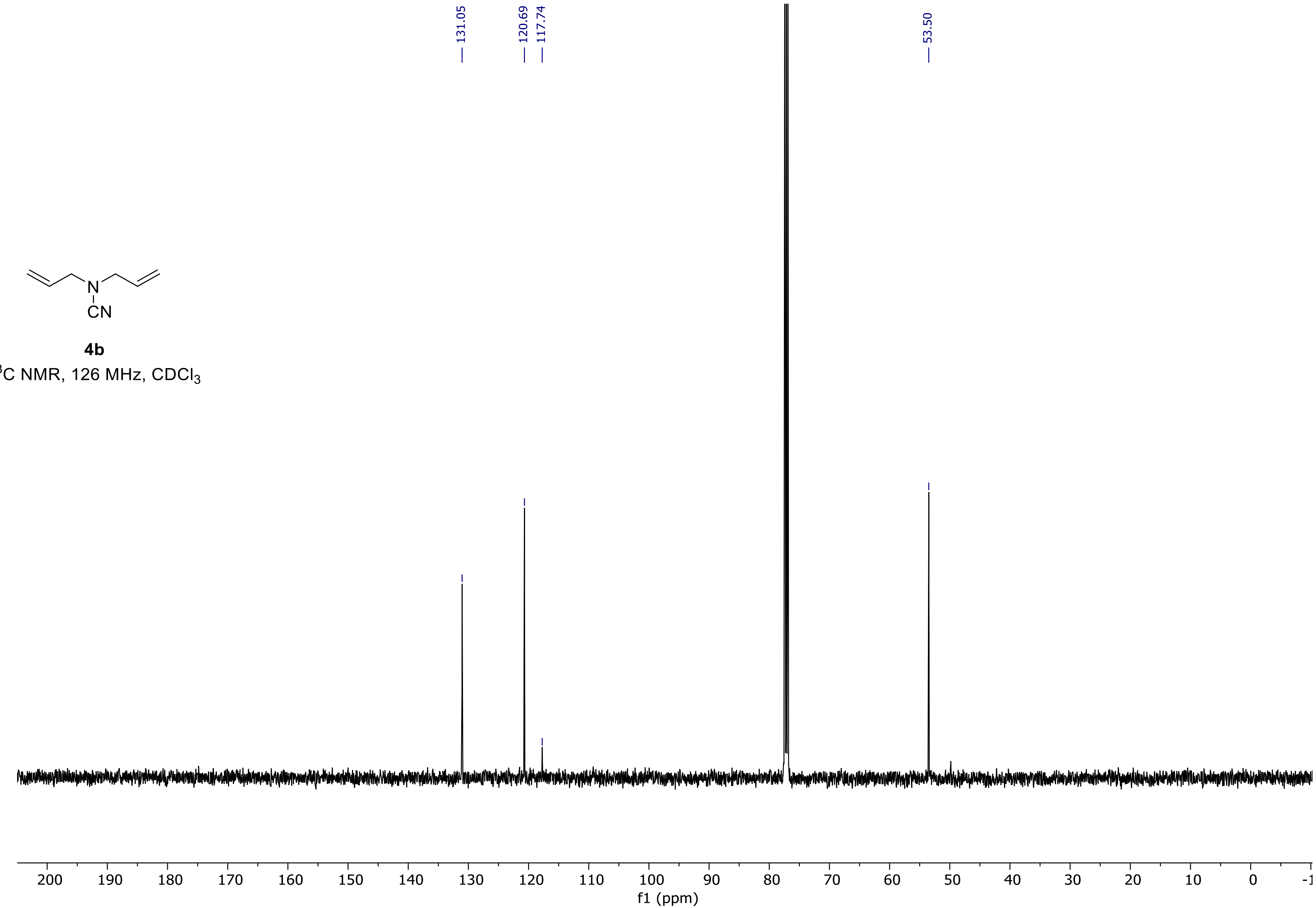

4b

^{13}C NMR, 126 MHz, $CDCl_3$

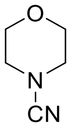

4c

^{1}H NMR, 400 MHz, $CDCl_3$

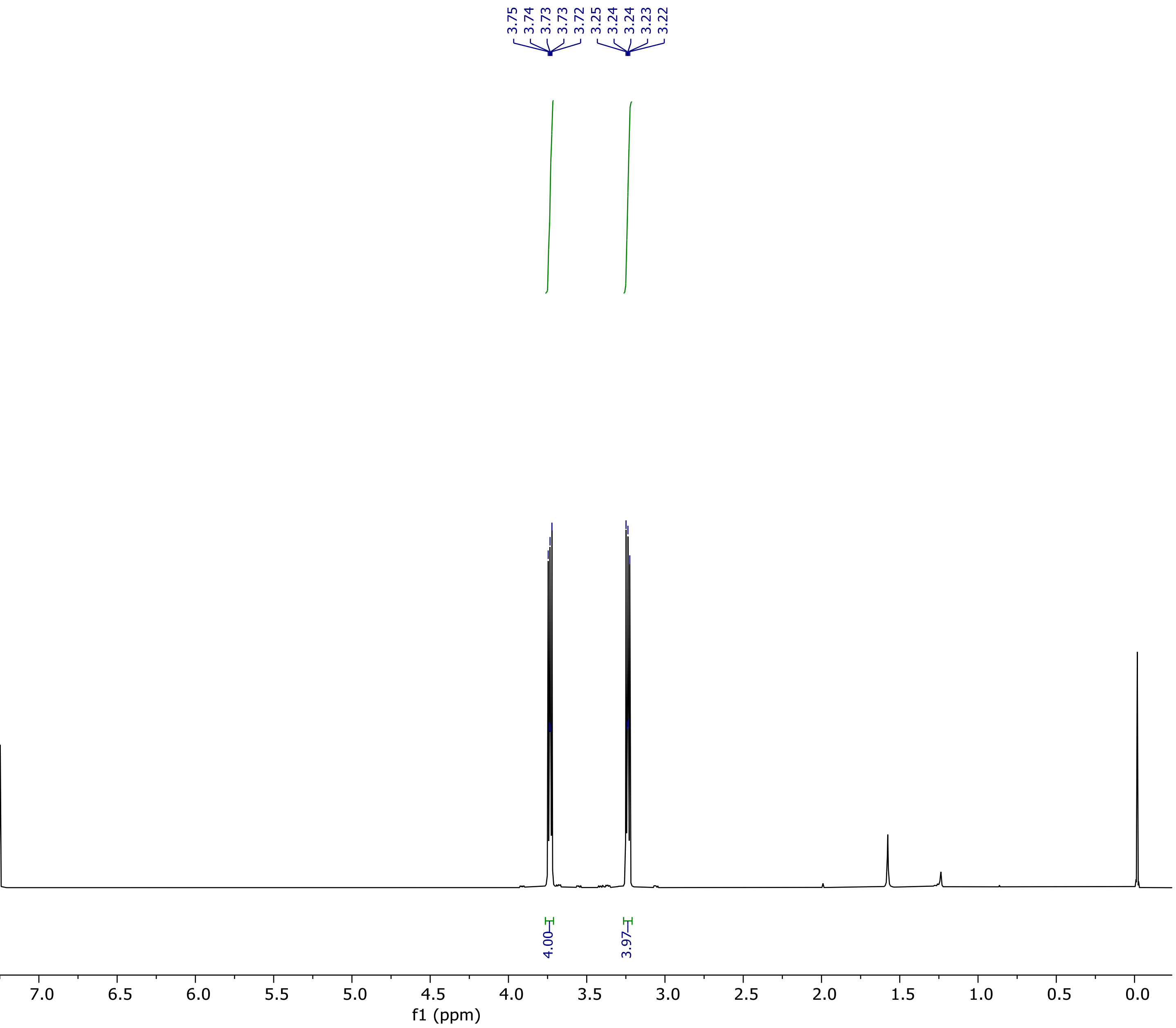

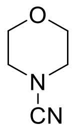

4c

^{13}C NMR, 101 MHz, $CDCl_3$

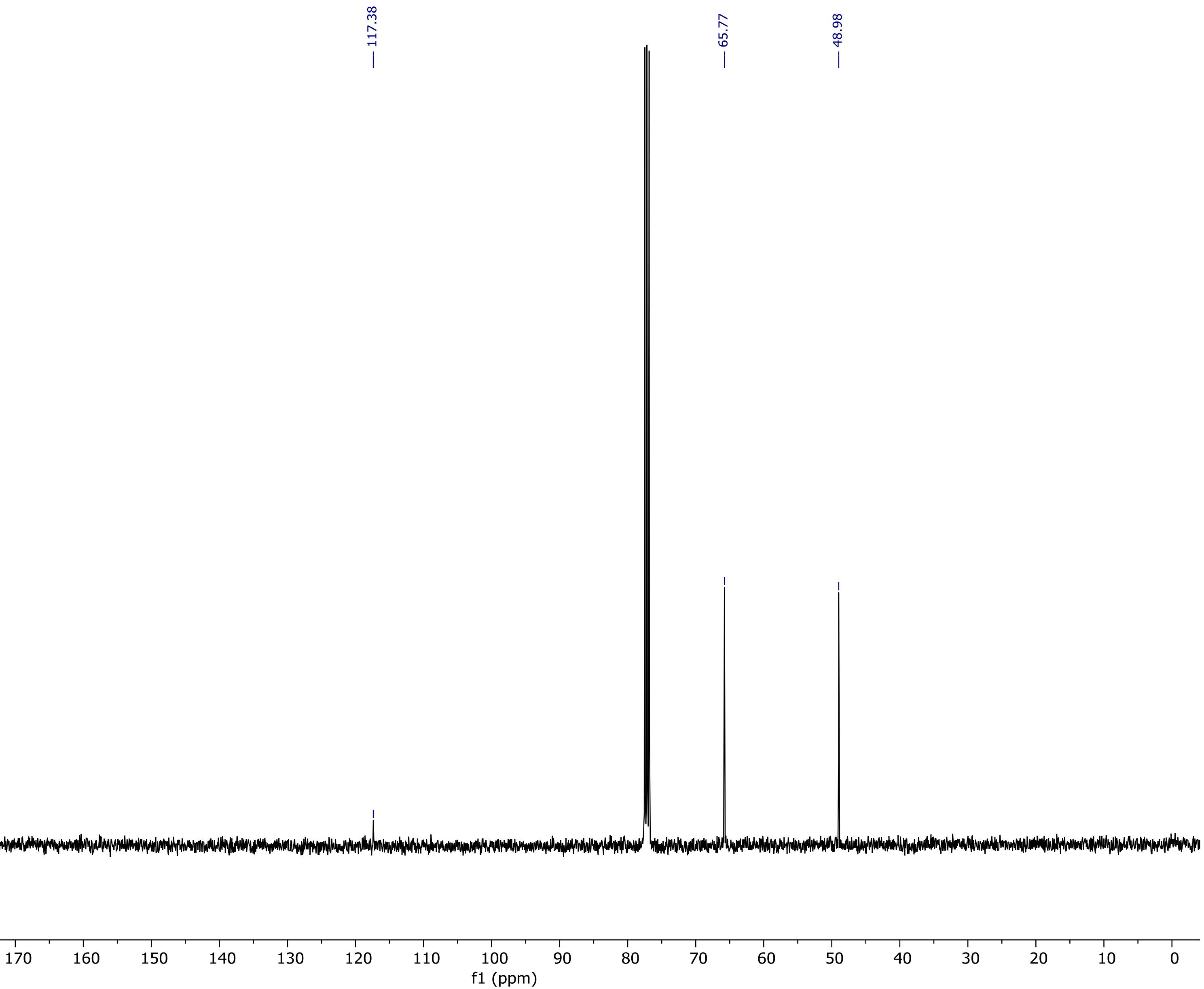

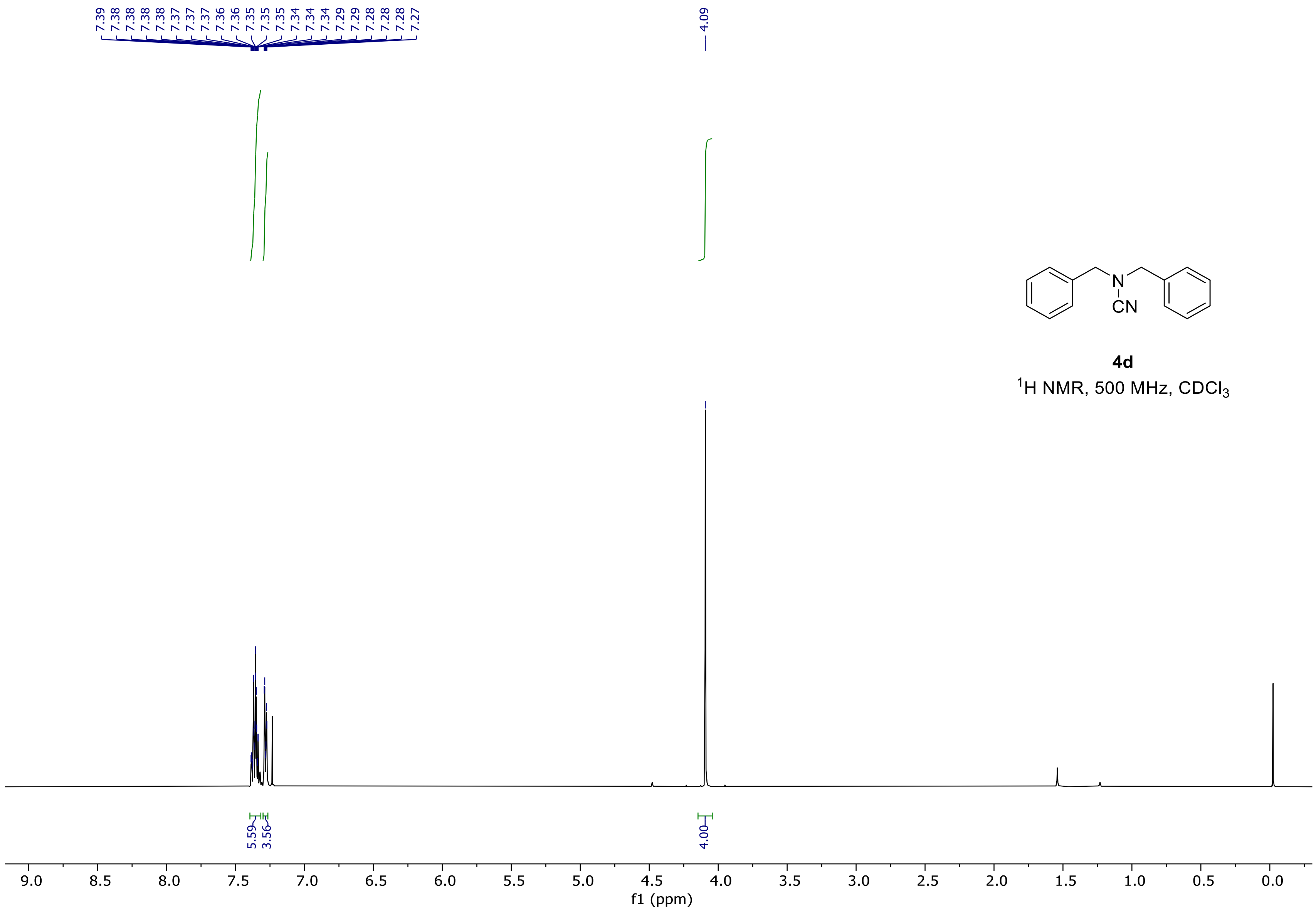
7.39 7.38 7.38 7.38 7.38 7.37 7.37 7.37 7.36 7.36 7.35 7.35 7.35 7.34 7.34 7.34 7.29 7.29 7.28 7.28 7.28 7.27
4.09
N
CN
4d
1H NMR, 500 MHz, CDCl3
5.59
3.56
4.00
9.0 8.5 8.0 7.5 7.0 6.5 6.0 5.5 5.0 4.5 4.0 3.5 3.0 2.5 2.0 1.5 1.0 0.5 0.0
f1 (ppm)

4d
^{13}C NMR, 126 MHz, $CDCl_3$

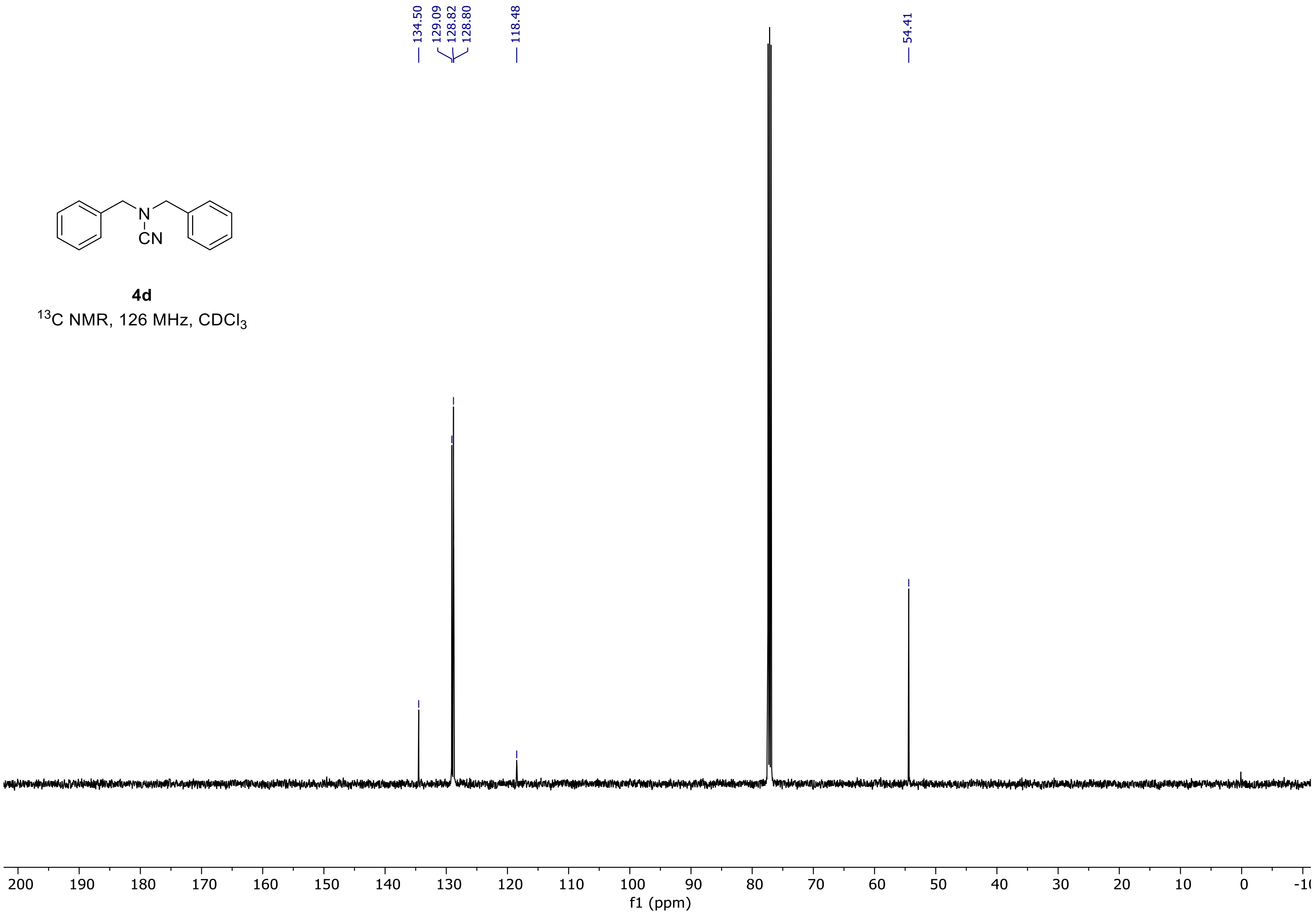

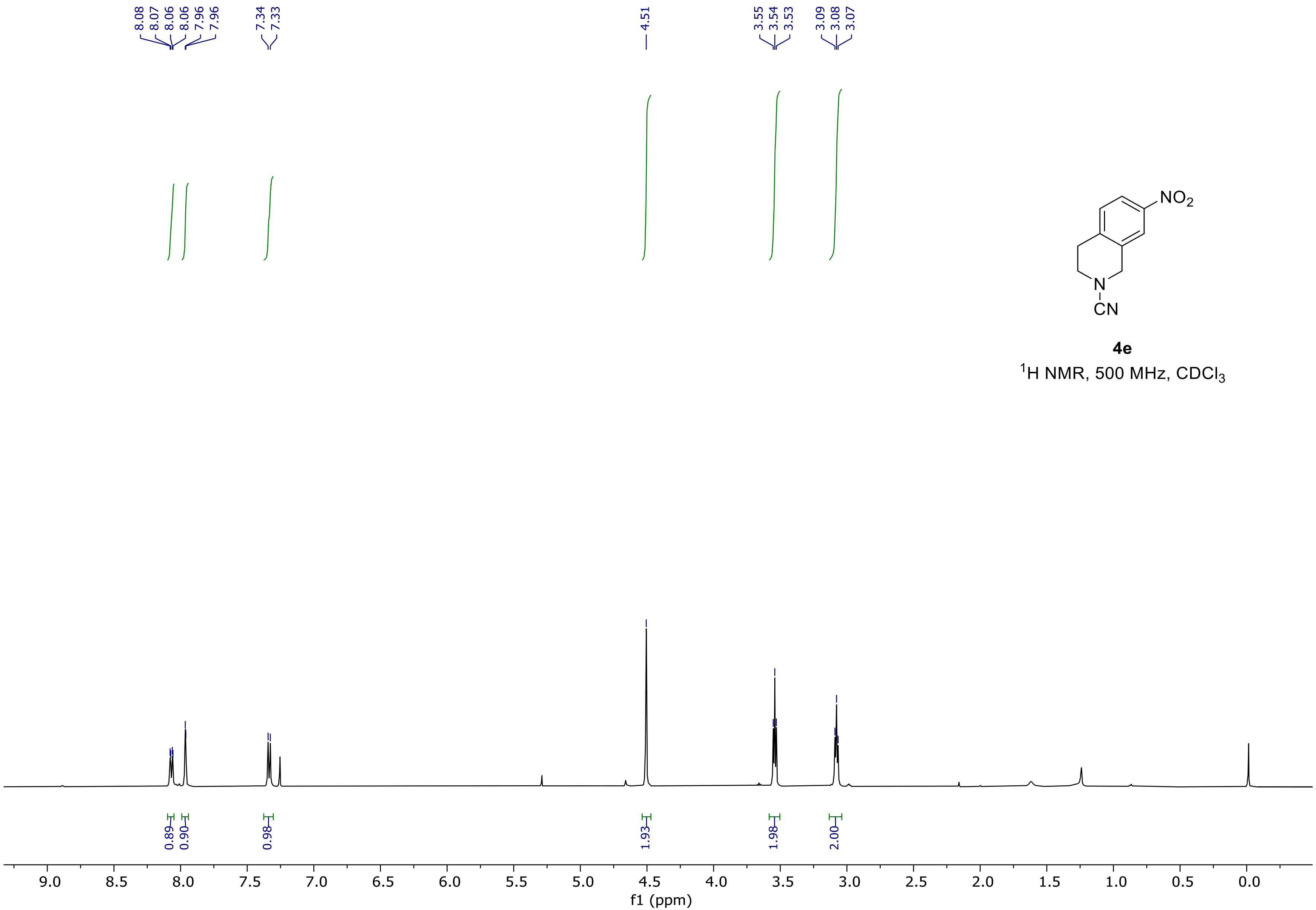

4e

^{1}H NMR, 500 MHz, $CDCl_3$

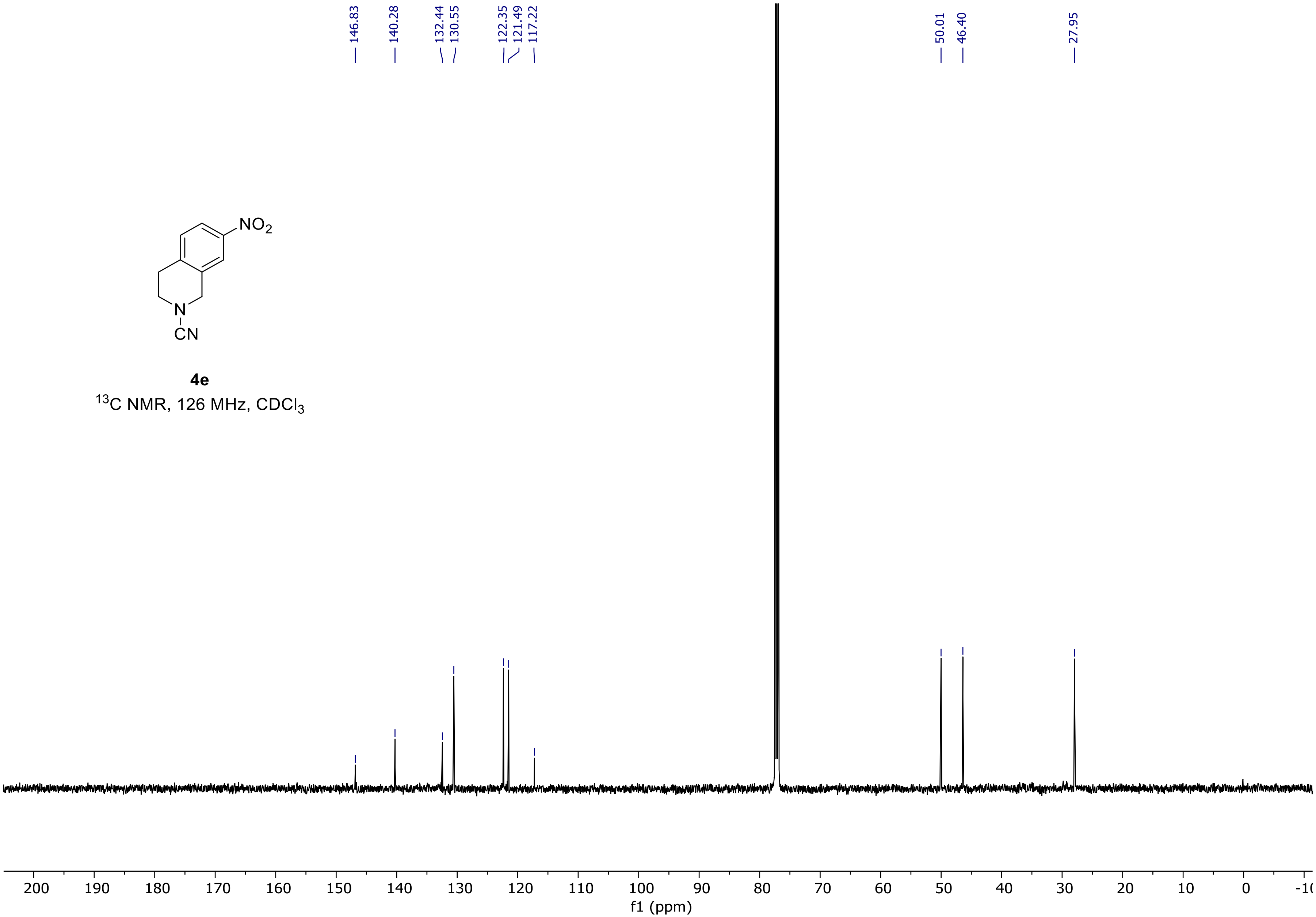

4e

^{13}C NMR, 126 MHz, $CDCl_3$

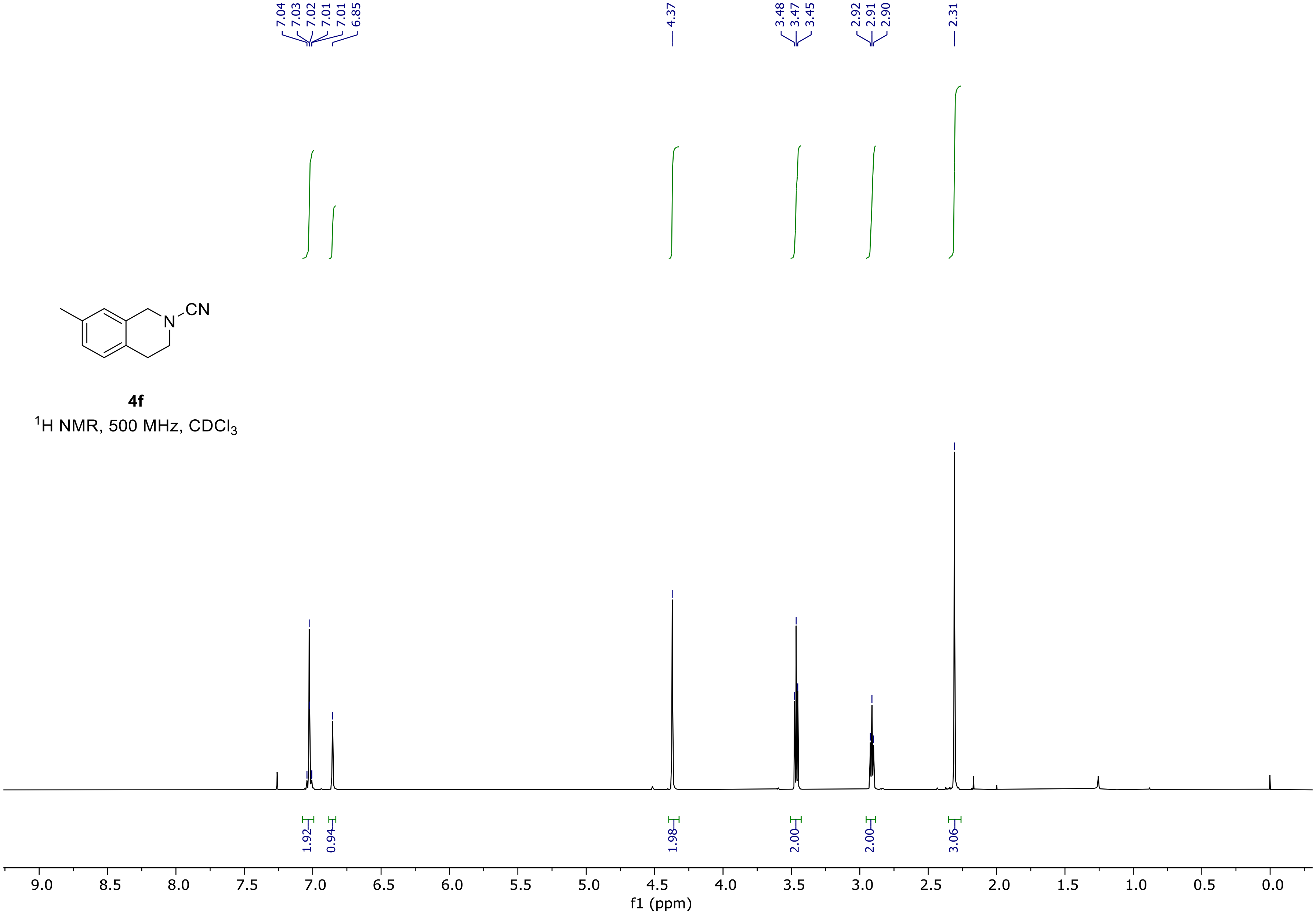

4f

^{1}H NMR, 500 MHz, $CDCl_3$

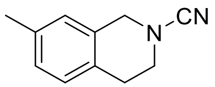

4f

^{13}C NMR, 126 MHz, $CDCl_3$

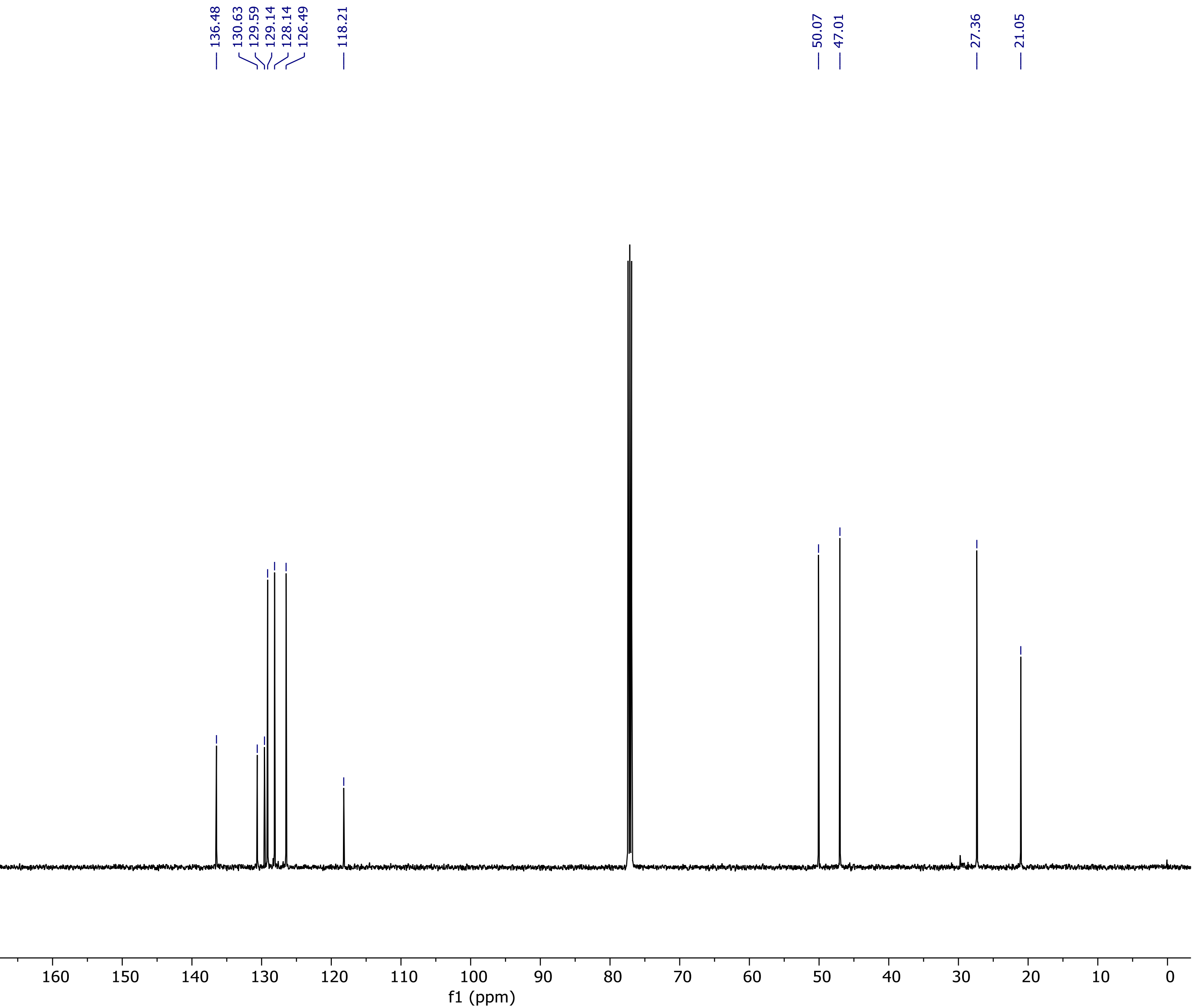

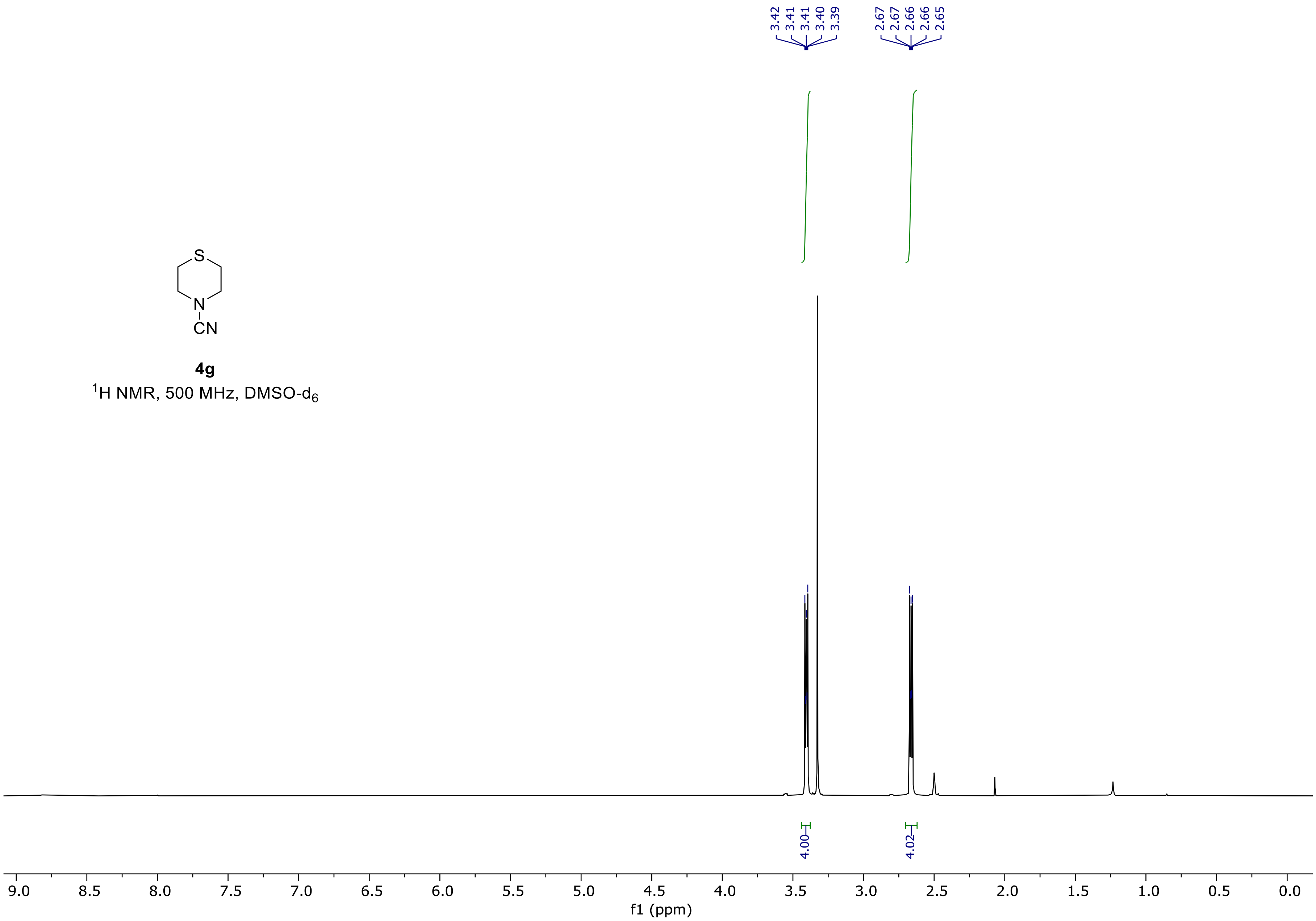

4g

^{1}H NMR, 500 MHz, DMSO-d_6

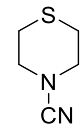

4g

^{13}C NMR, 126 MHz, DMSO-d$_6$

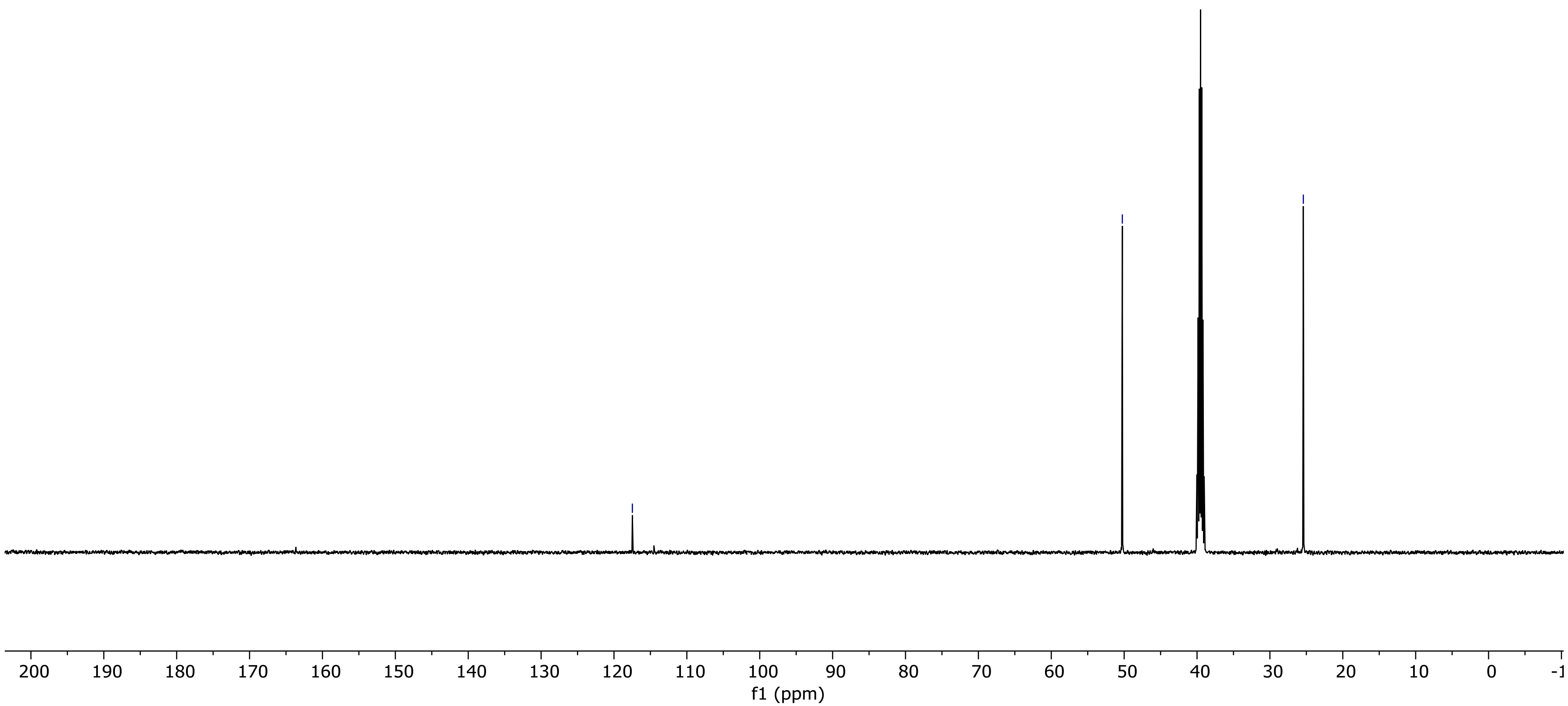

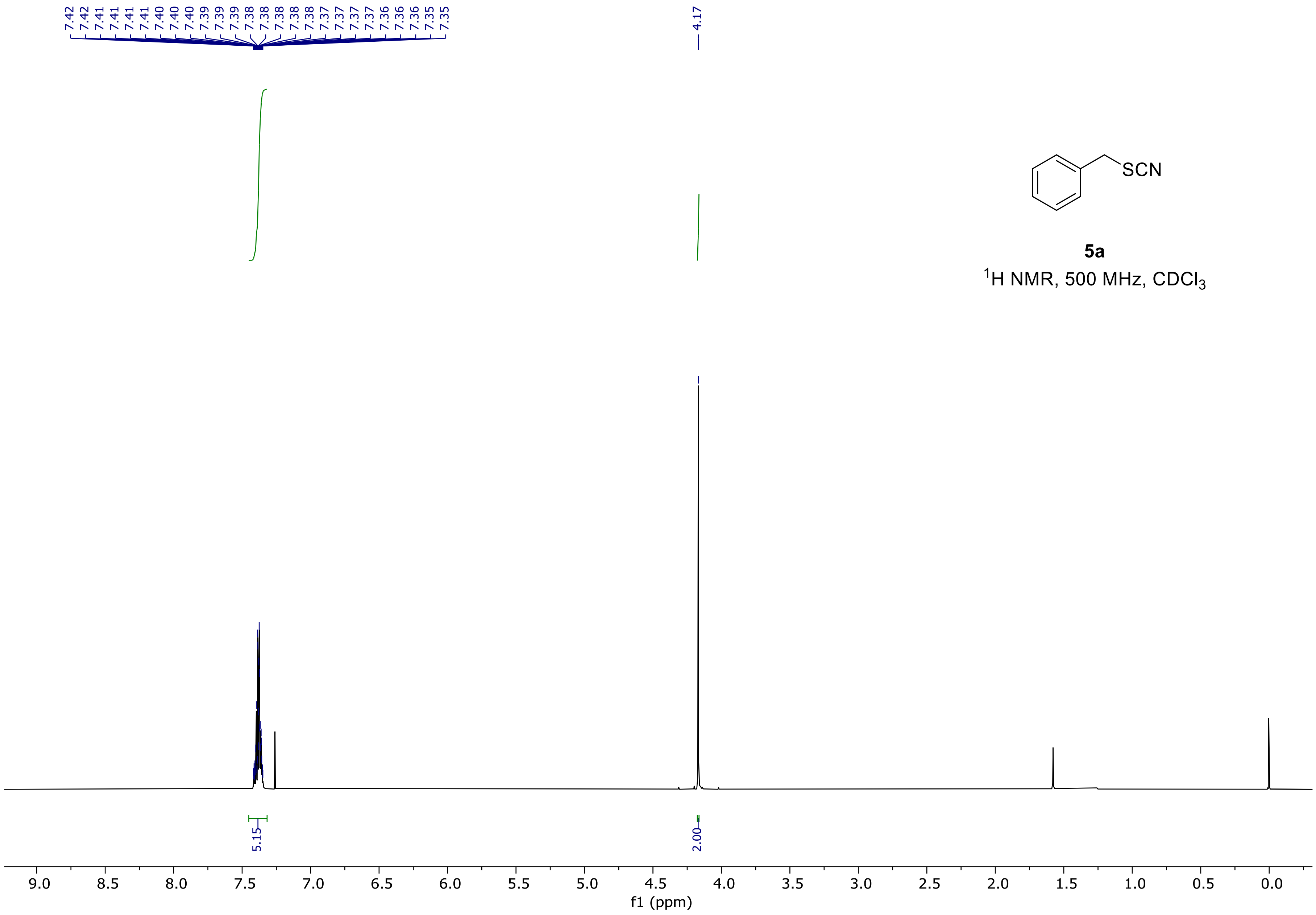

5a

^1H NMR, 500 MHz, $CDCl_3$

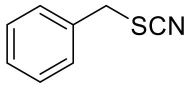

5a

^{13}C NMR, 126 MHz, $CDCl_3$

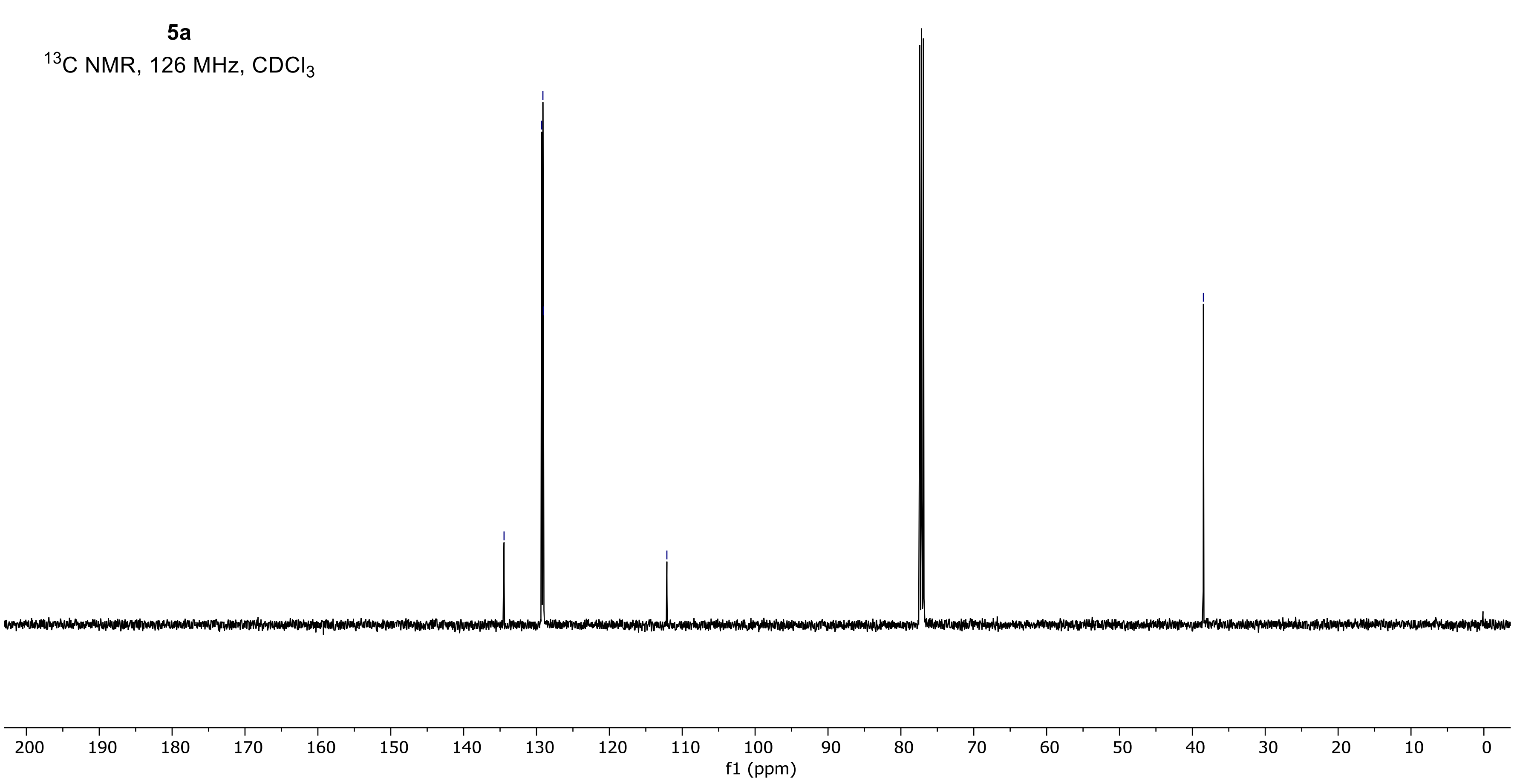

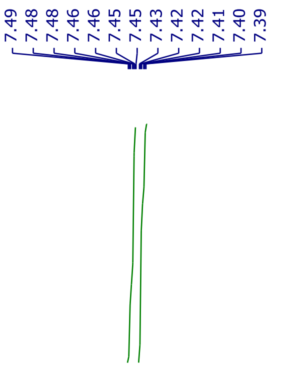

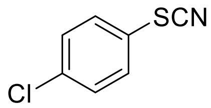

5b

^{1}H NMR, 400 MHz, $CDCl_3$

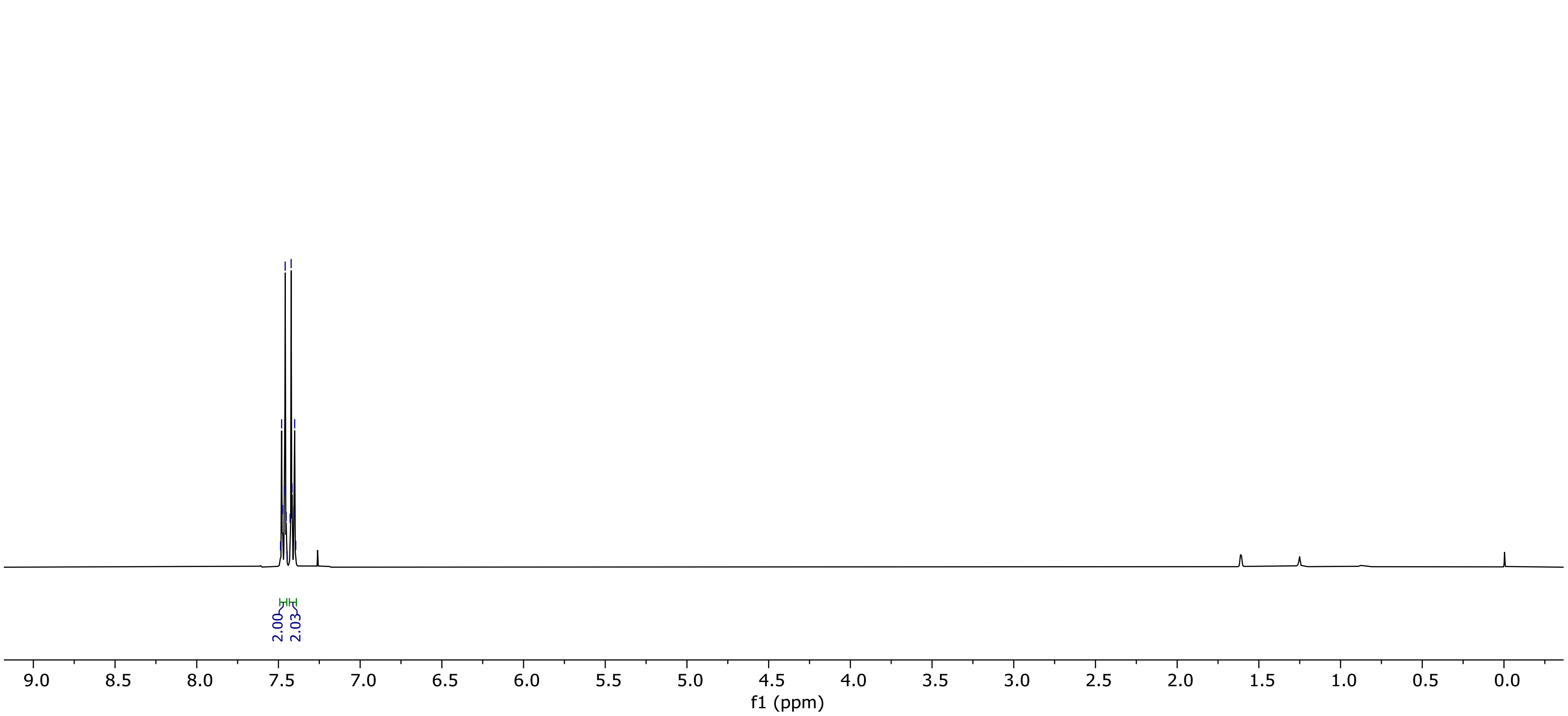

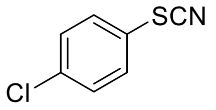

5b

^{13}C NMR, 101 MHz, $CDCl_3$

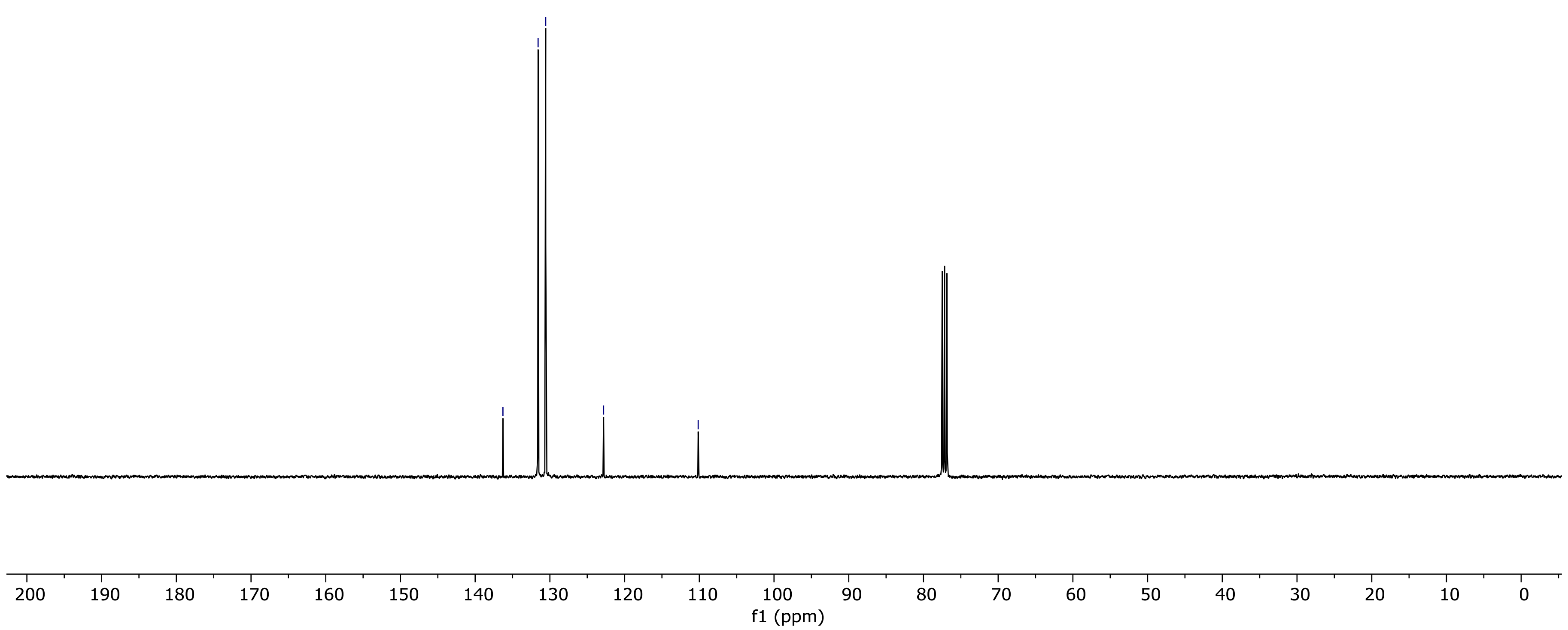

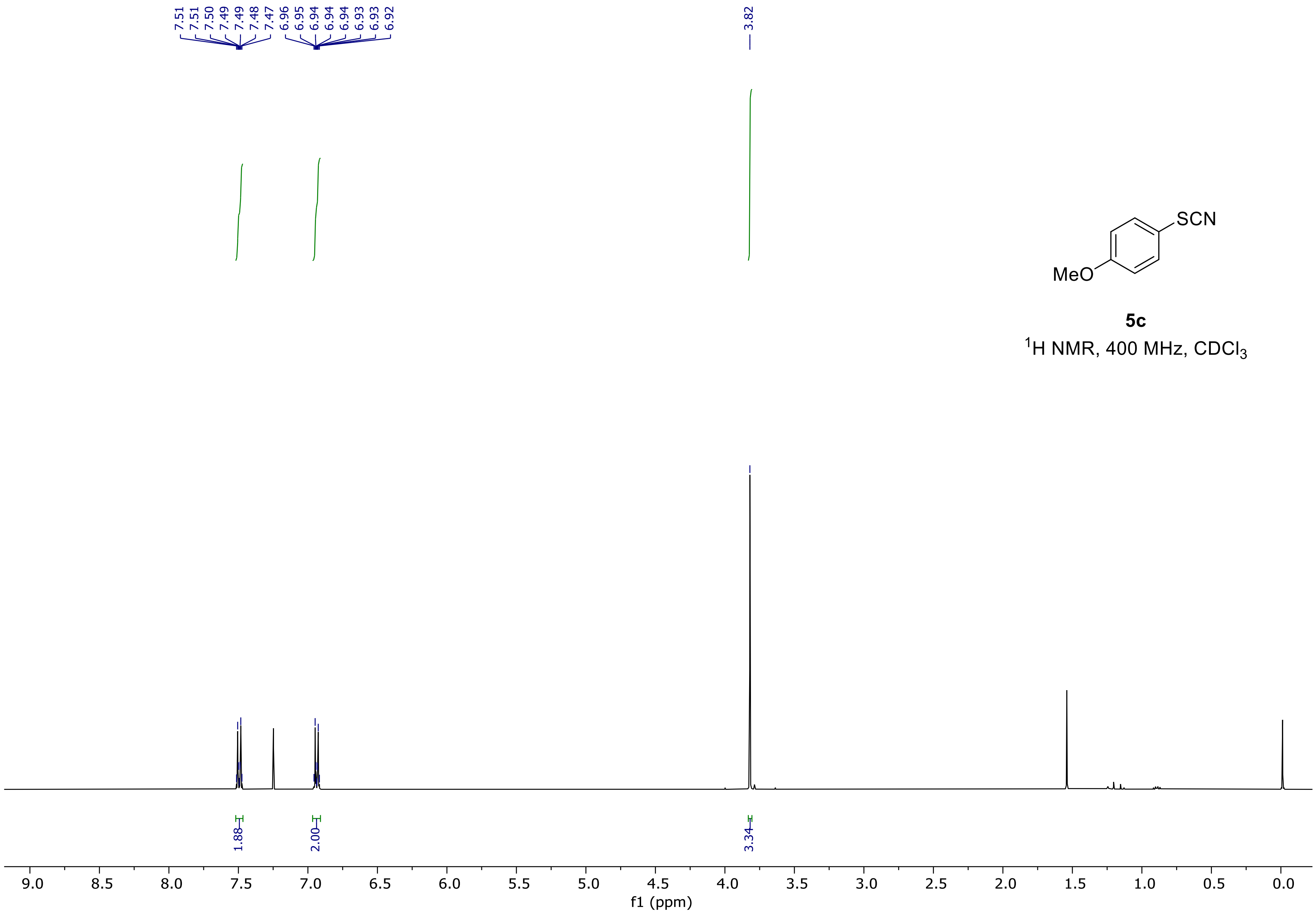

5c

^{1}H NMR, 400 MHz, $CDCl_3$

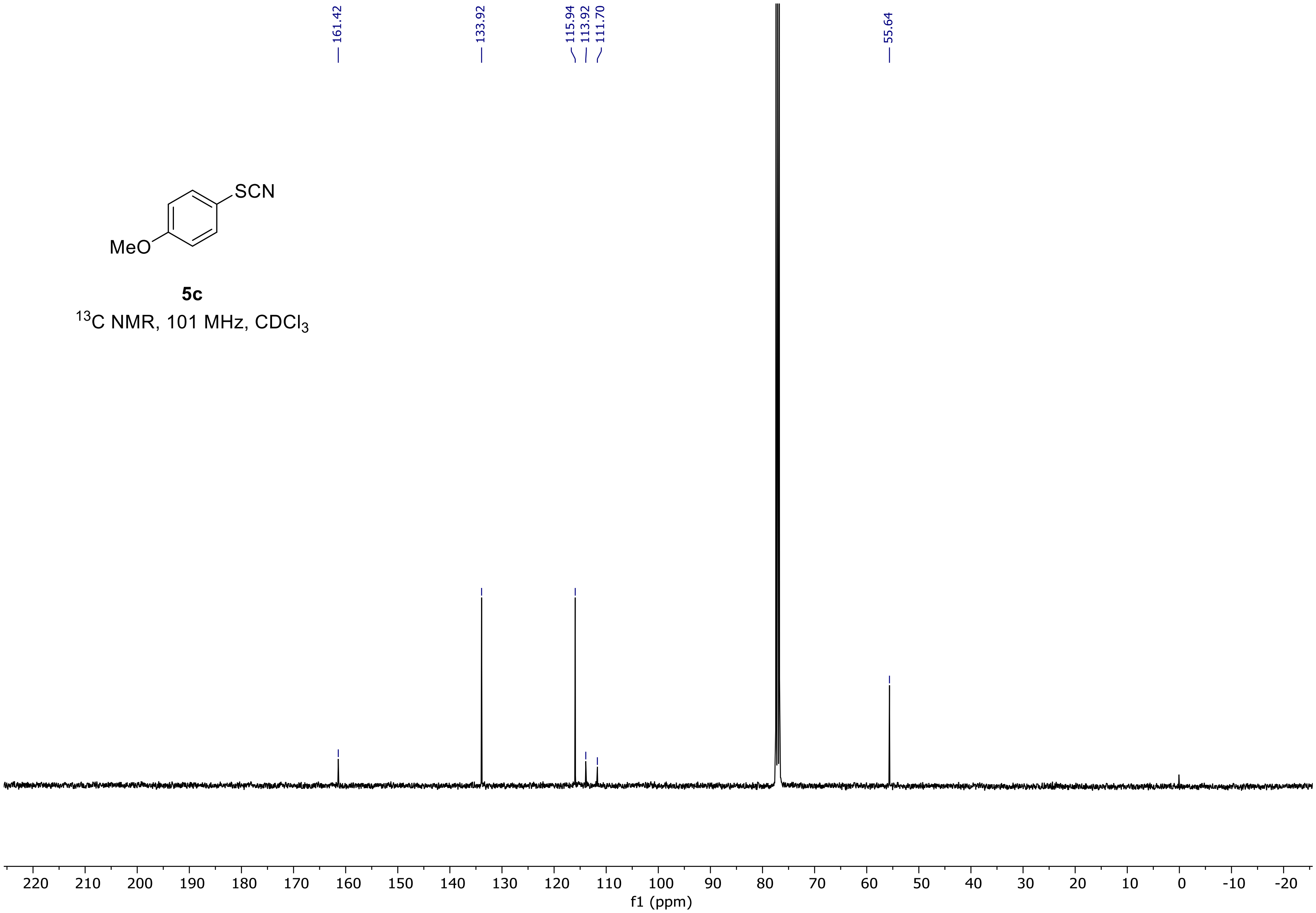

5c

^{13}C NMR, 101 MHz, $CDCl_3$

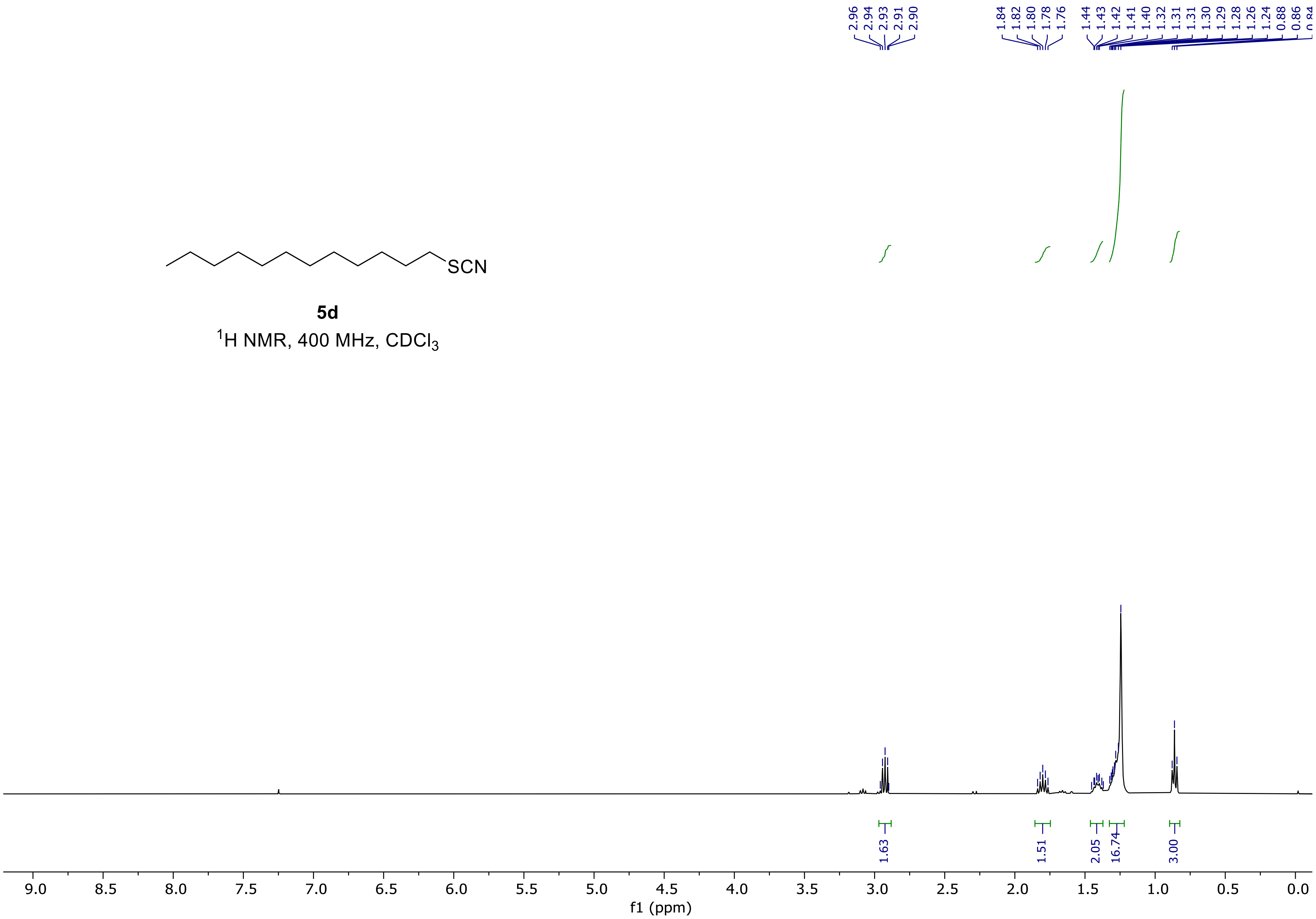

5d

^{1}H NMR, 400 MHz, $CDCl_3$

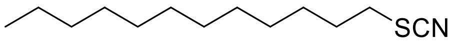

5d

^{13}C NMR, 101 MHz, $CDCl_3$

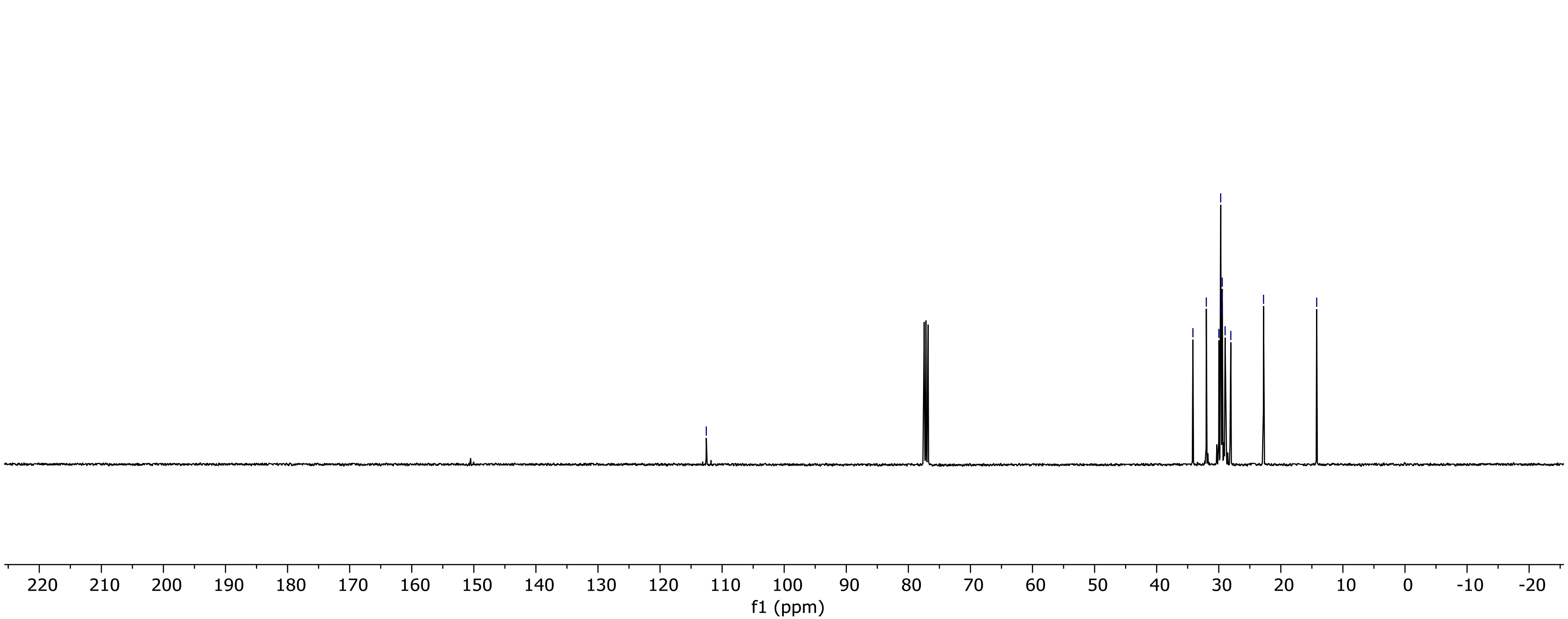

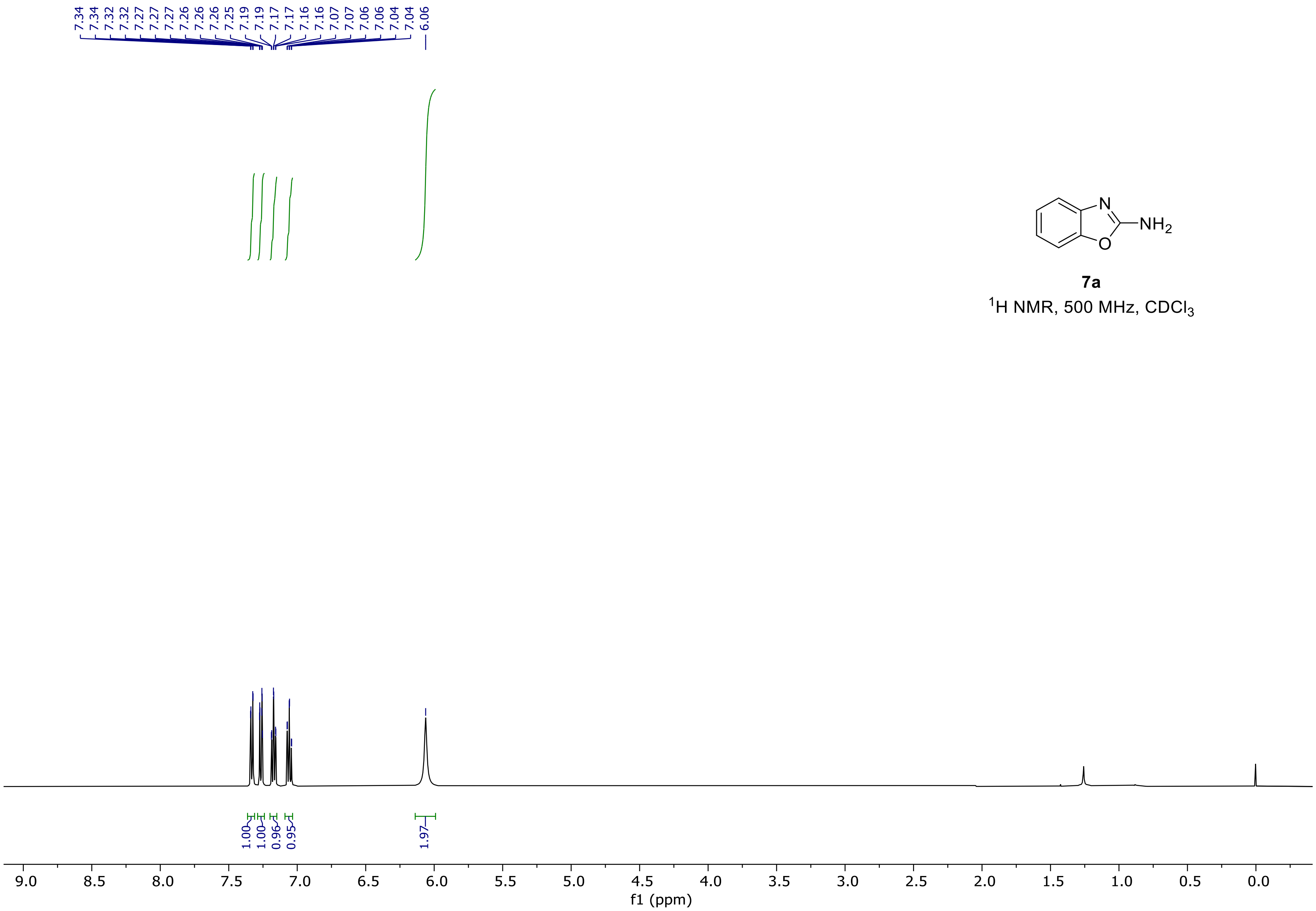

7a

^{1}H NMR, 500 MHz, $CDCl_3$

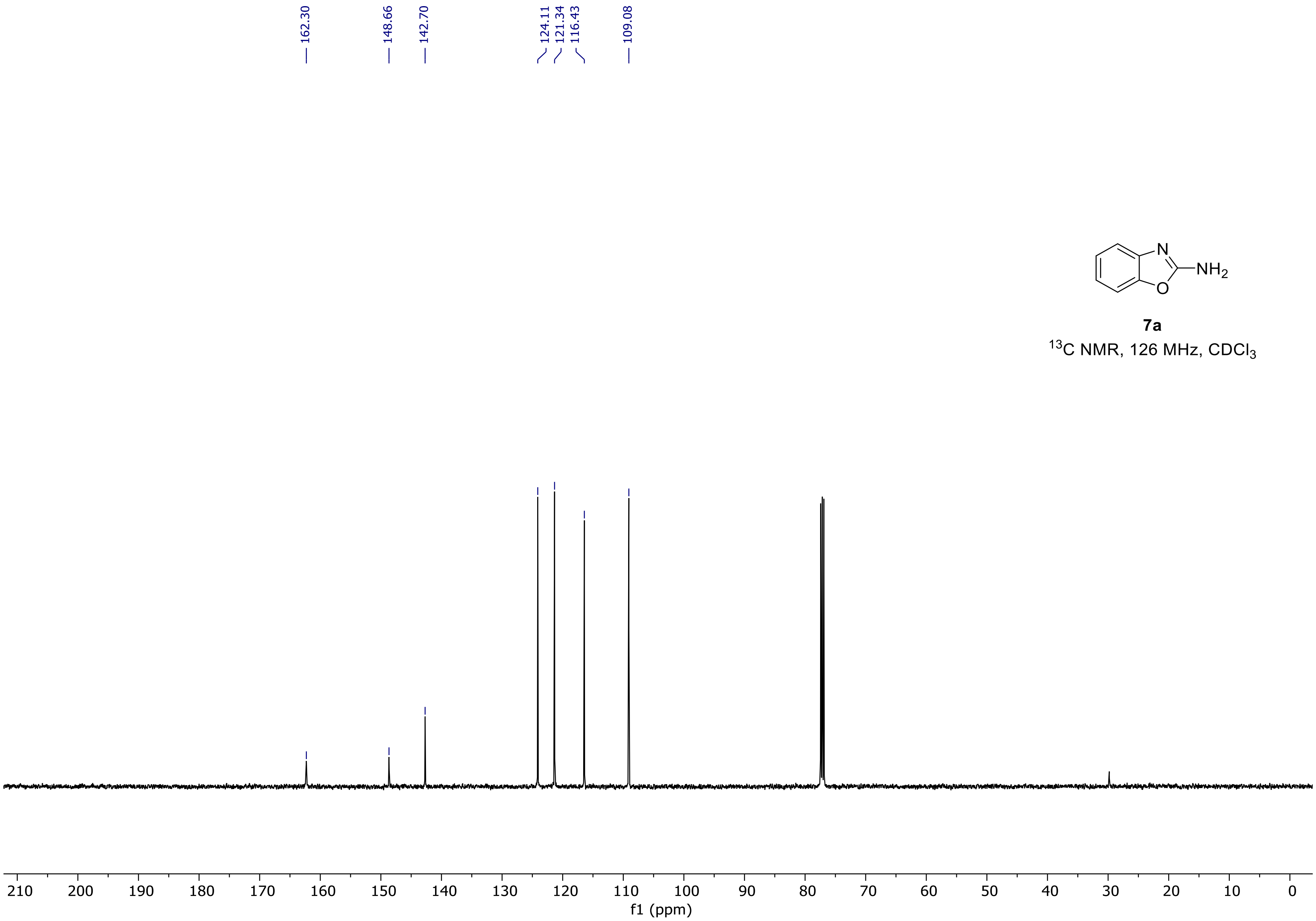

7a

^{13}C NMR, 126 MHz, $CDCl_3$

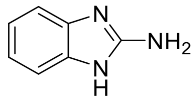

7b

^{1}H NMR, 500 MHz, DMSO-d_6

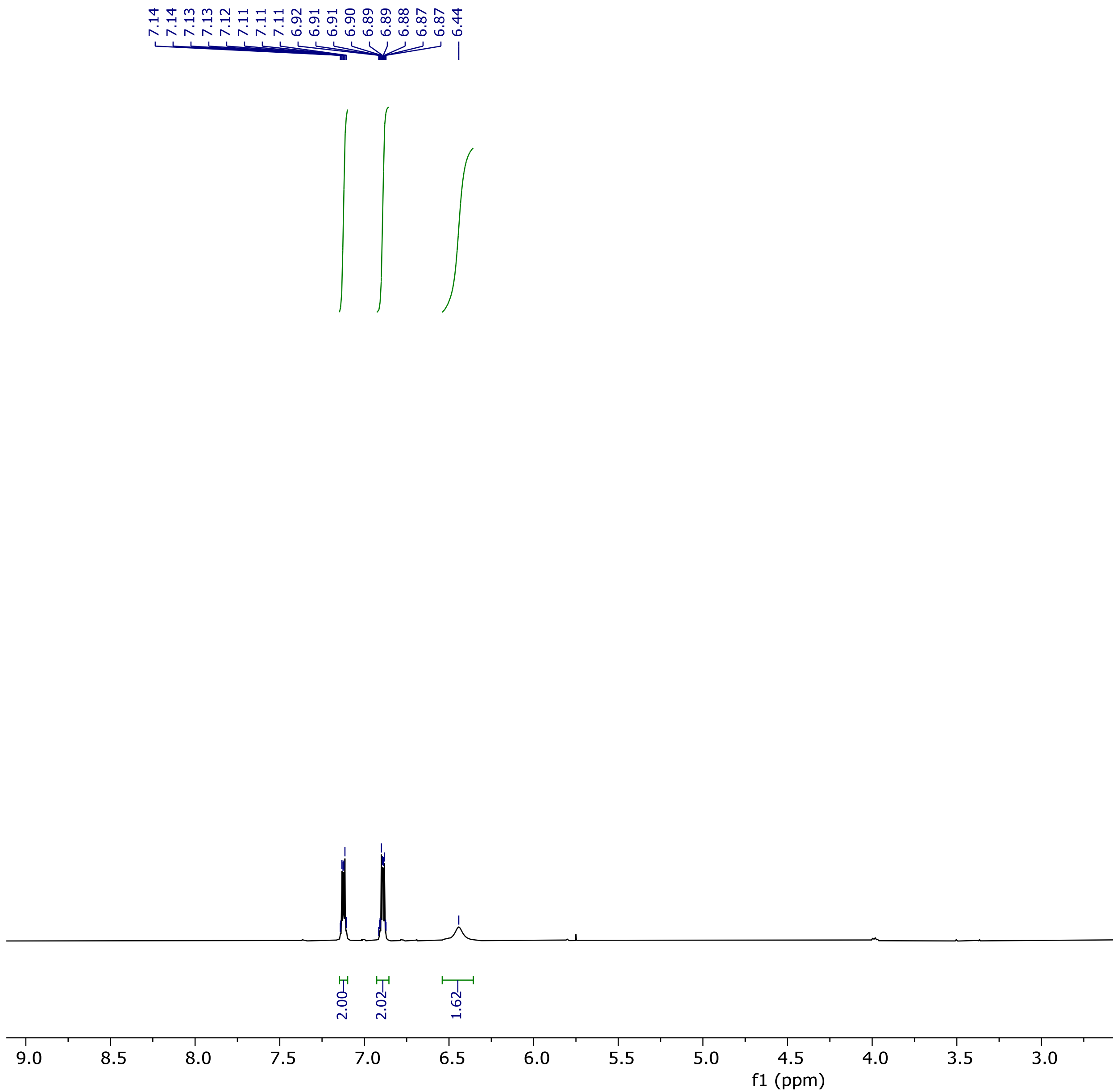

7b

^{13}C NMR, 126 MHz, DMSO-d_6

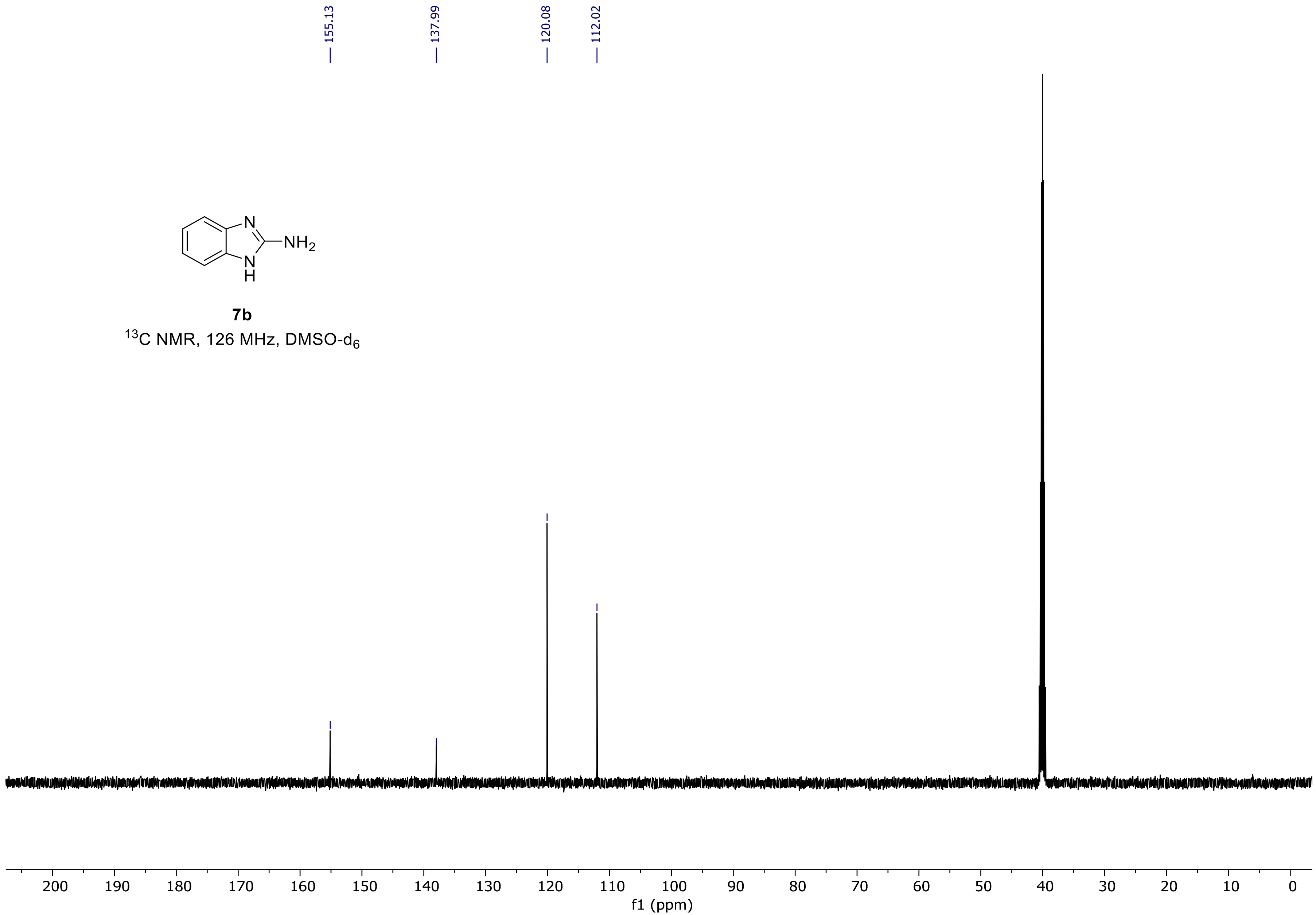

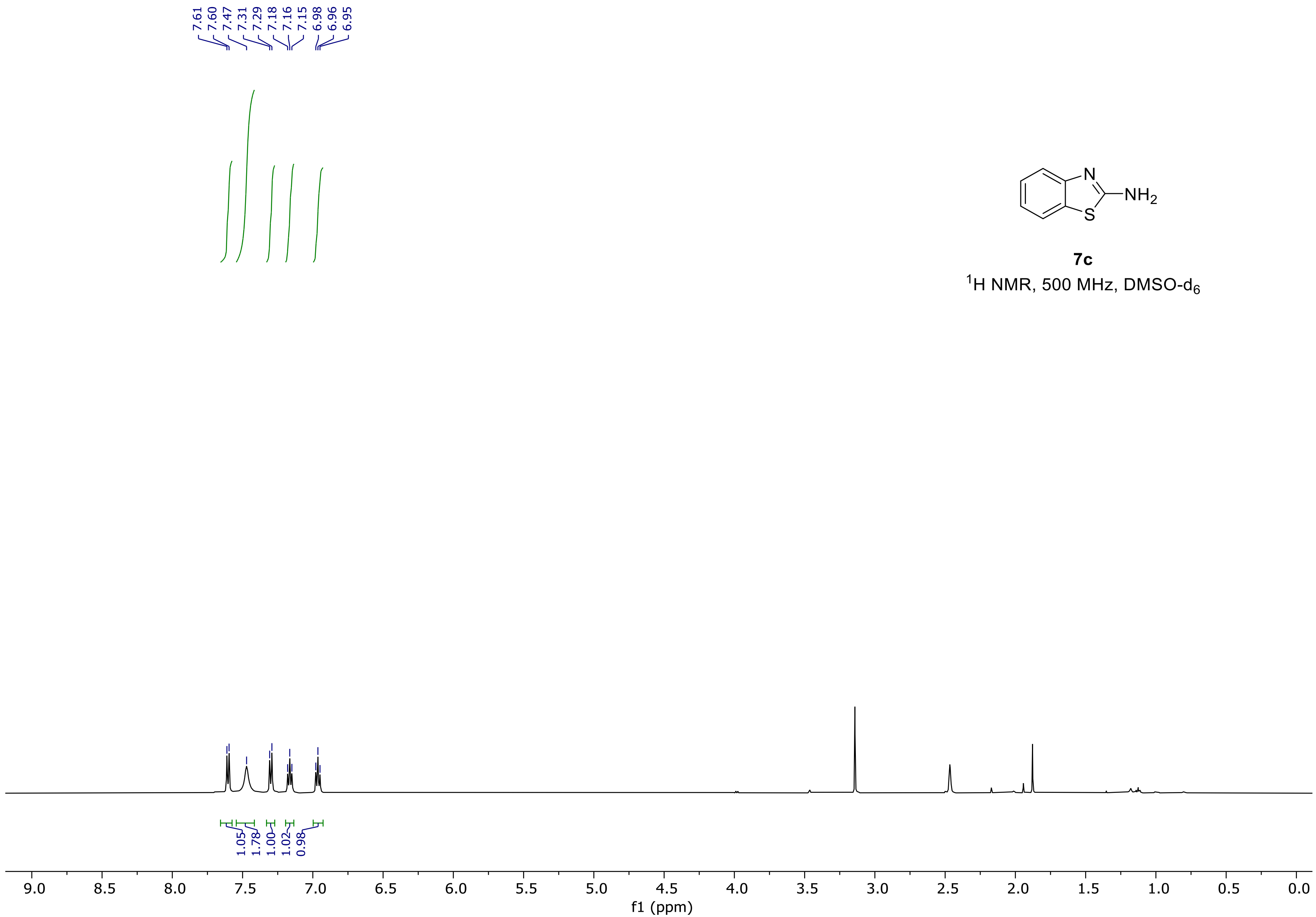

7c

^{1}H NMR, 500 MHz, DMSO-d_6

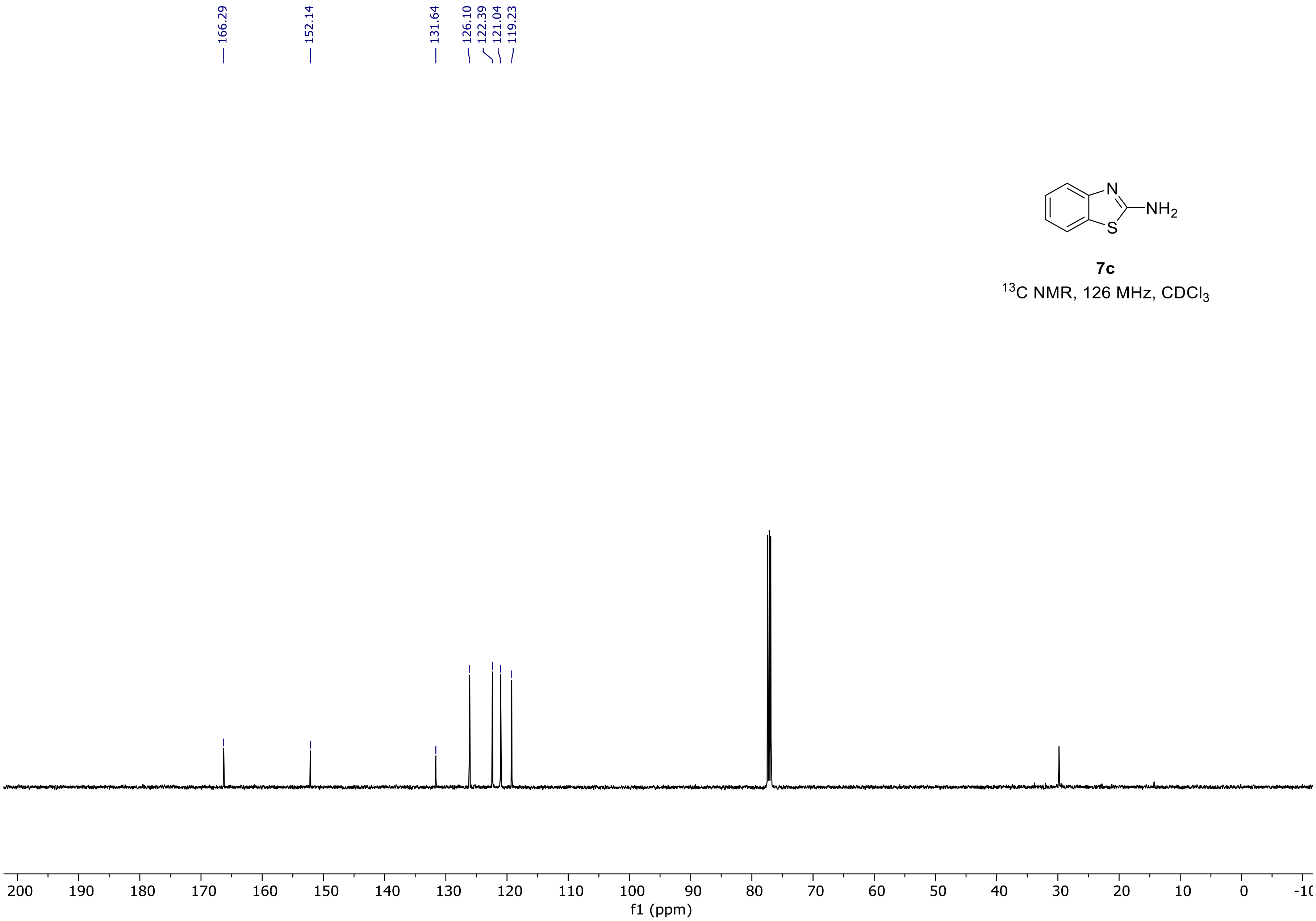
N
NH2
S
7c
13C NMR, 126 MHz, CDCl3
166.29
152.14
131.64
126.10
122.39
121.04
119.23
200 190 180 170 160 150 140 130 120 110 100 90 80 70 60 50 40 30 20 10 0
f1 (ppm)

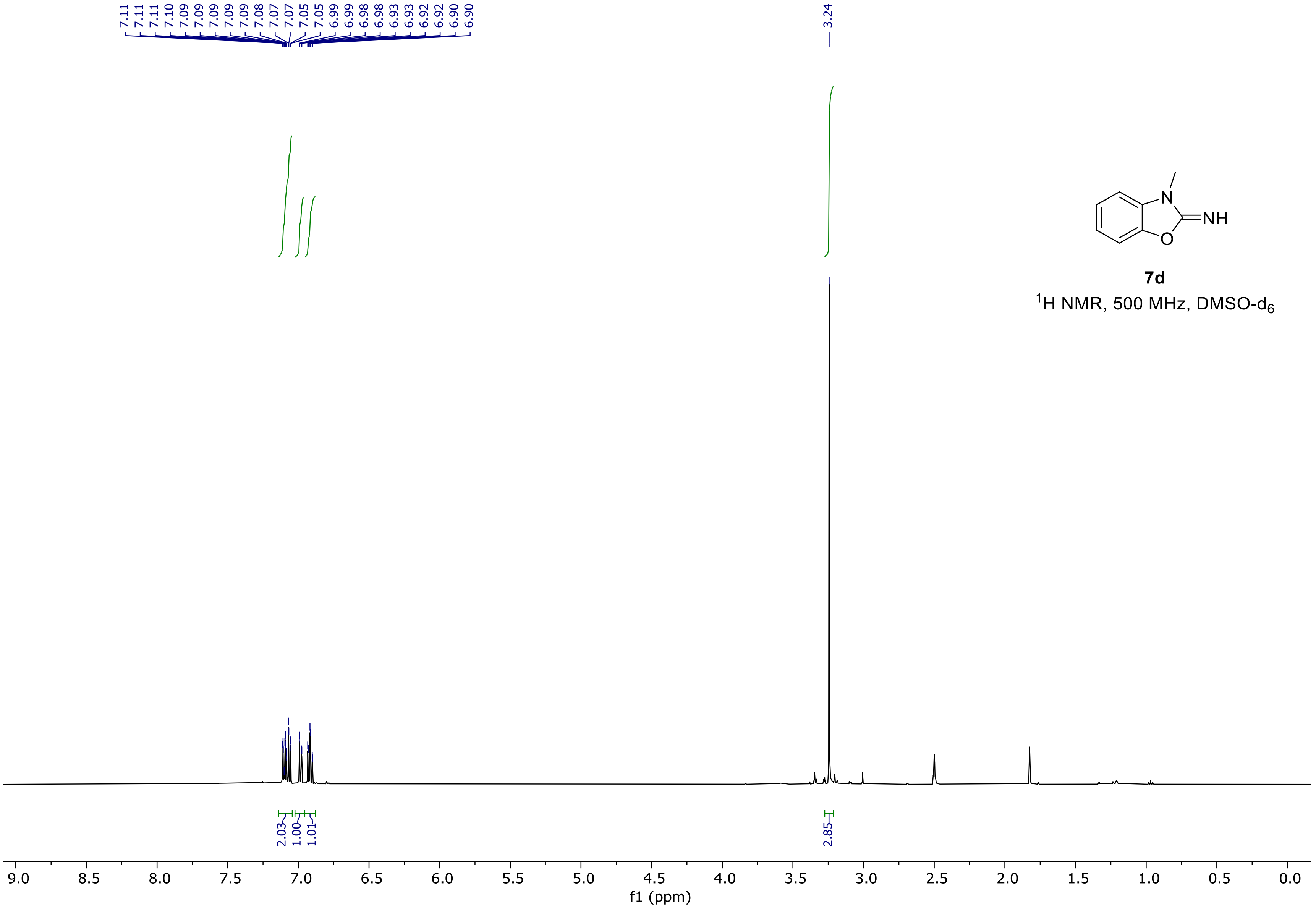

7d

^{1}H NMR, 500 MHz, DMSO-d_6

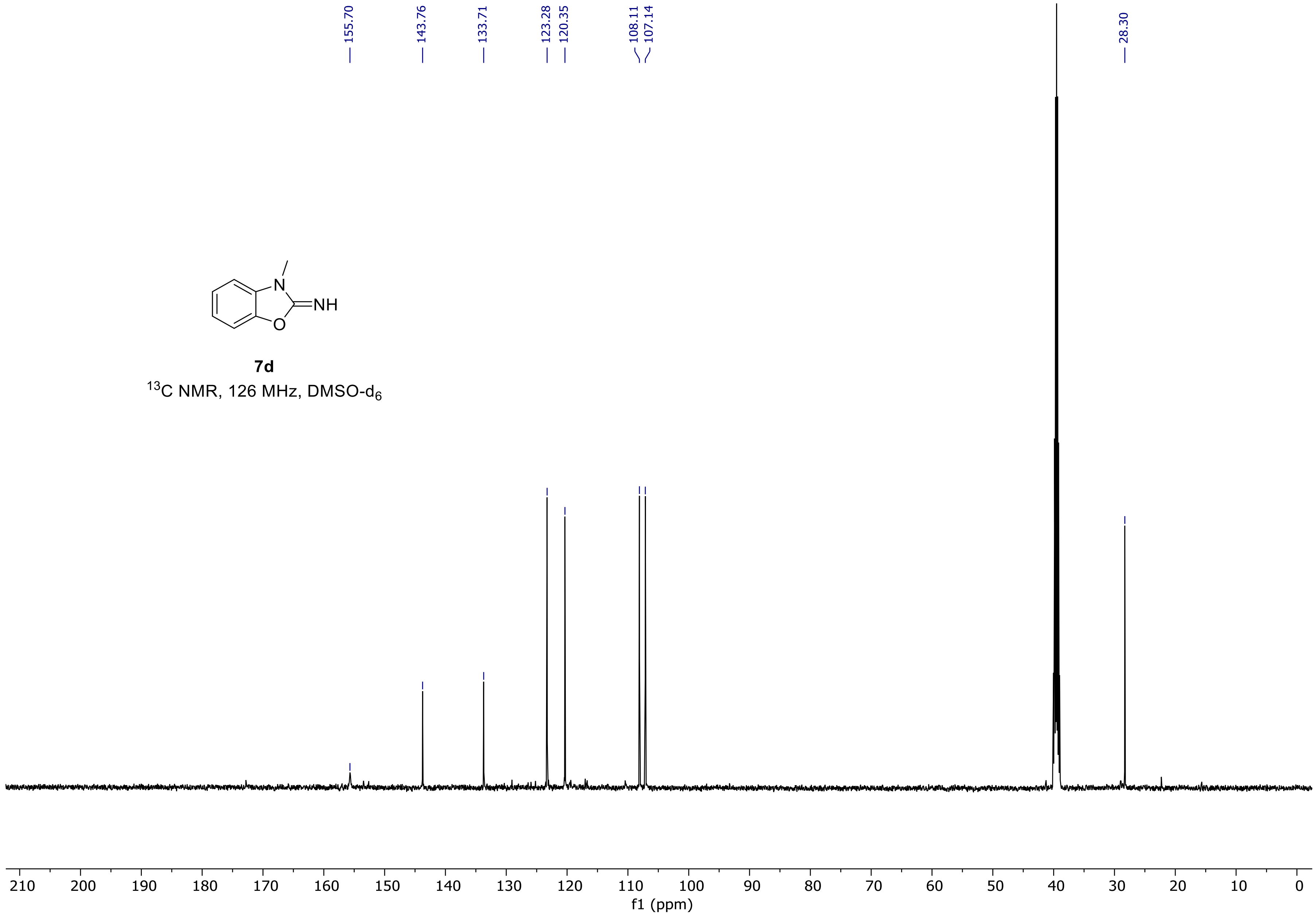

7d

^{13}C NMR, 126 MHz, DMSO-d_6

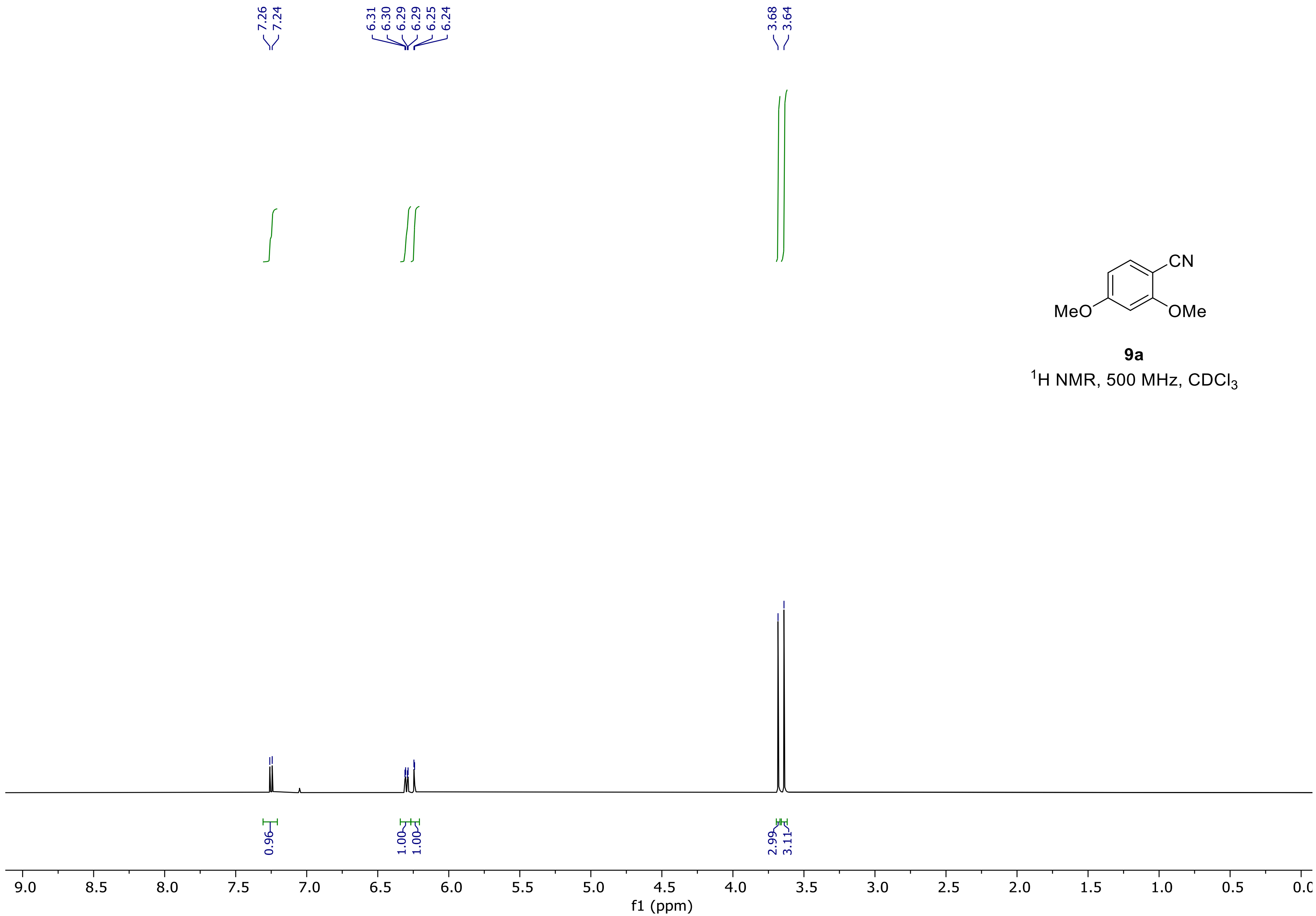

9a

^{1}H NMR, 500 MHz, $CDCl_3$

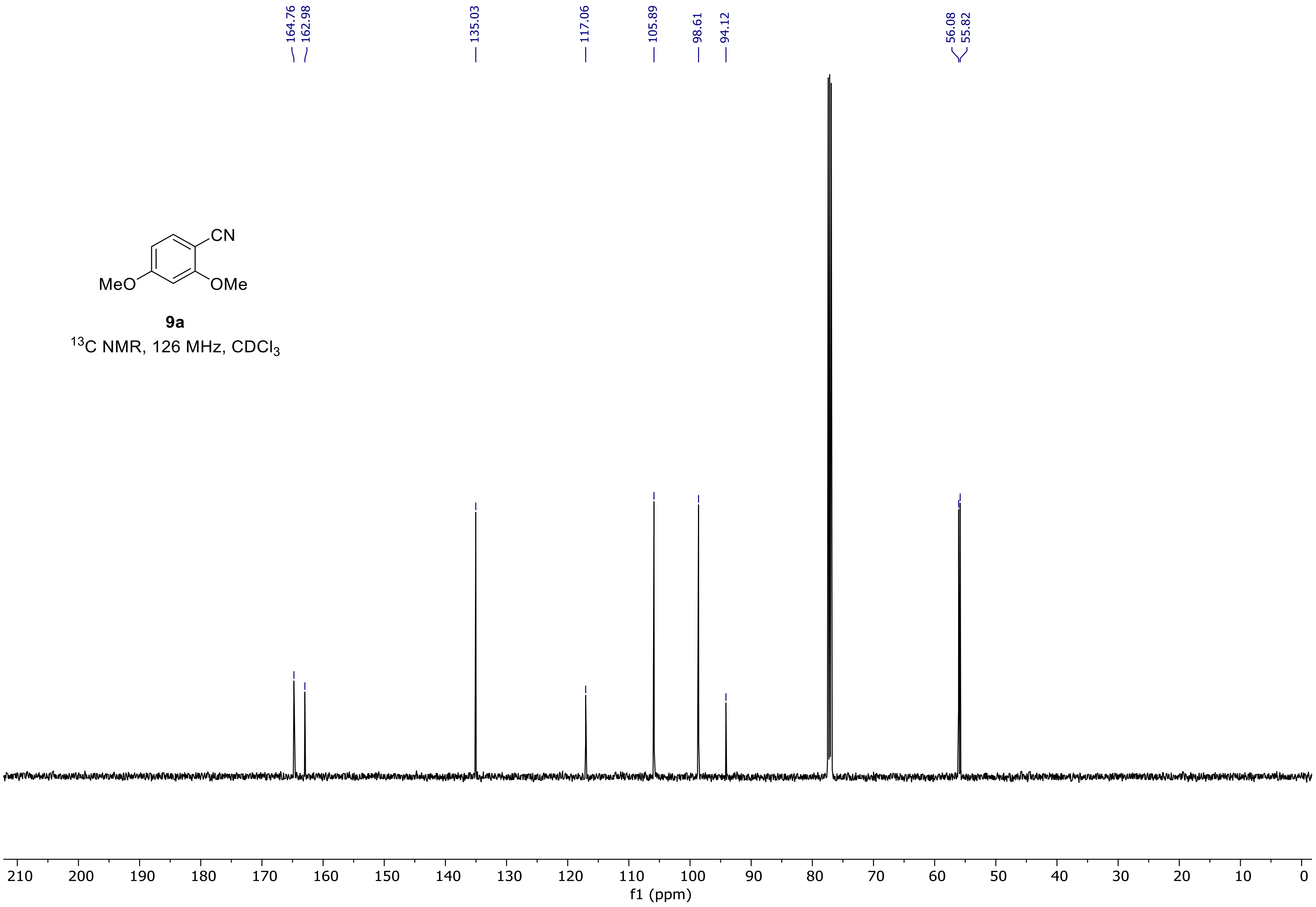
CN
MeO
OMe
9a
13C NMR, 126 MHz, CDCl3
164.76
162.98
135.03
117.06
105.89
98.61
94.12
56.08
55.82
210 200 190 180 170 160 150 140 130 120 110 100 90 80 70 60 50 40 30 20 10 0
f1 (ppm)

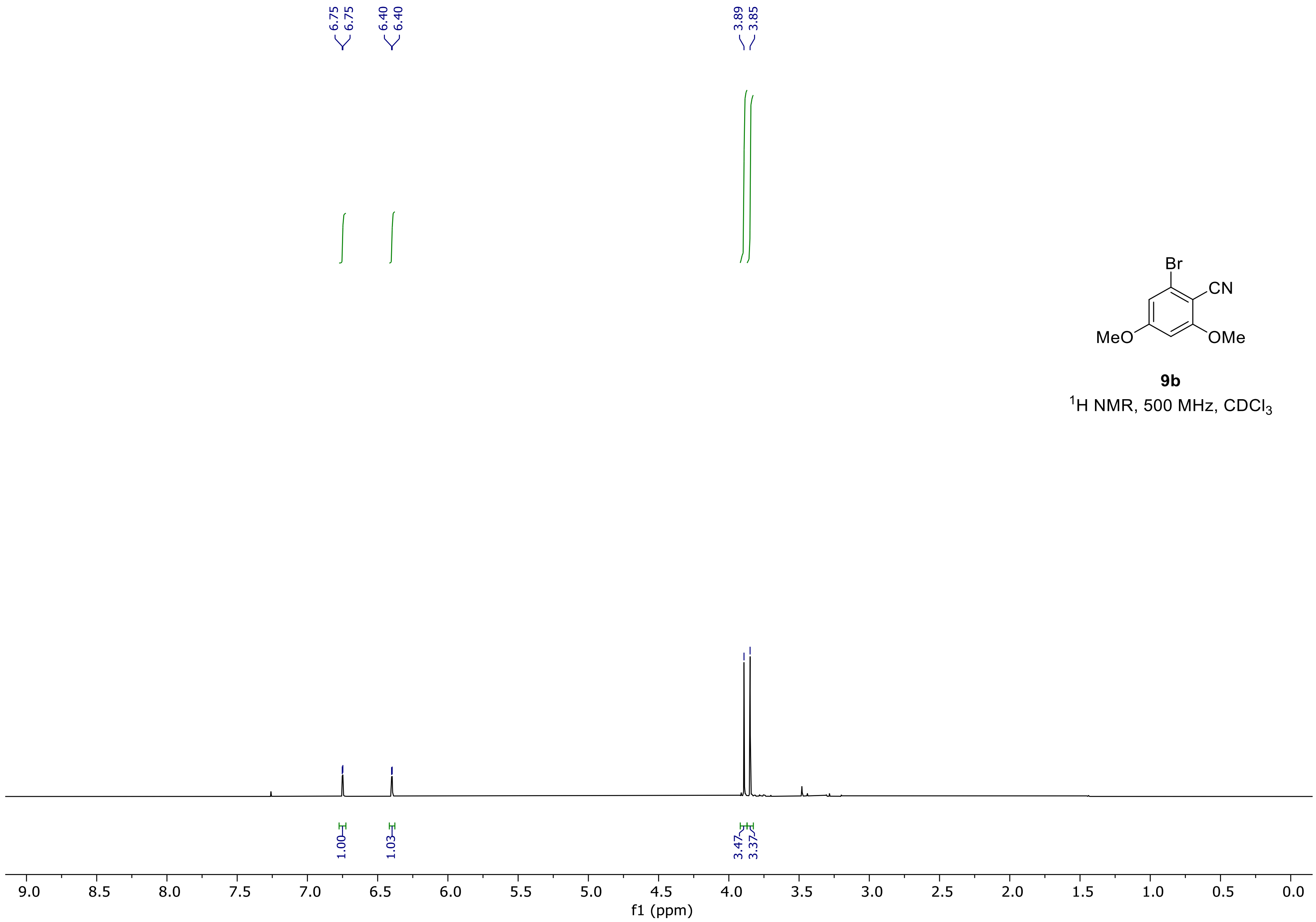

9b

^{1}H NMR, 500 MHz, $CDCl_3$

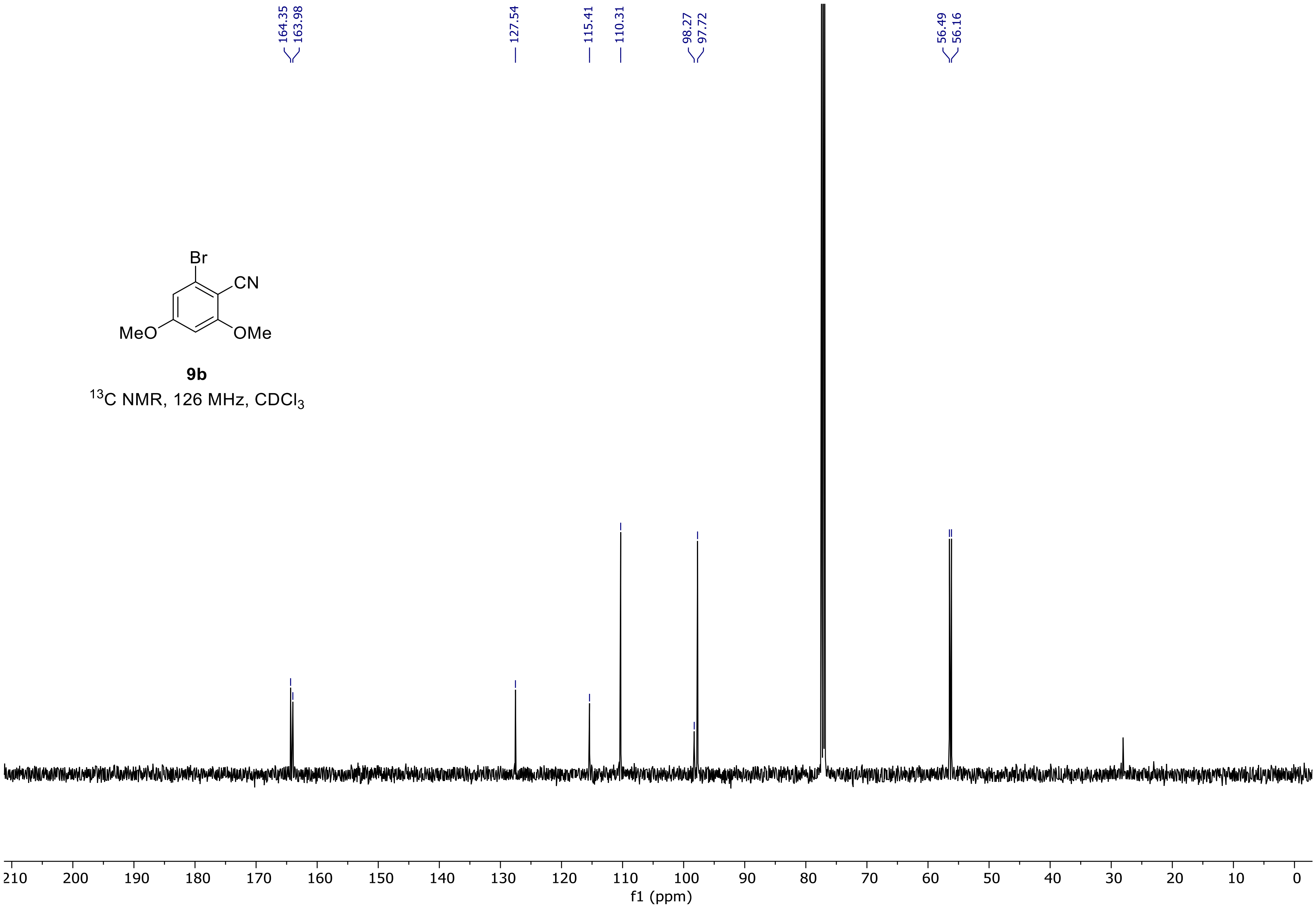

9b

^{13}C NMR, 126 MHz, $CDCl_3$

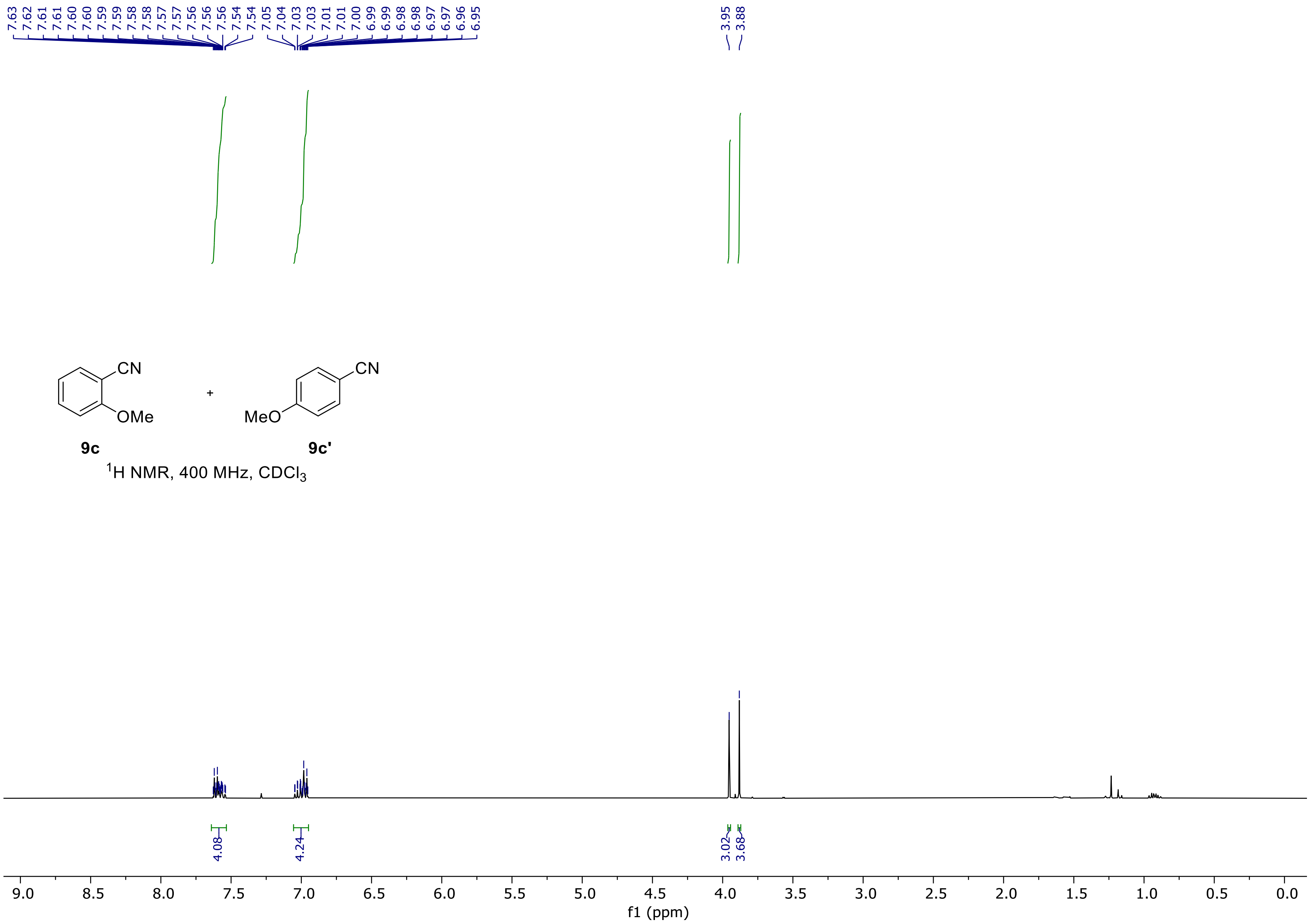

^{1}H NMR, 400 MHz, $CDCl_3$

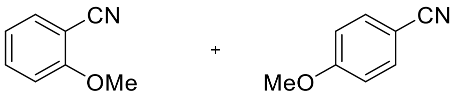

9c **9c'**

^{13}C NMR, 101 MHz, $CDCl_3$

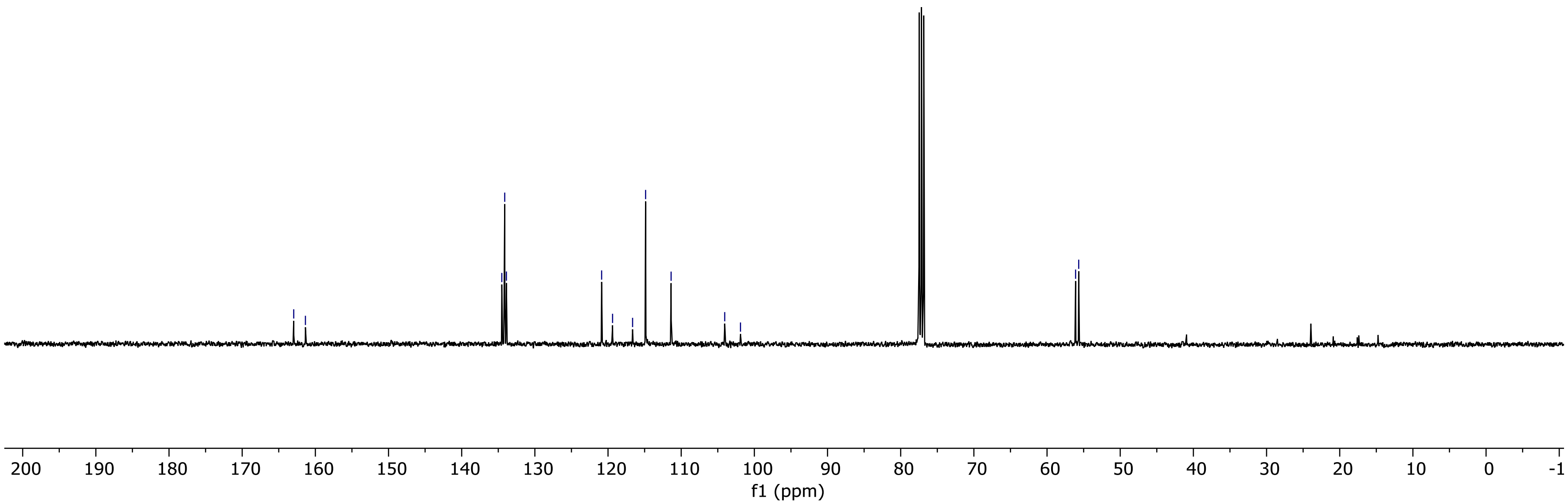

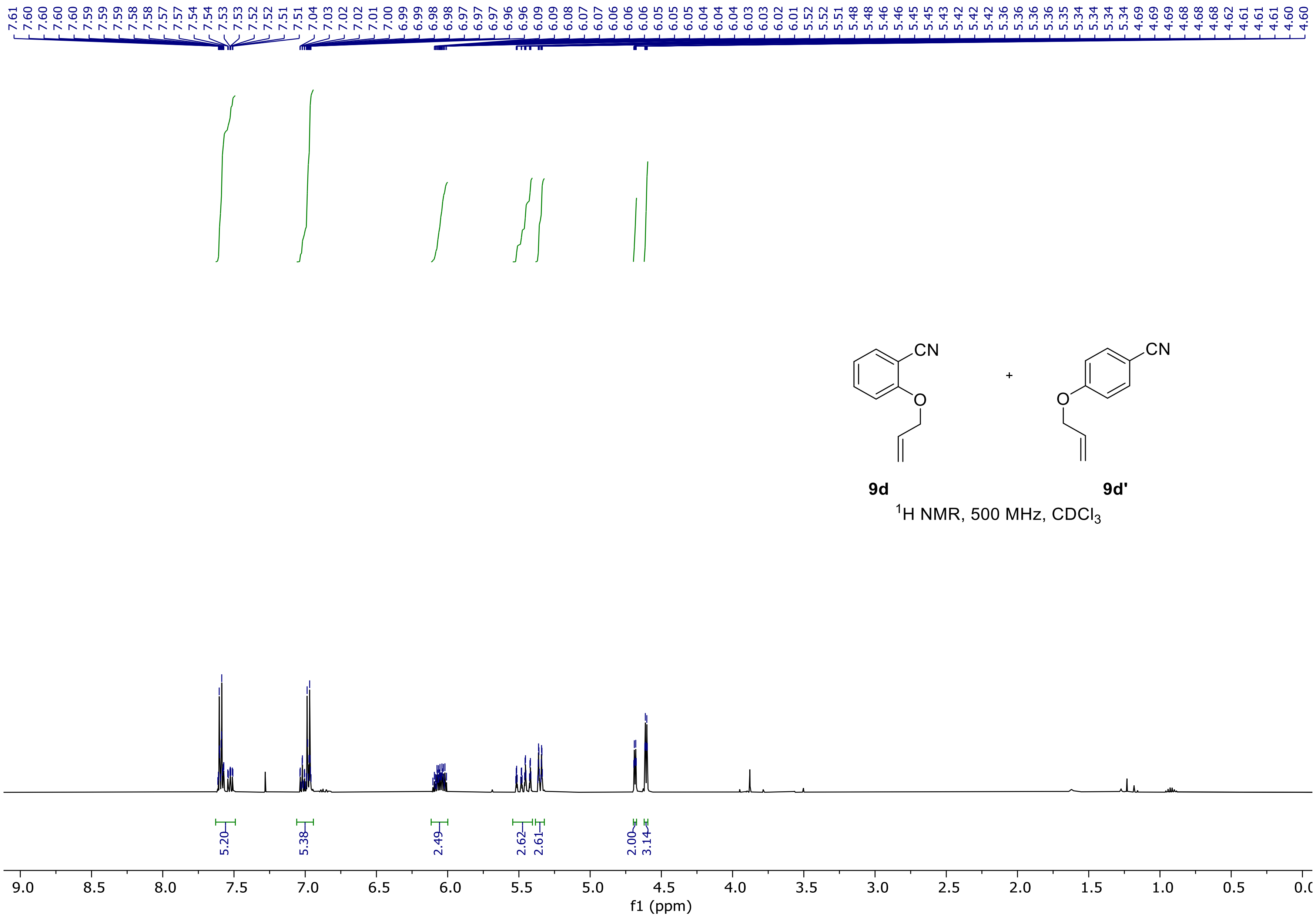
7.61 7.60 7.60 7.60 7.59 7.59 7.59 7.58 7.58 7.57 7.57 7.54 7.54 7.53 7.53 7.52 7.52 7.51 7.51 7.04 7.03 7.02 7.02 7.01 7.00 6.99 6.99 6.98 6.98 6.97 6.97 6.96 6.96 6.09 6.09 6.08 6.07 6.07 6.06 6.06 6.06 6.05 6.05 6.04 6.04 6.04 6.03 6.03 6.02 6.01 5.52 5.52 5.51 5.48 5.48 5.46 5.46 5.45 5.45 5.43 5.42 5.42 5.42 5.36 5.36 5.36 5.35 5.34 5.34 5.34 4.69 4.69 4.69 4.68 4.68 4.68 4.62 4.61 4.61 4.61 4.60 4.60
CN
+
CN
O
O
9d
9d'
^{1}H NMR, 500 MHz, $CDCl_3$
5.20
5.38
2.49
2.62
2.61
2.00
3.14
9.0 8.5 8.0 7.5 7.0 6.5 6.0 5.5 5.0 4.5 4.0 3.5 3.0 2.5 2.0 1.5 1.0 0.5 0.0
f1 (ppm)

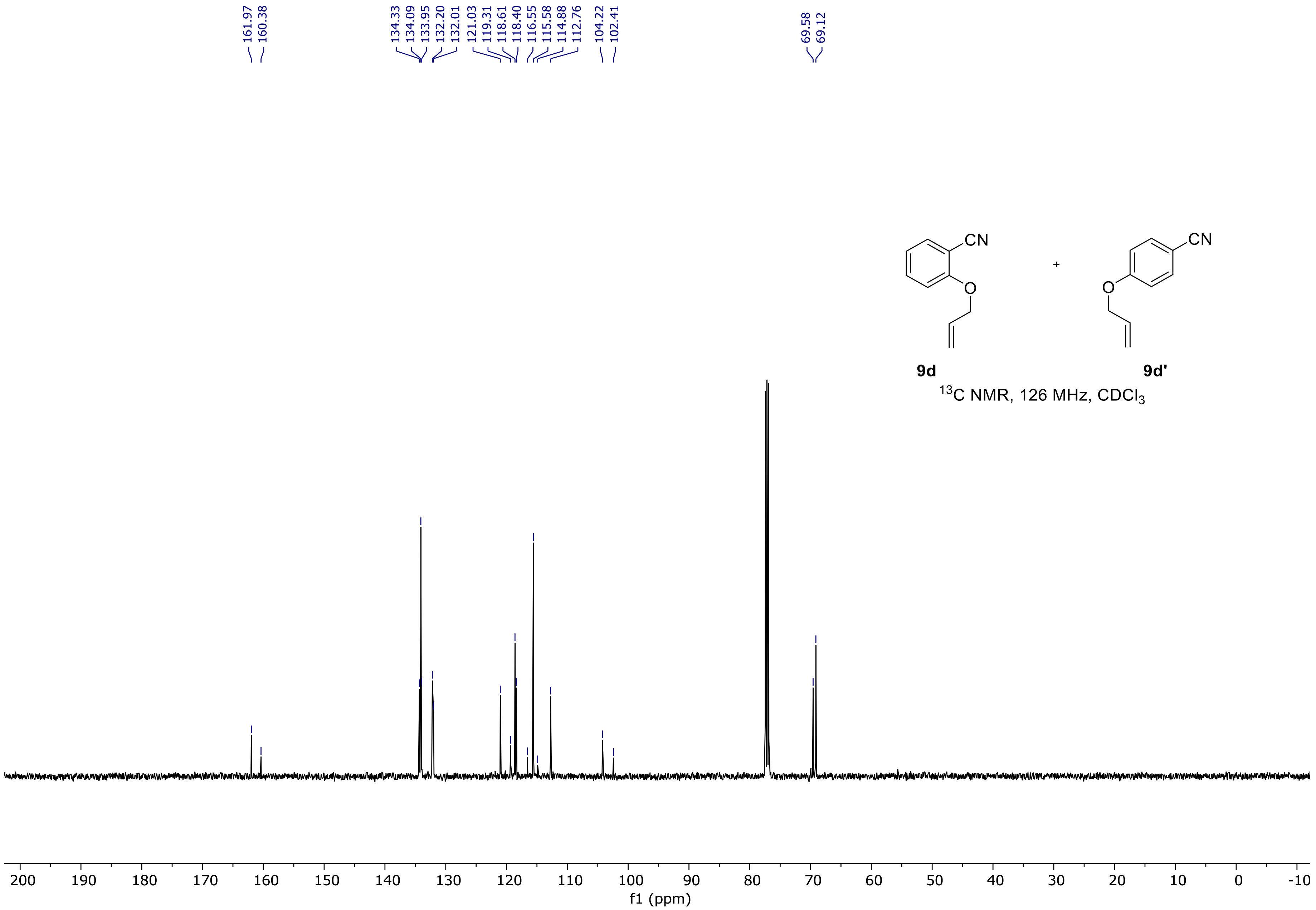
161.97
160.38
134.33
134.09
133.95
132.20
132.01
121.03
119.31
118.61
118.40
116.55
115.58
114.88
112.76
104.22
102.41
69.58
69.12
CN
O
9d
9d'
^{13}C NMR, 126 MHz, CDCl$_3$
200
190
180
170
160
150
140
130
120
110
100
90
80
70
60
50
40
30
20
10
0
-10
f1 (ppm)

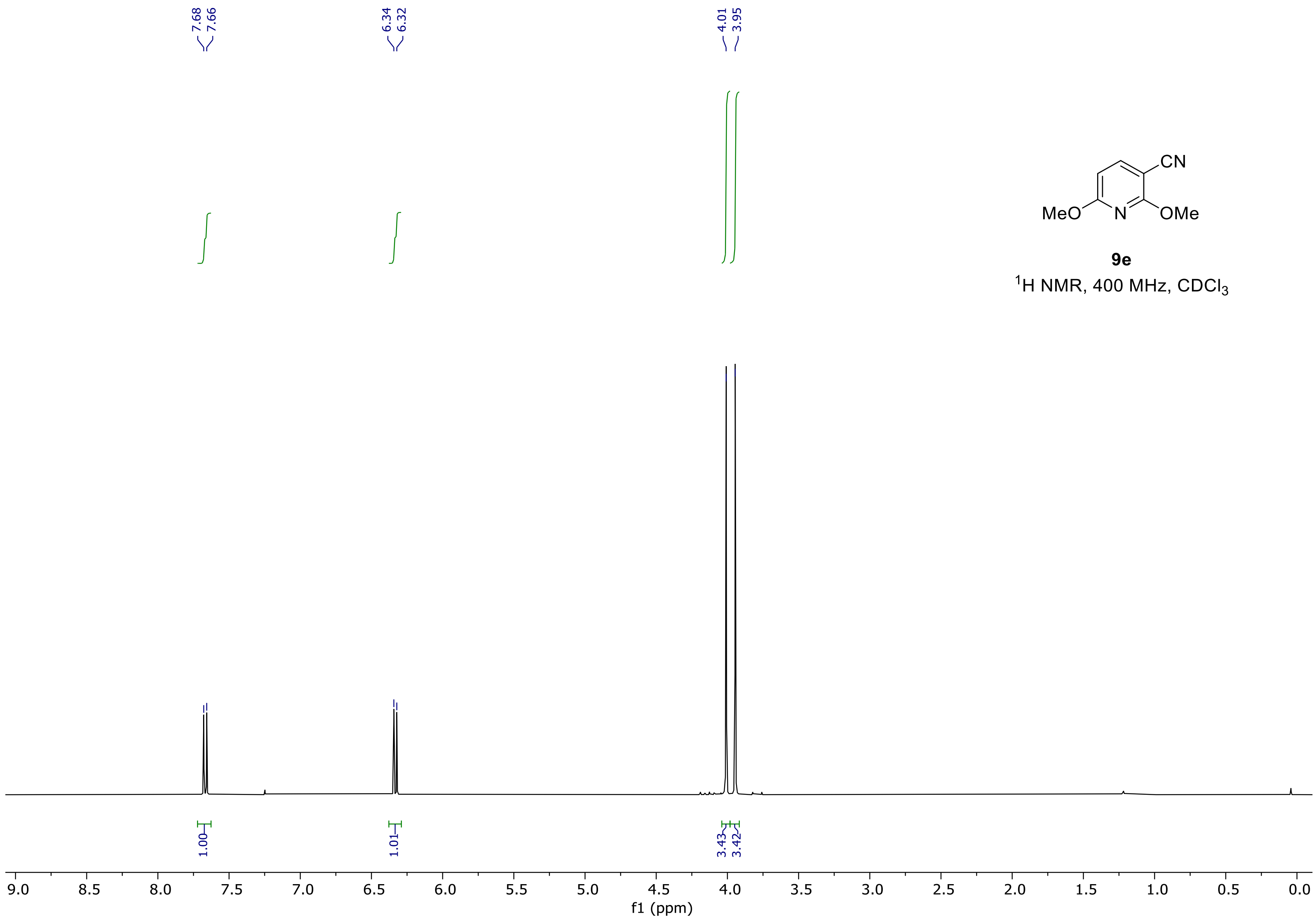
7.68
7.66
6.34
6.32
4.01
3.95
CN
MeO
N
OMe
9e
1H NMR, 400 MHz, CDCl3
1.00
1.01
3.43
3.42
9.0 8.5 8.0 7.5 7.0 6.5 6.0 5.5 5.0 4.5 4.0 3.5 3.0 2.5 2.0 1.5 1.0 0.5 0.0
f1 (ppm)

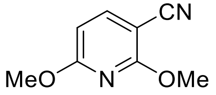

9e

^{13}C NMR, 101 MHz, $CDCl_3$

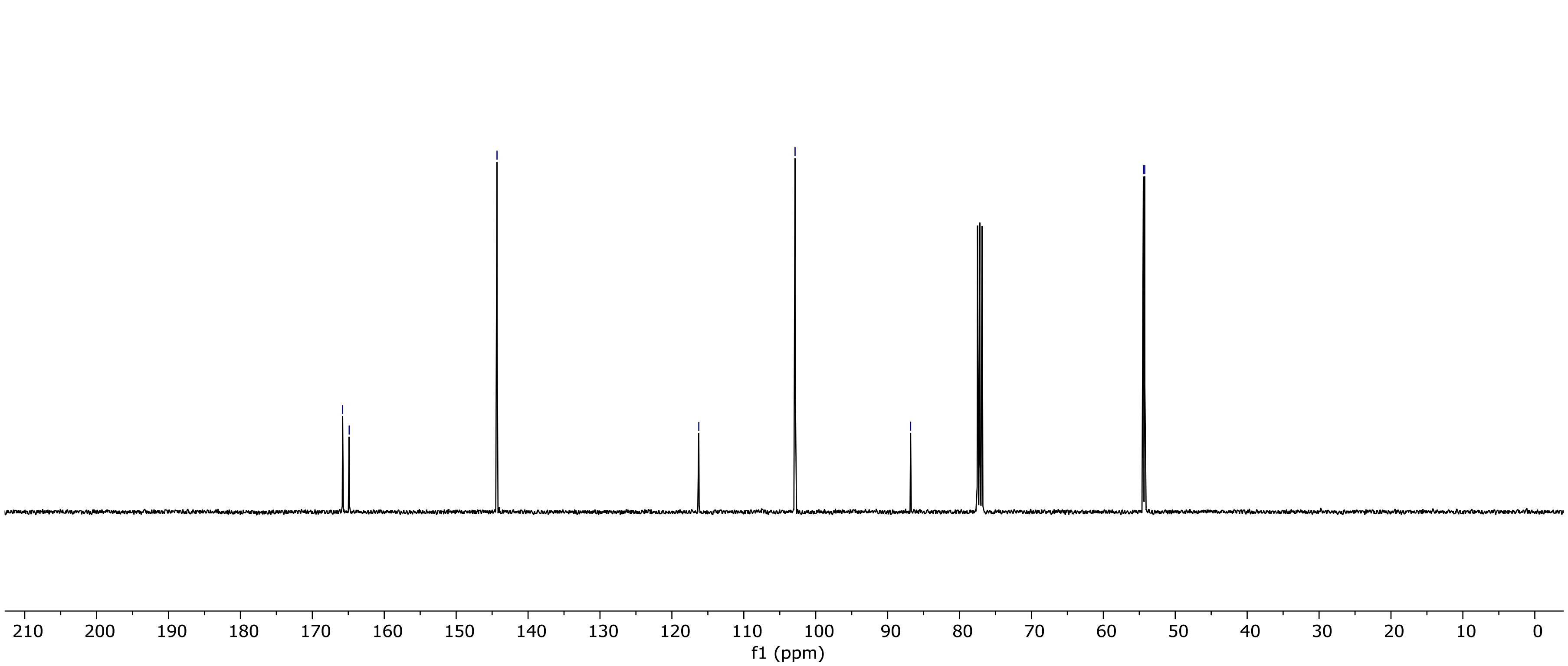

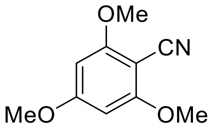

9f

^{1}H NMR, 400 MHz, $CDCl_3$

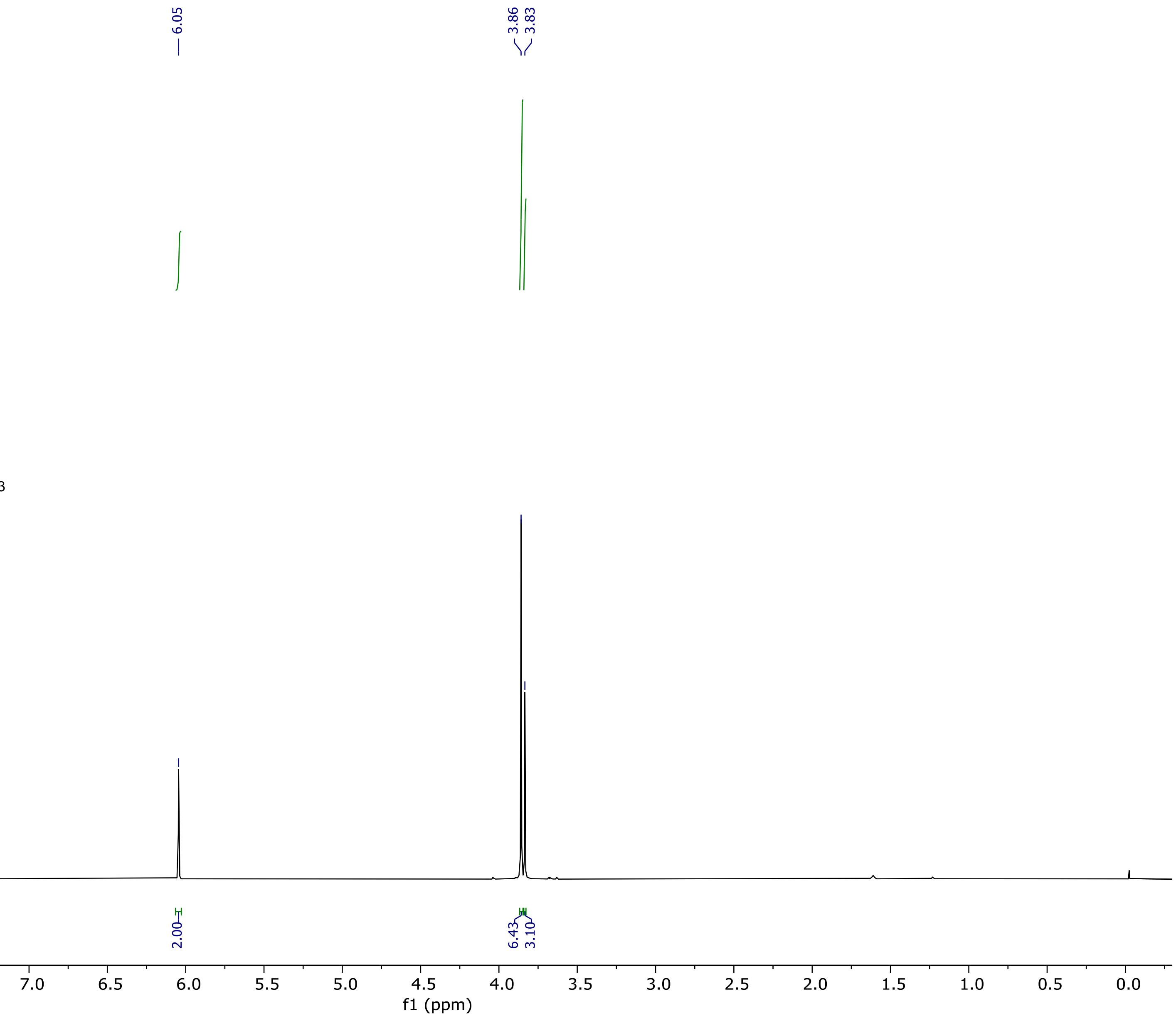

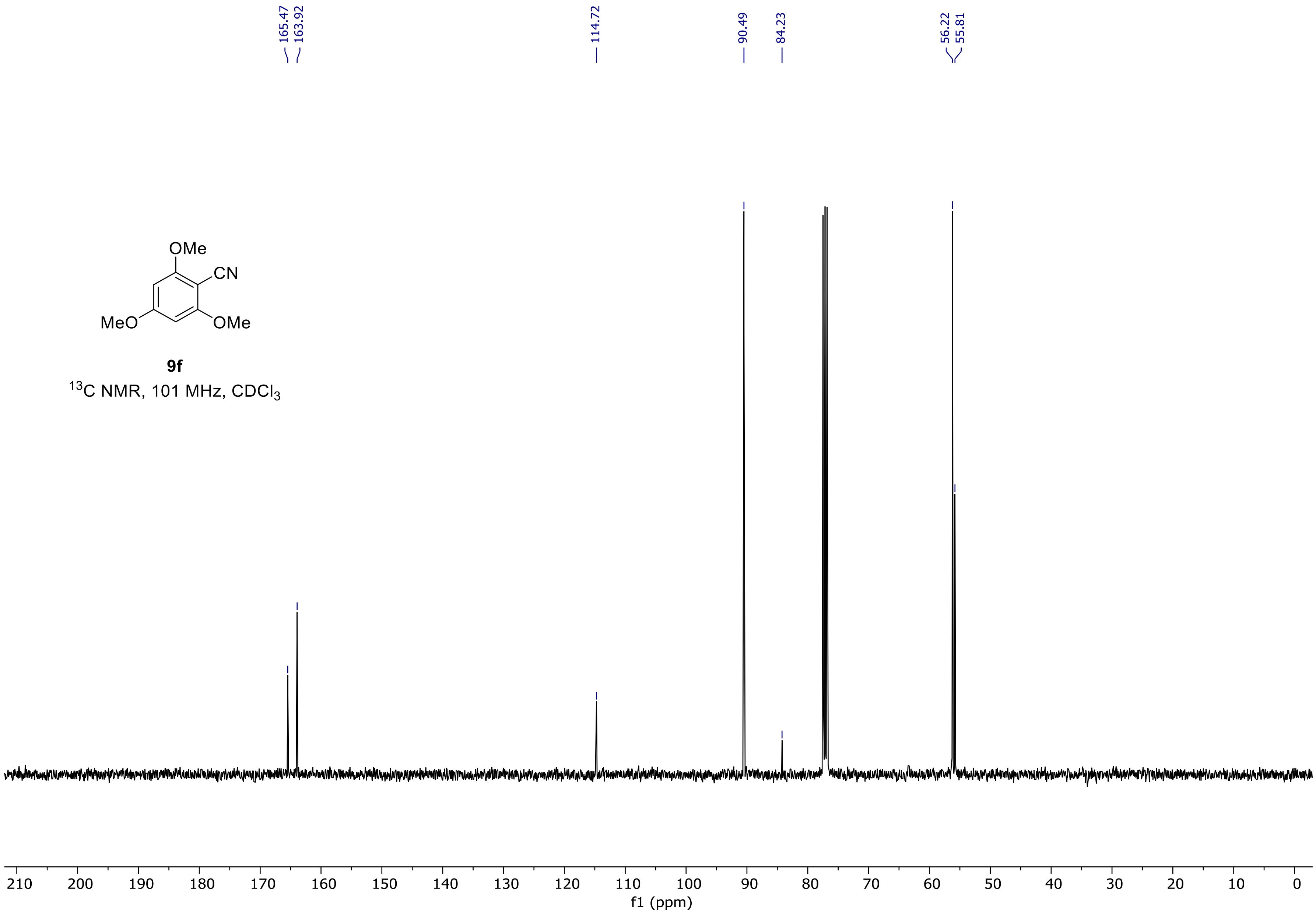
165.47
163.92
114.72
90.49
84.23
56.22
55.81
OMe
CN
MeO
OMe
9f
^{13}C NMR, 101 MHz, $CDCl_3$
210 200 190 180 170 160 150 140 130 120 110 100 90 80 70 60 50 40 30 20 10 0
f1 (ppm)

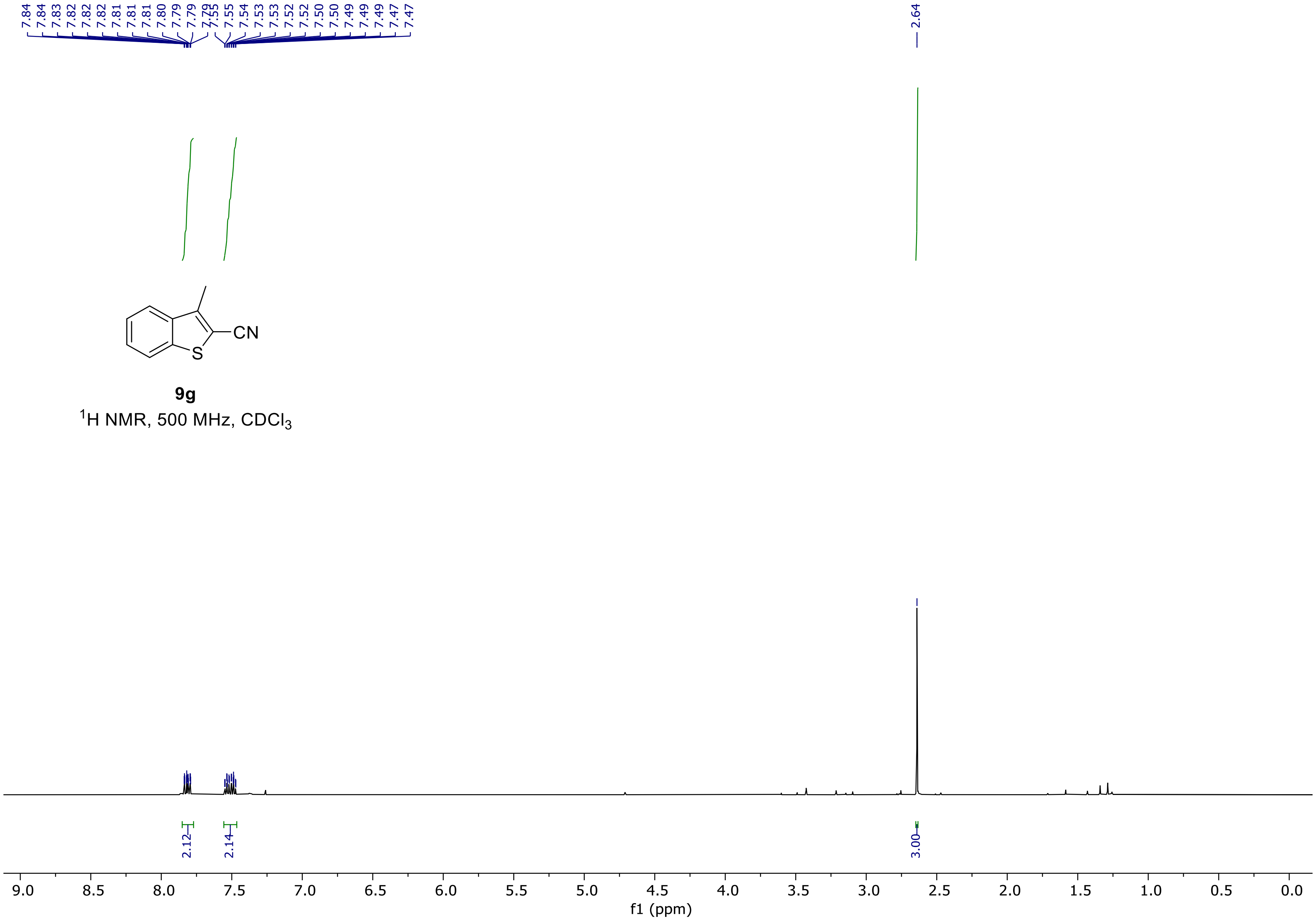

9g

^{1}H NMR, 500 MHz, $CDCl_3$

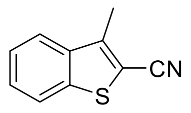

9g

^{13}C NMR, 126 MHz, $CDCl_3$

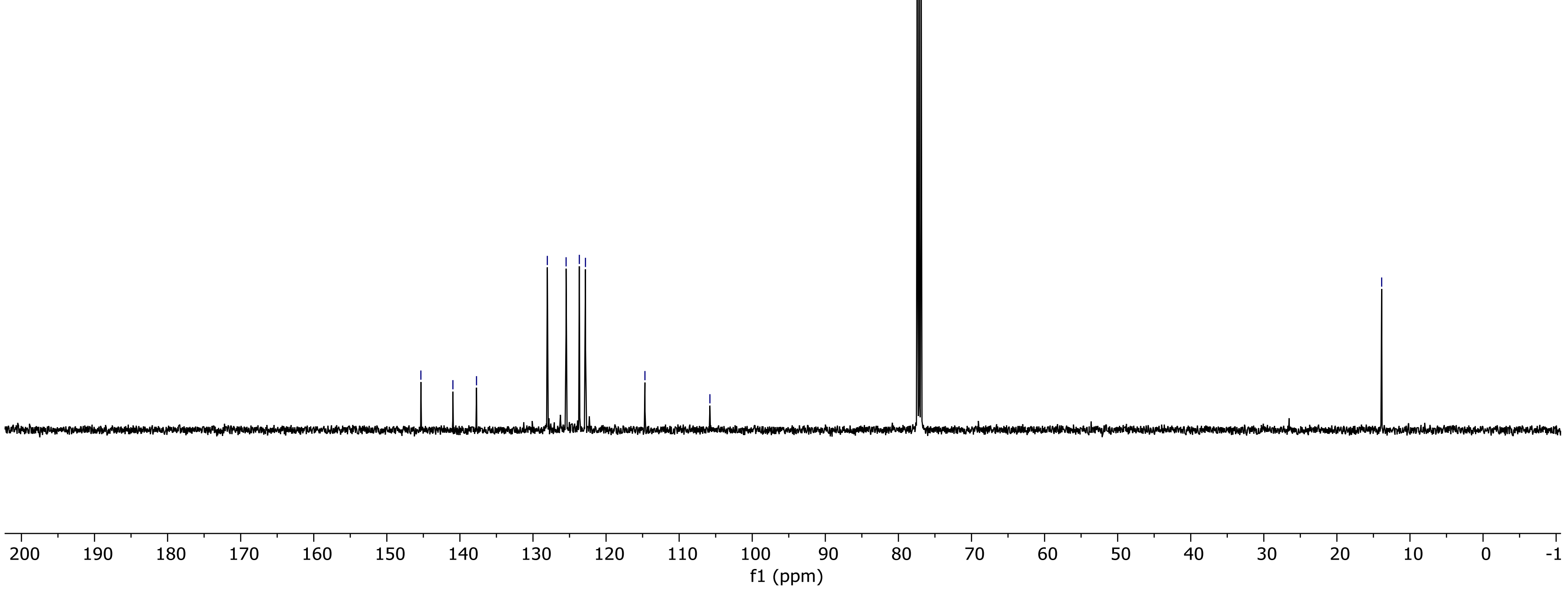

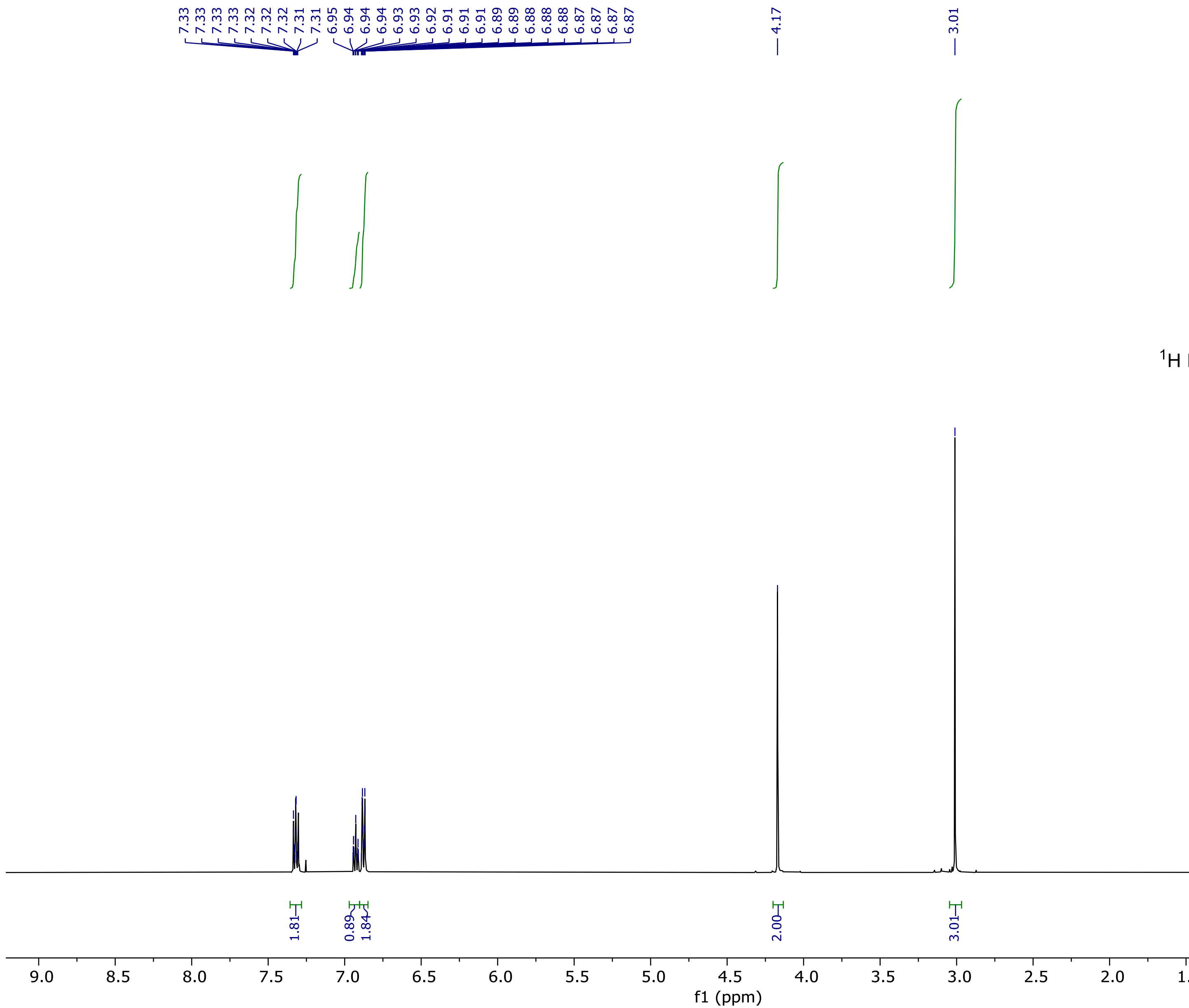

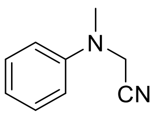

9h

^{1}H NMR, 500 MHz, $CDCl_3$

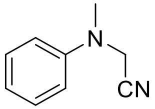

9h

^{13}C NMR, 126 MHz, $CDCl_3$

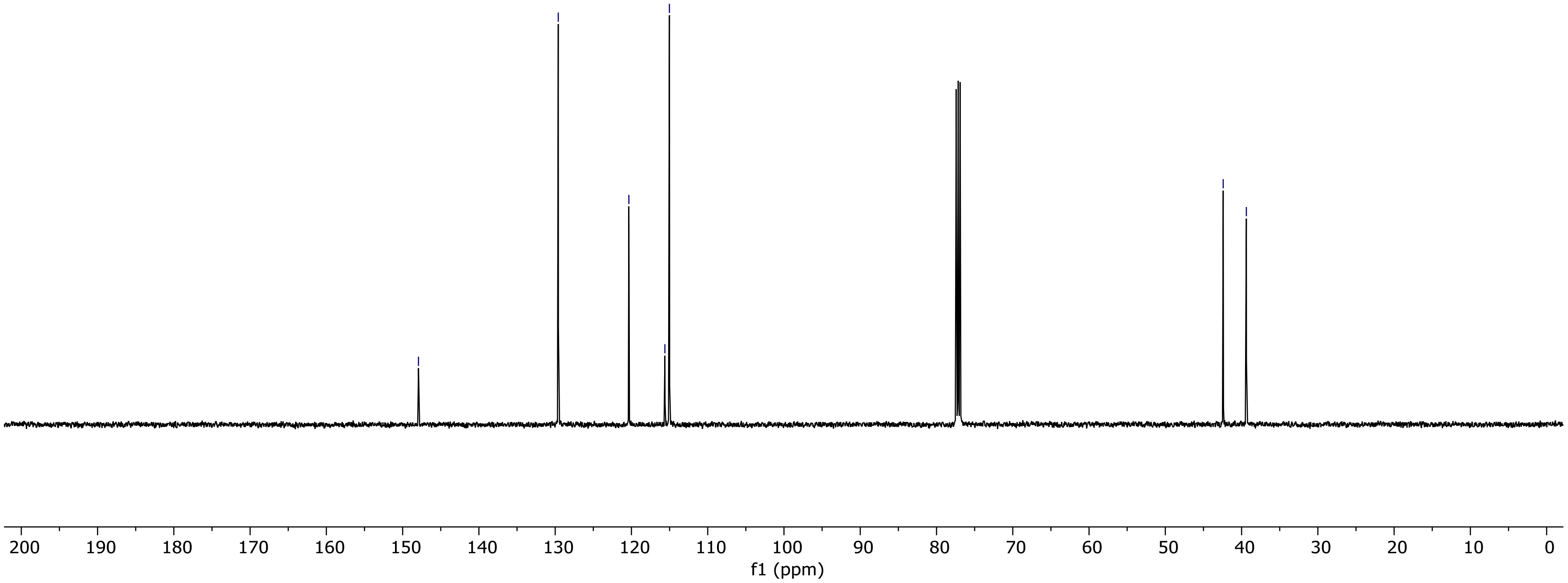

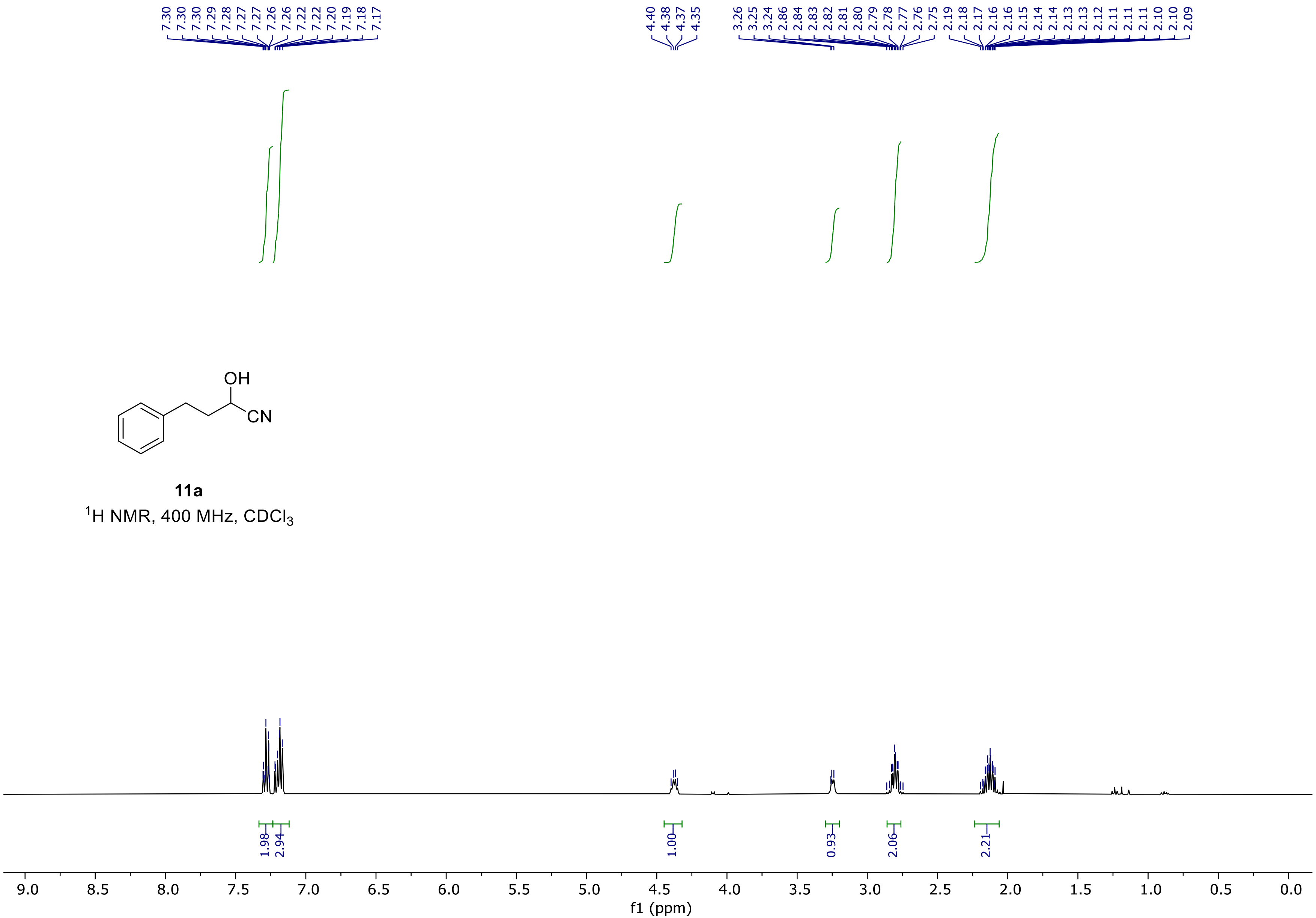

11a

^{1}H NMR, 400 MHz, CDCl$_3$

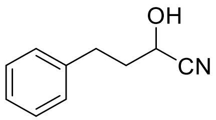

11a

^{13}C NMR, 101 MHz, $CDCl_3$

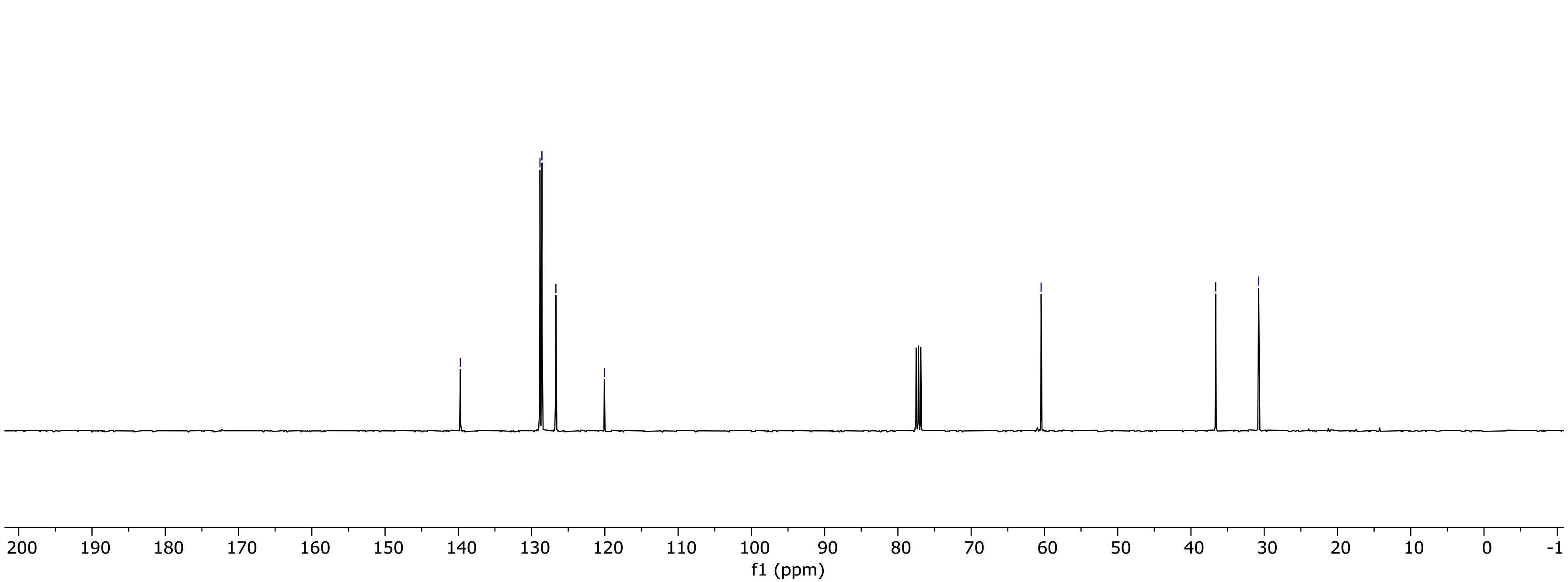

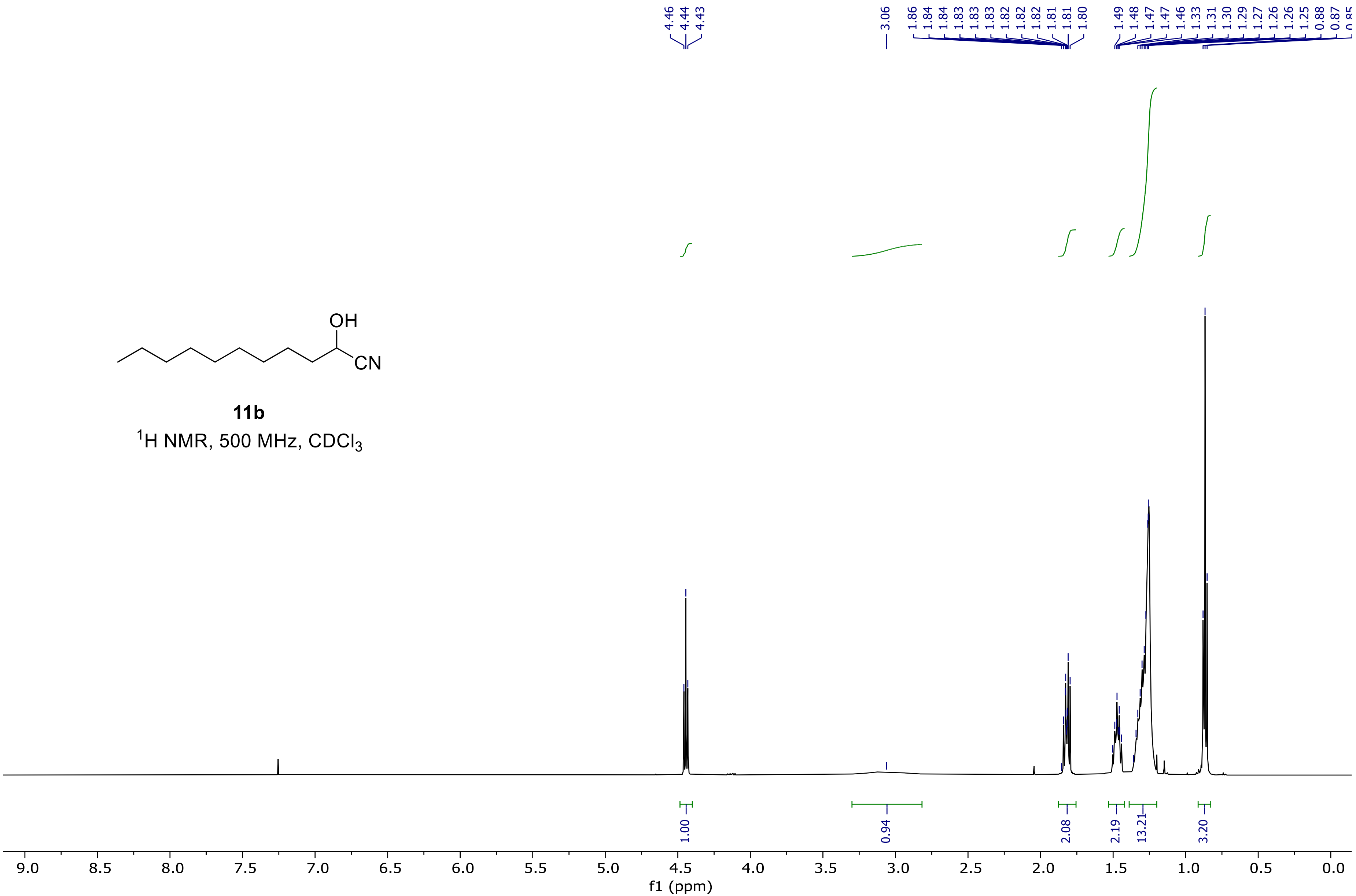

11b

^{1}H NMR, 500 MHz, $CDCl_3$

11b

^{13}C NMR, 126 MHz, $CDCl_3$

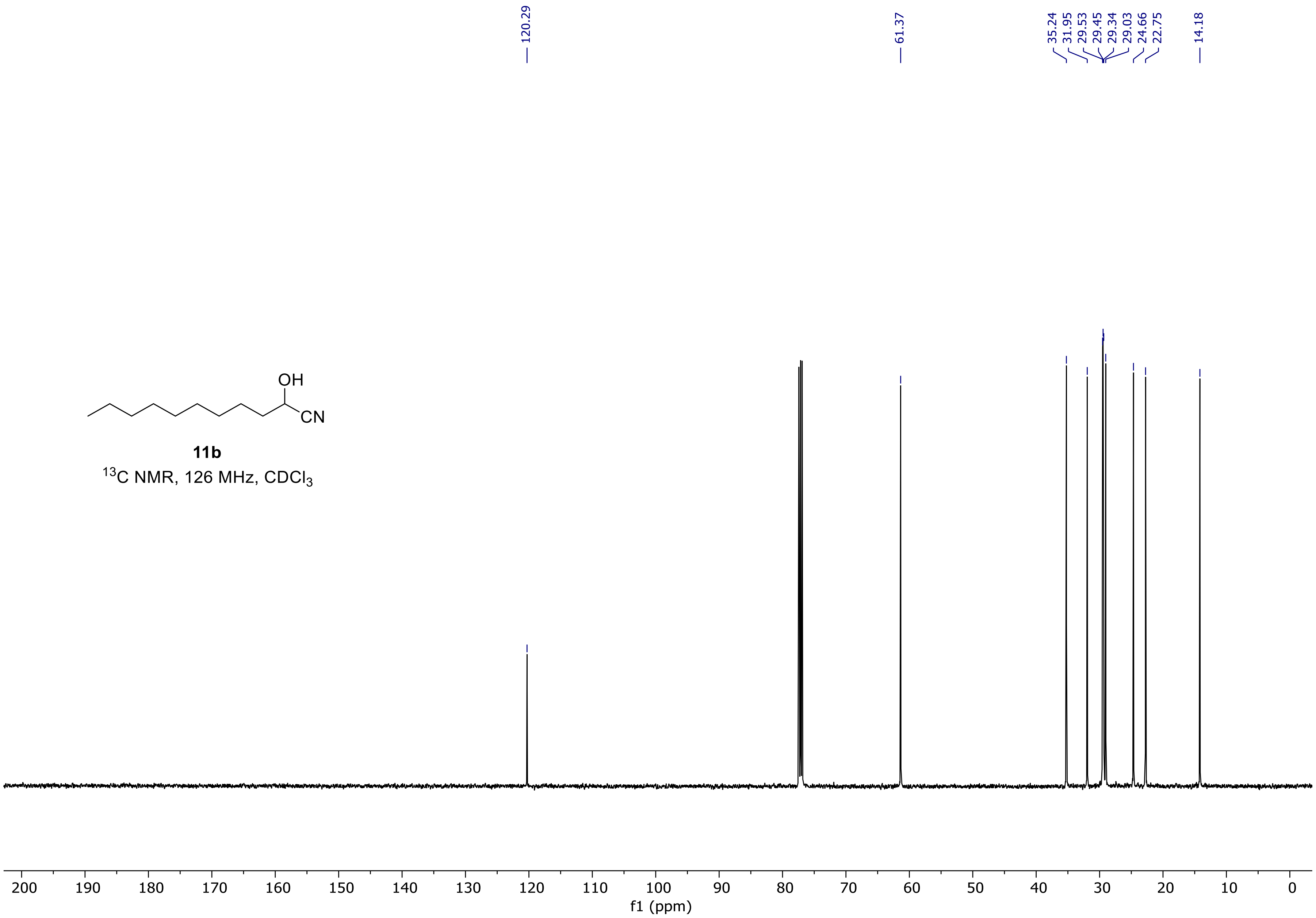

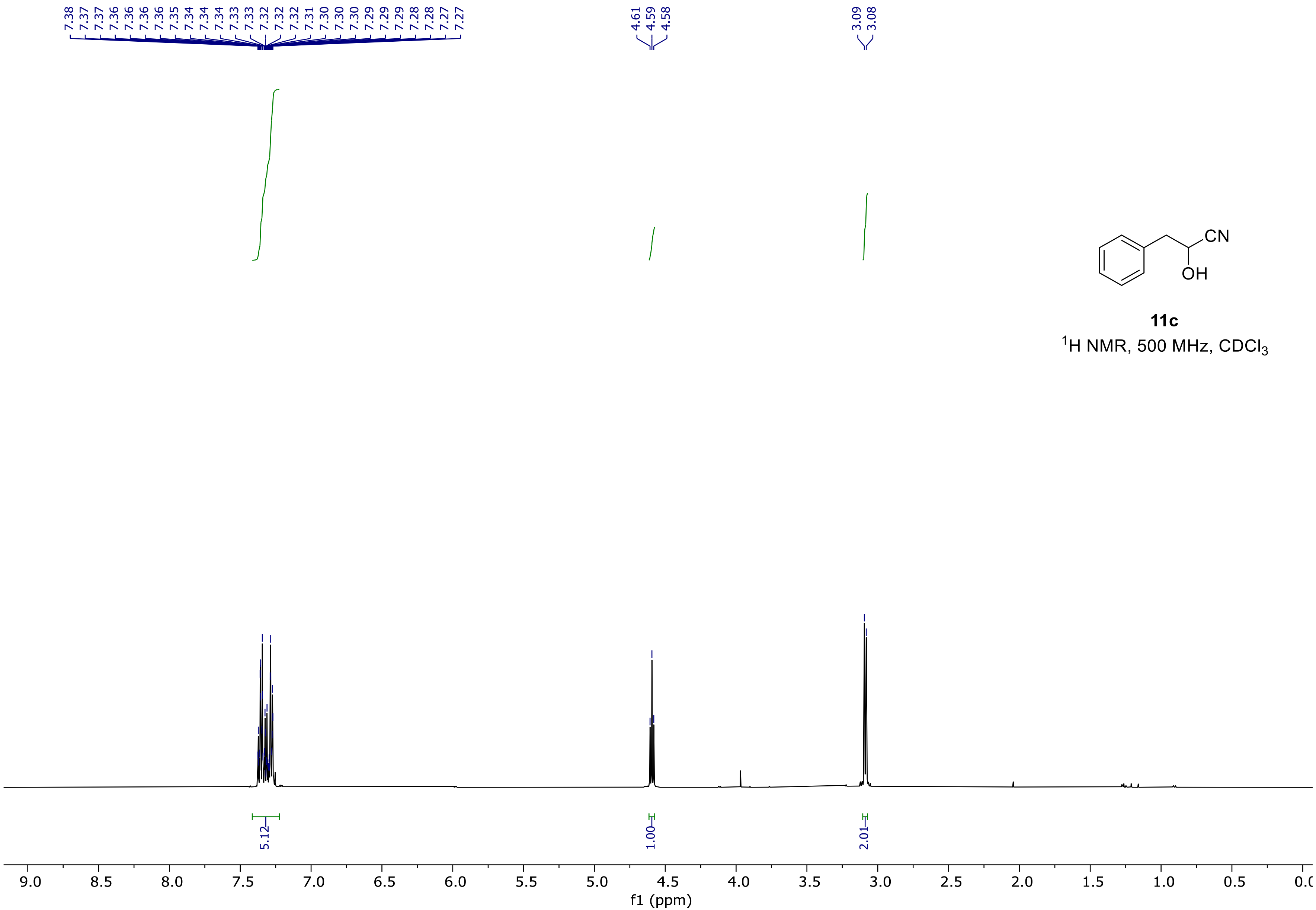

11c

^{1}H NMR, 500 MHz, $CDCl_3$

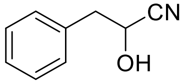

11c

^{13}C NMR, 126 MHz, $CDCl_3$

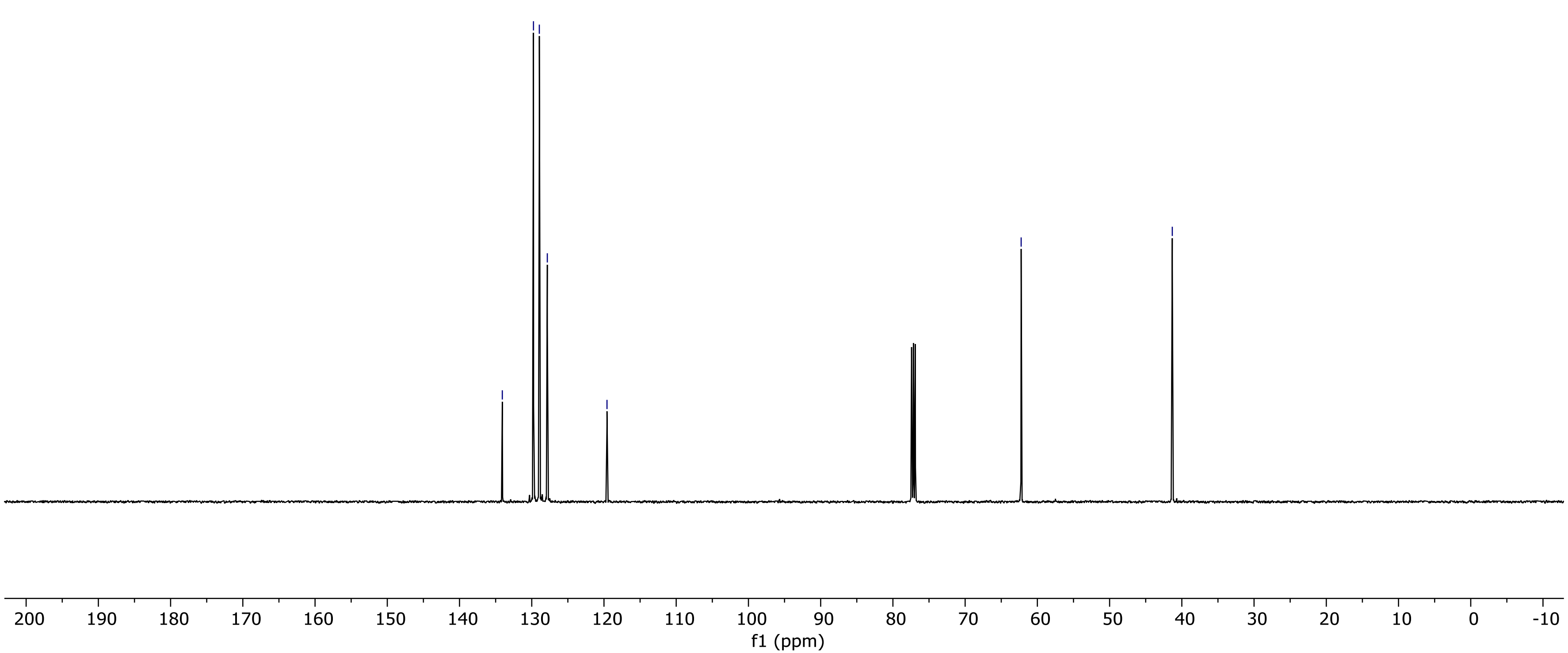

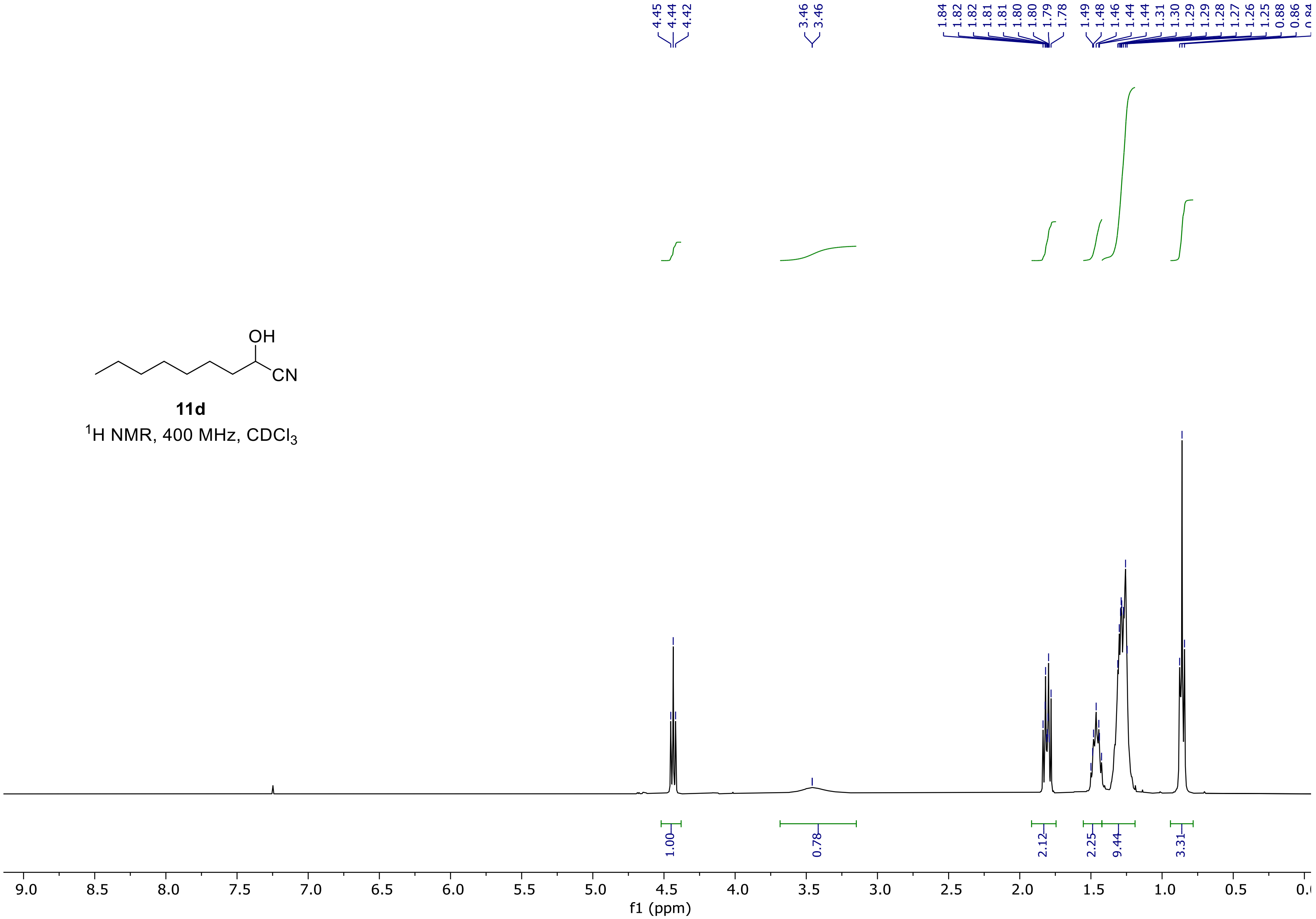
OH
CN
11d
1H NMR, 400 MHz, CDCl3
4.45
4.44
4.42
3.46
3.46
1.84
1.82
1.82
1.81
1.81
1.80
1.80
1.79
1.78
1.49
1.48
1.46
1.44
1.44
1.31
1.30
1.29
1.29
1.28
1.27
1.26
1.25
0.88
0.86
1.00
0.78
2.12
2.25
9.44
3.31
9.0
8.5
8.0
7.5
7.0
6.5
6.0
5.5
5.0
4.5
4.0
3.5
3.0
2.5
2.0
1.5
1.0
0.5
f1 (ppm)

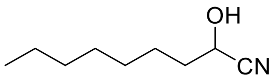

11d

^{13}C NMR, 101 MHz, $CDCl_3$

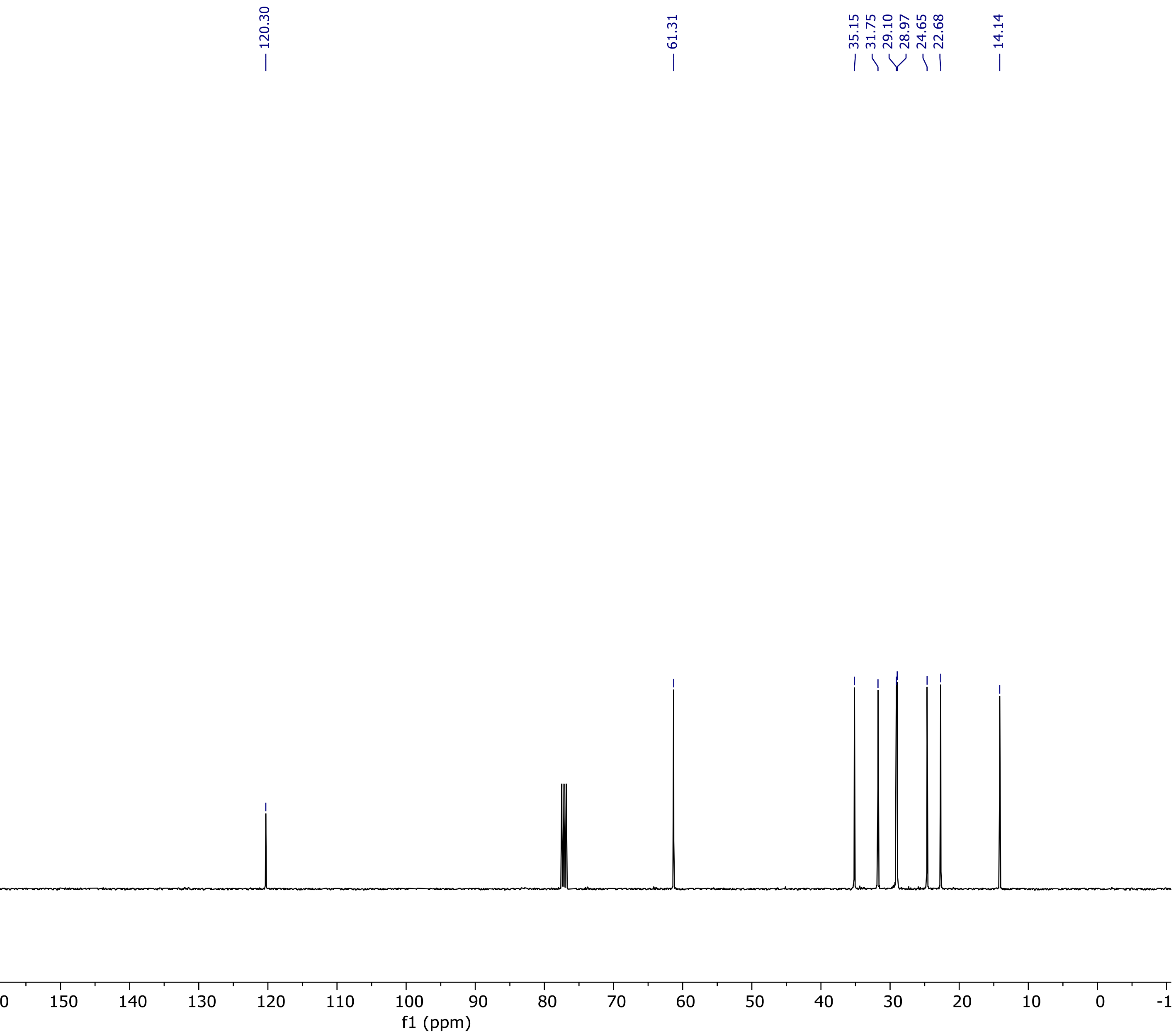

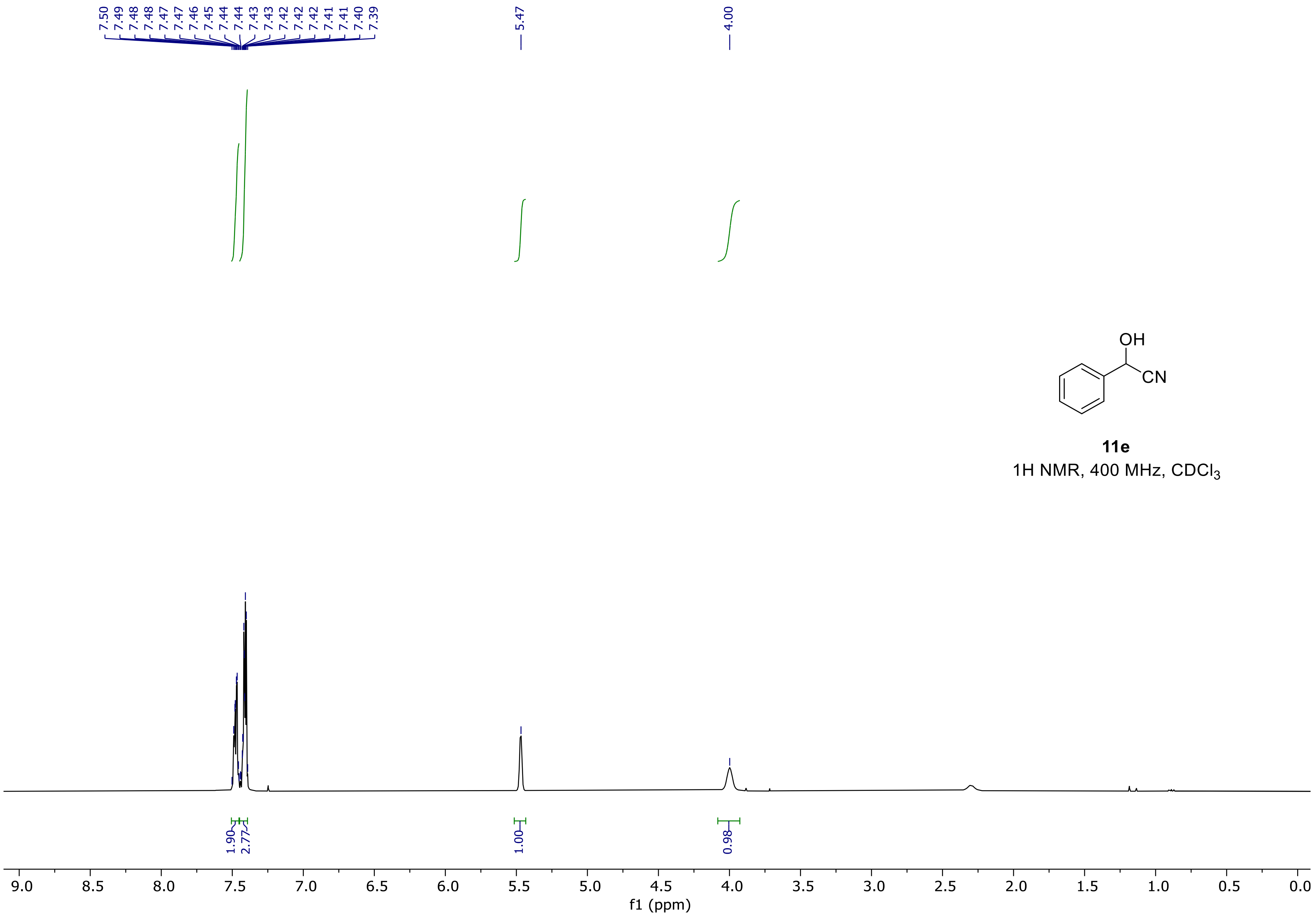

11e

1H NMR, 400 MHz, $CDCl_3$

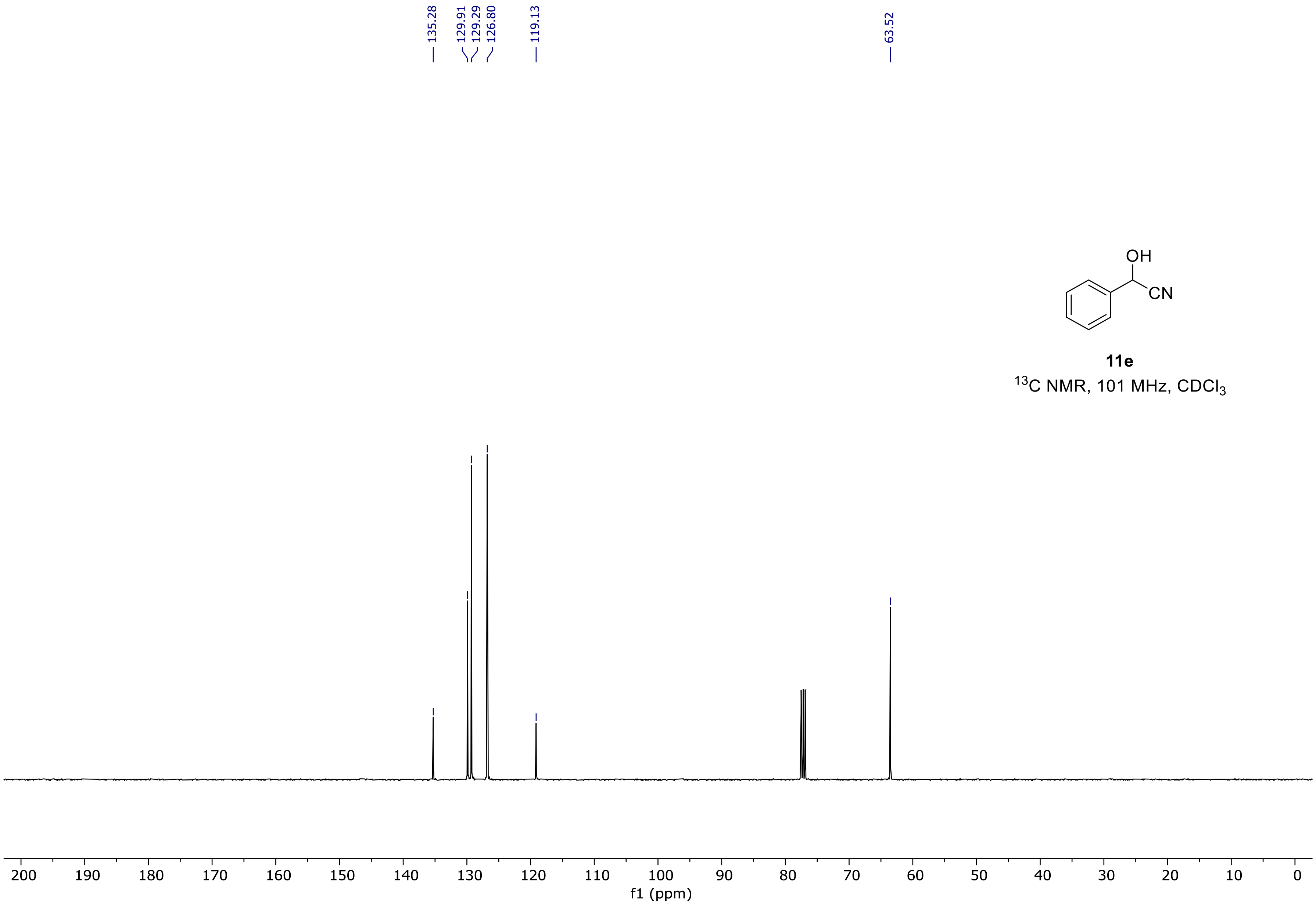

11e

^{13}C NMR, 101 MHz, $CDCl_3$

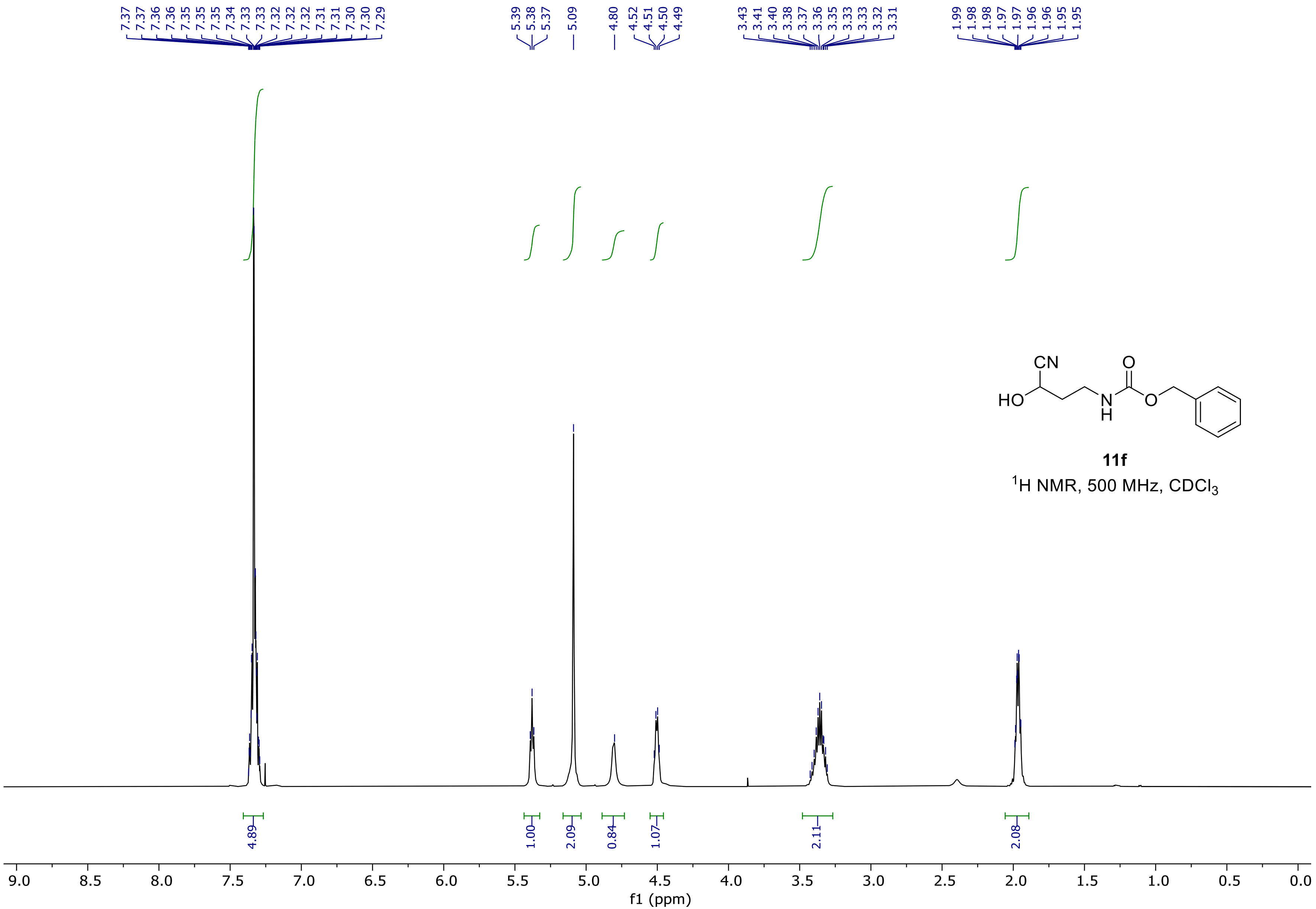
7.37
7.37
7.36
7.36
7.35
7.35
7.35
7.34
7.33
7.33
7.32
7.32
7.32
7.31
7.31
7.30
7.30
7.29
5.39
5.38
5.37
5.09
4.80
4.52
4.51
4.50
4.49
3.43
3.41
3.40
3.38
3.37
3.36
3.35
3.33
3.33
3.32
3.31
1.99
1.98
1.98
1.97
1.97
1.96
1.96
1.95
1.95
CN
O
HO
N
H
O
11f
1H NMR, 500 MHz, CDCl3
4.89
1.00
2.09
0.84
1.07
2.11
2.08
9.0
8.5
8.0
7.5
7.0
6.5
6.0
5.5
5.0
4.5
4.0
3.5
3.0
2.5
2.0
1.5
1.0
0.5
0.0
f1 (ppm)

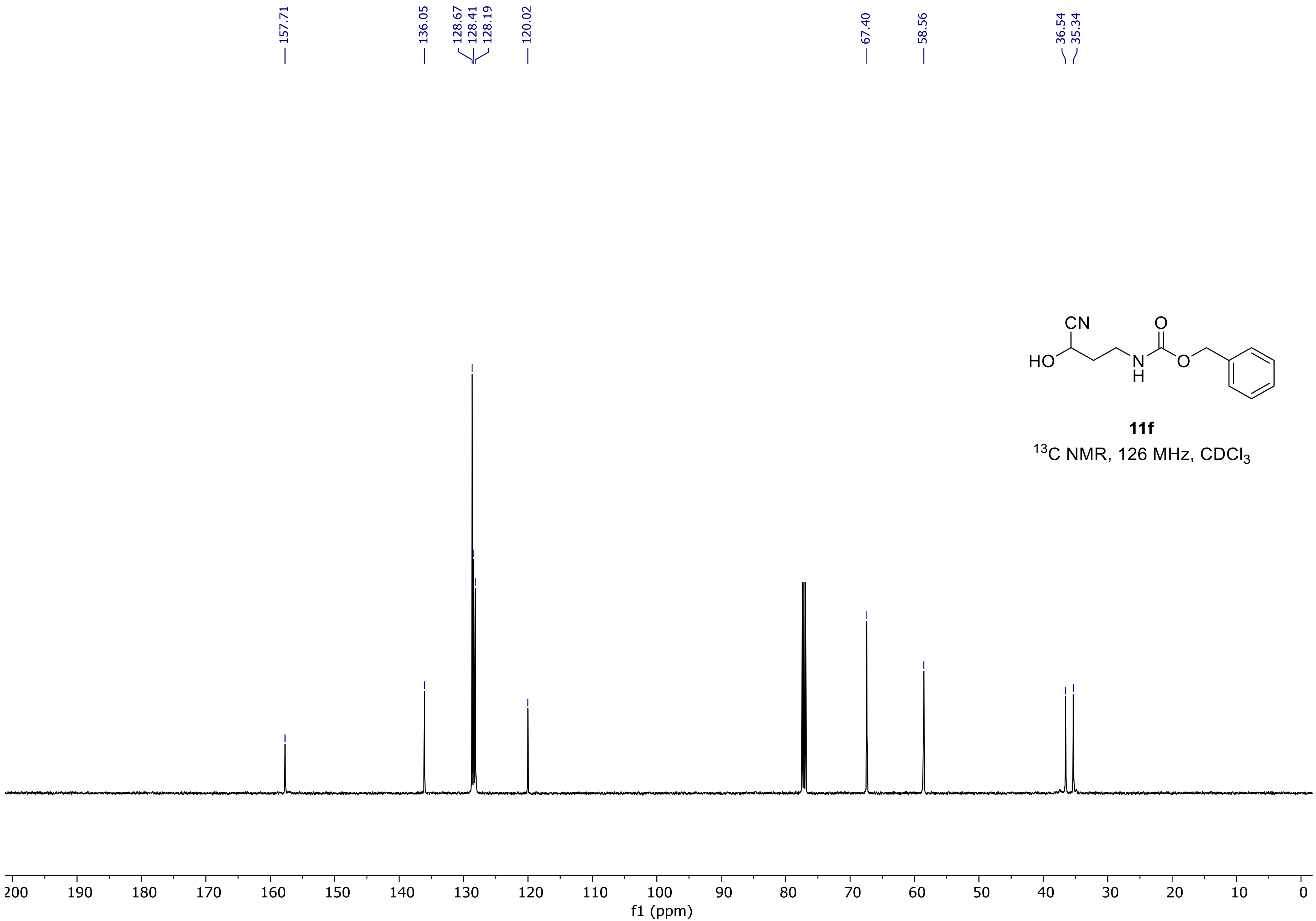
157.71
136.05
128.67
128.41
128.19
120.02
67.40
58.56
36.54
35.34
CN
HO
N
H
O
O
11f
^{13}C NMR, 126 MHz, $CDCl_3$
200 190 180 170 160 150 140 130 120 110 100 90 80 70 60 50 40 30 20 10 0
f1 (ppm)

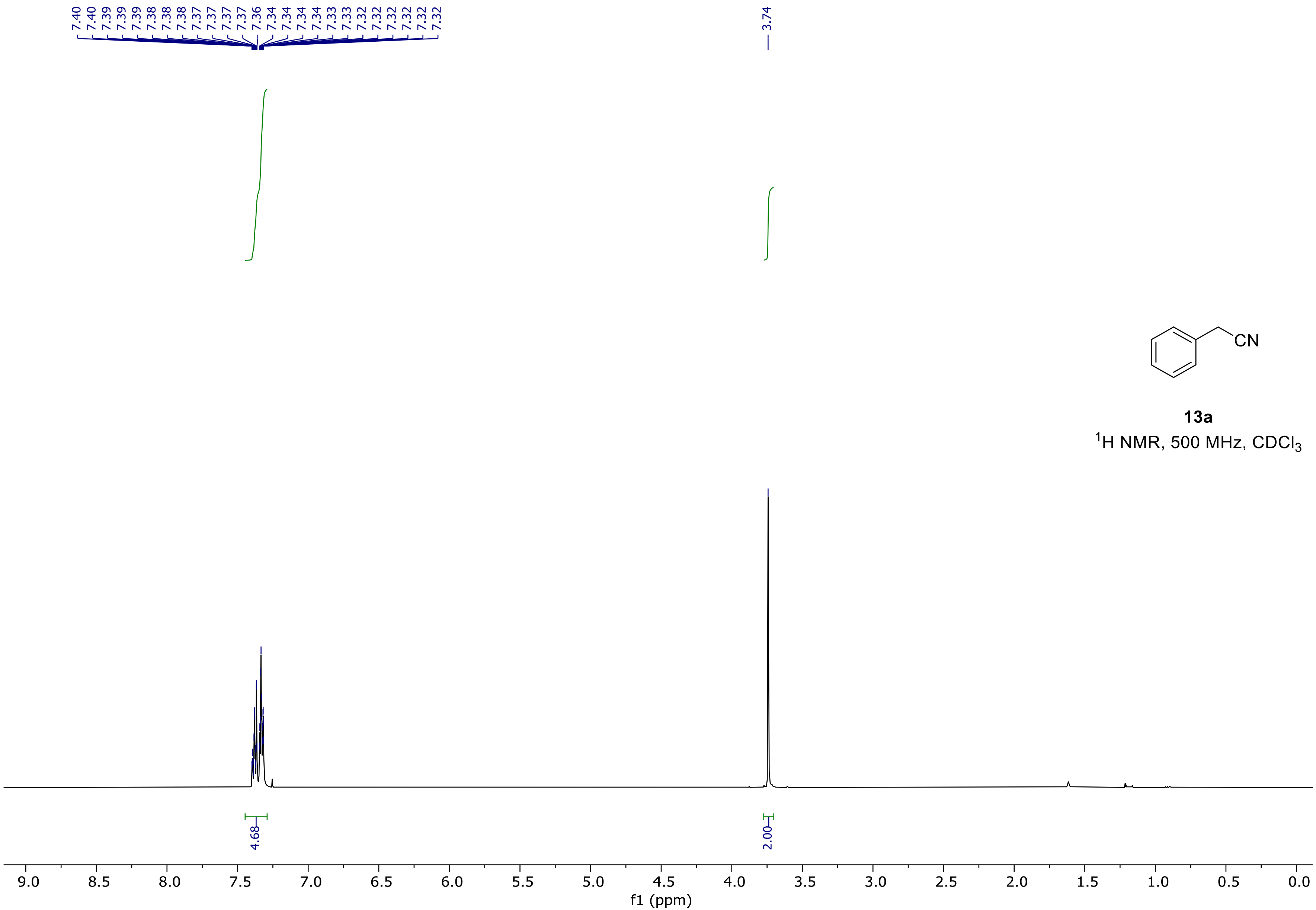

13a

^{1}H NMR, 500 MHz, CDCl$_3$

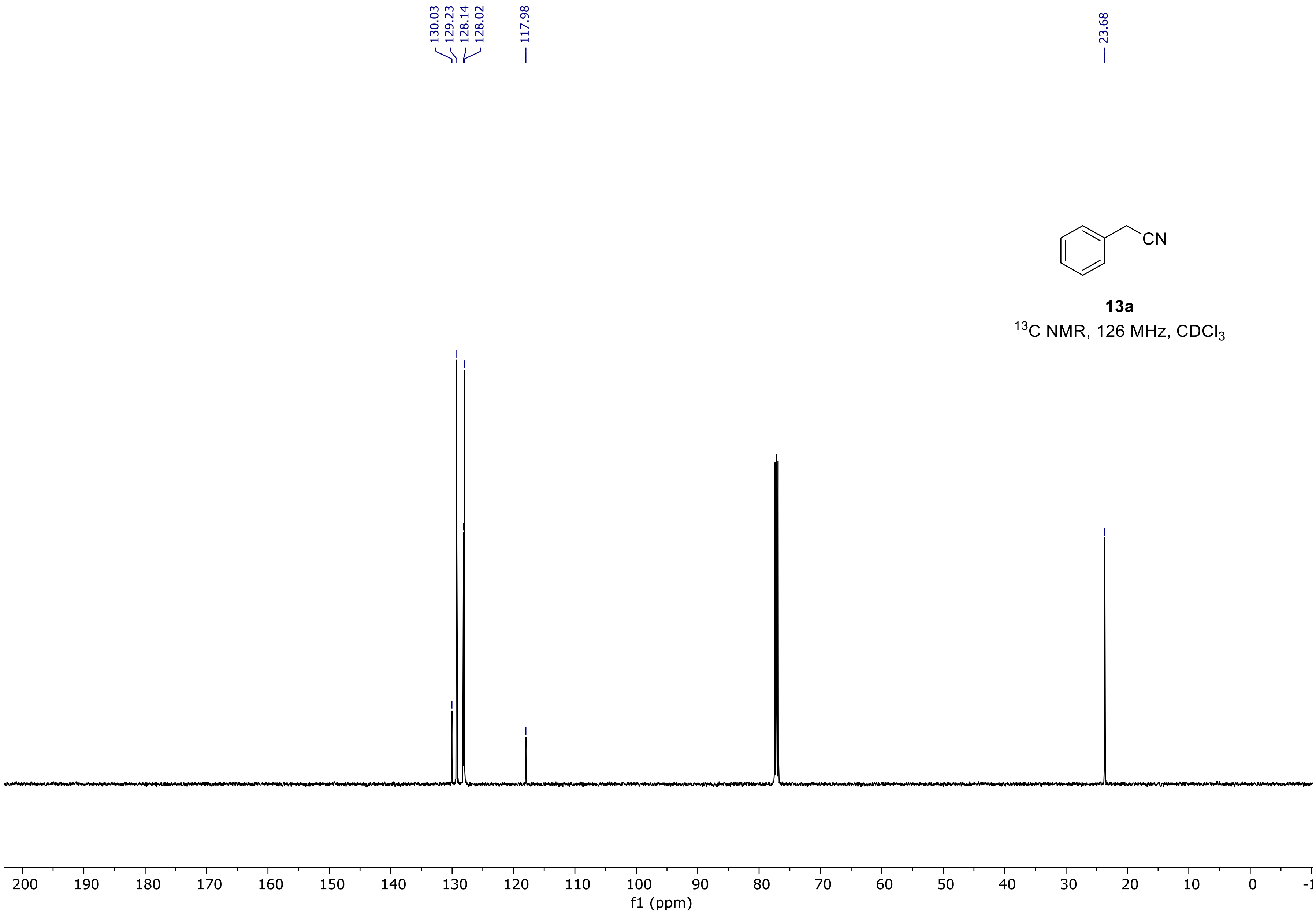

13a

^{13}C NMR, 126 MHz, $CDCl_3$

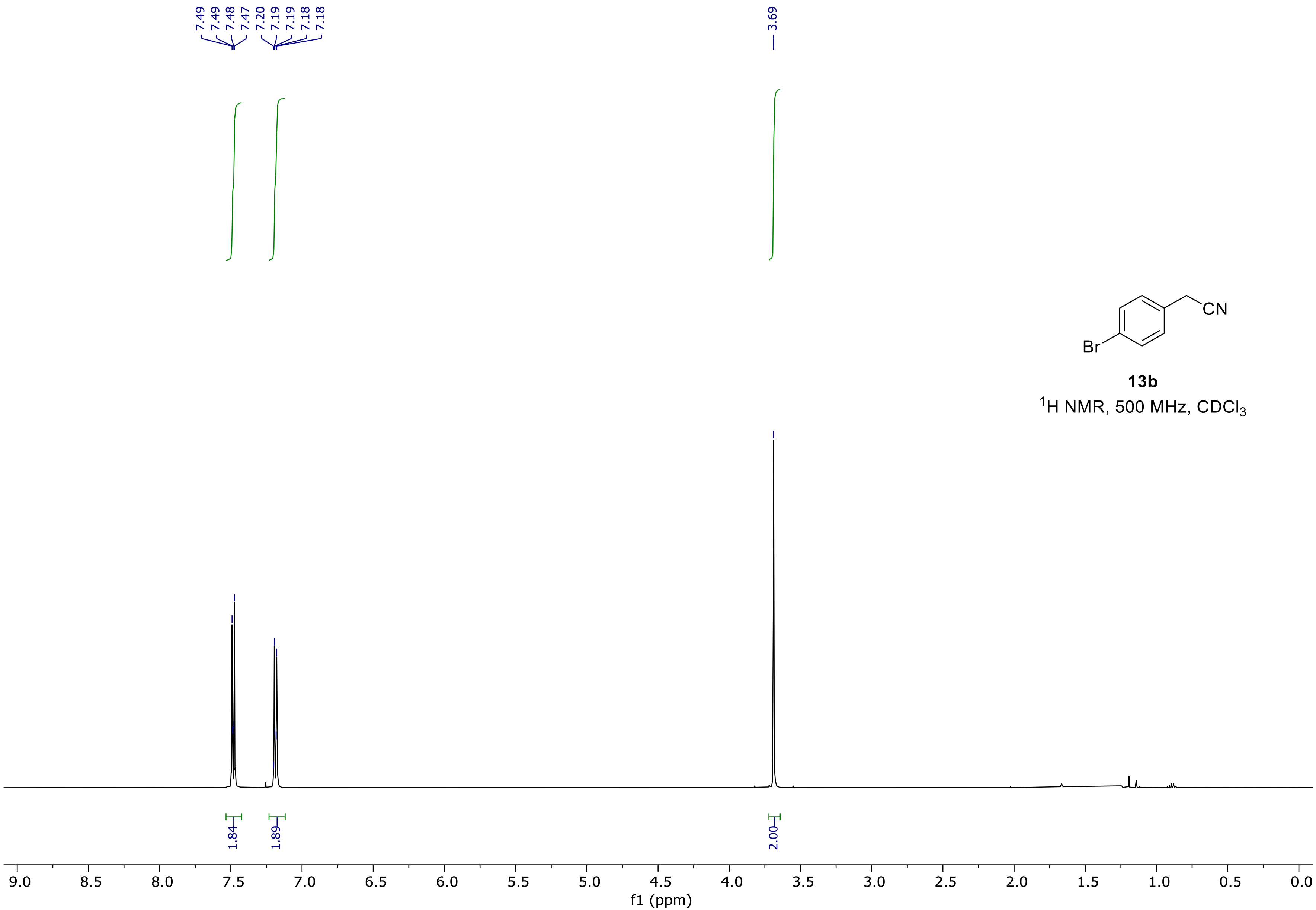
7.49
7.49
7.48
7.47
7.20
7.19
7.19
7.18
7.18
3.69
CN
Br
13b
^{1}H NMR, 500 MHz, $CDCl_3$
1.84
1.89
2.00
9.0 8.5 8.0 7.5 7.0 6.5 6.0 5.5 5.0 4.5 4.0 3.5 3.0 2.5 2.0 1.5 1.0 0.5 0.0
f1 (ppm)

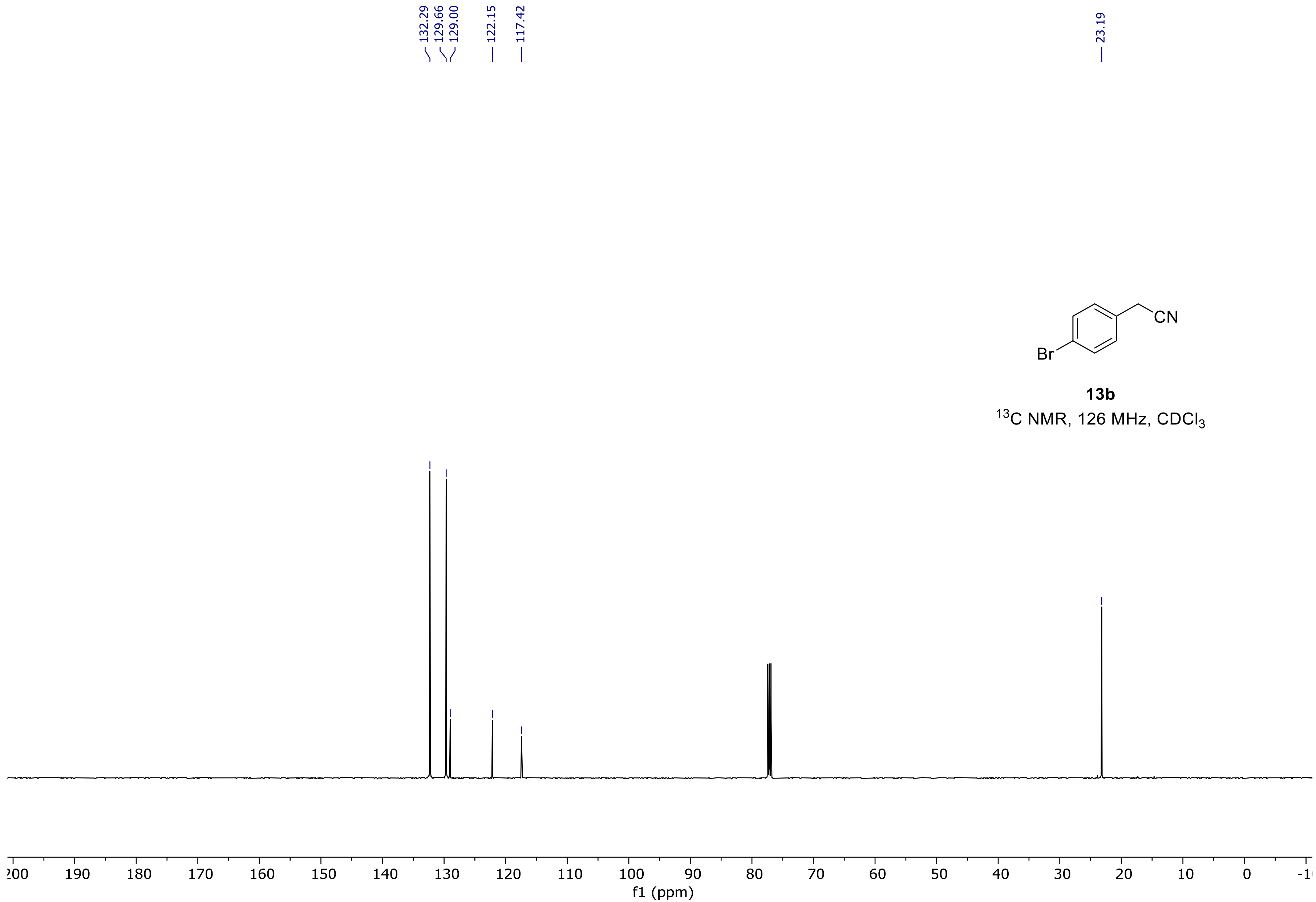

13b

^{13}C NMR, 126 MHz, $CDCl_3$

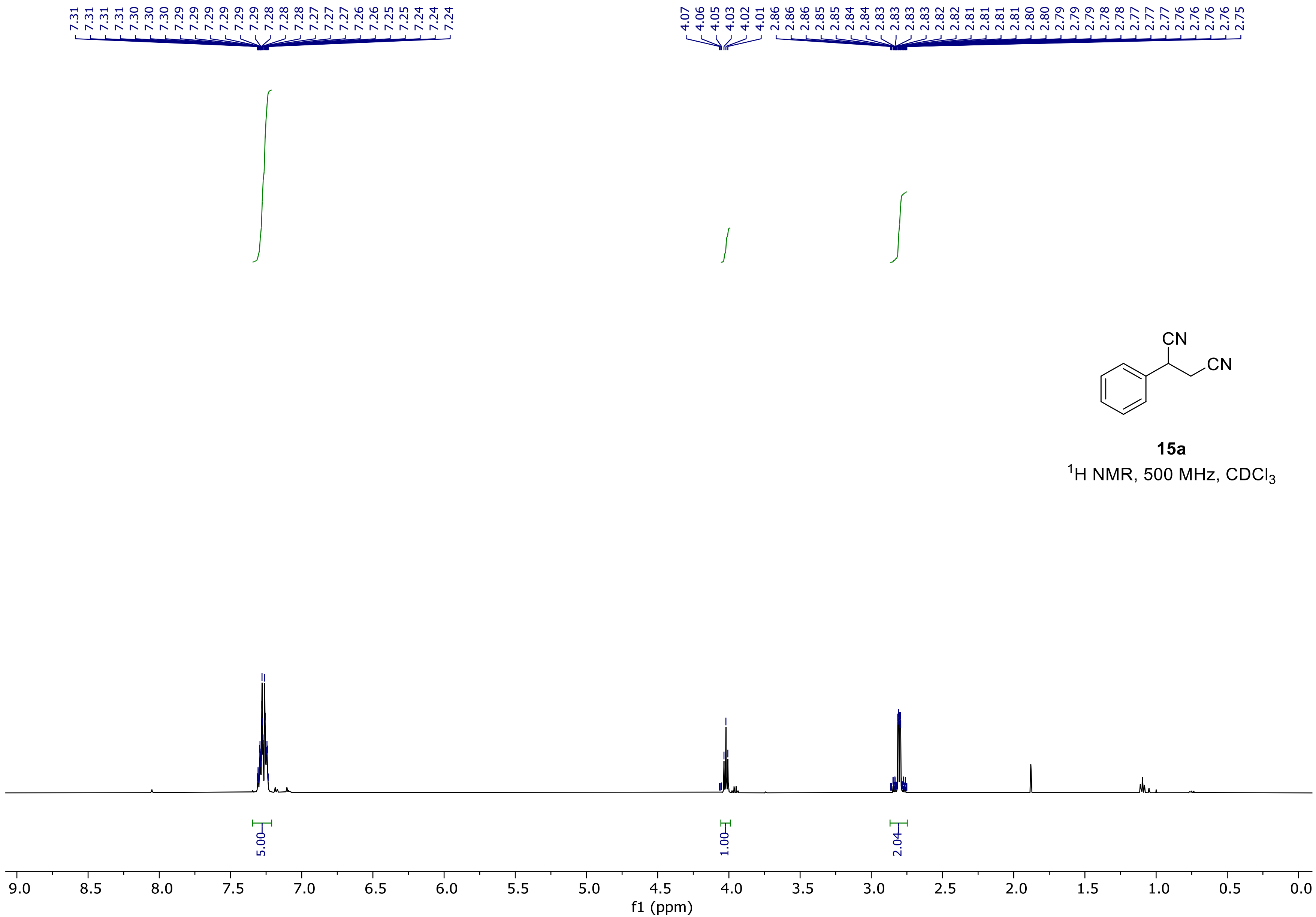

15a

^{1}H NMR, 500 MHz, $CDCl_3$

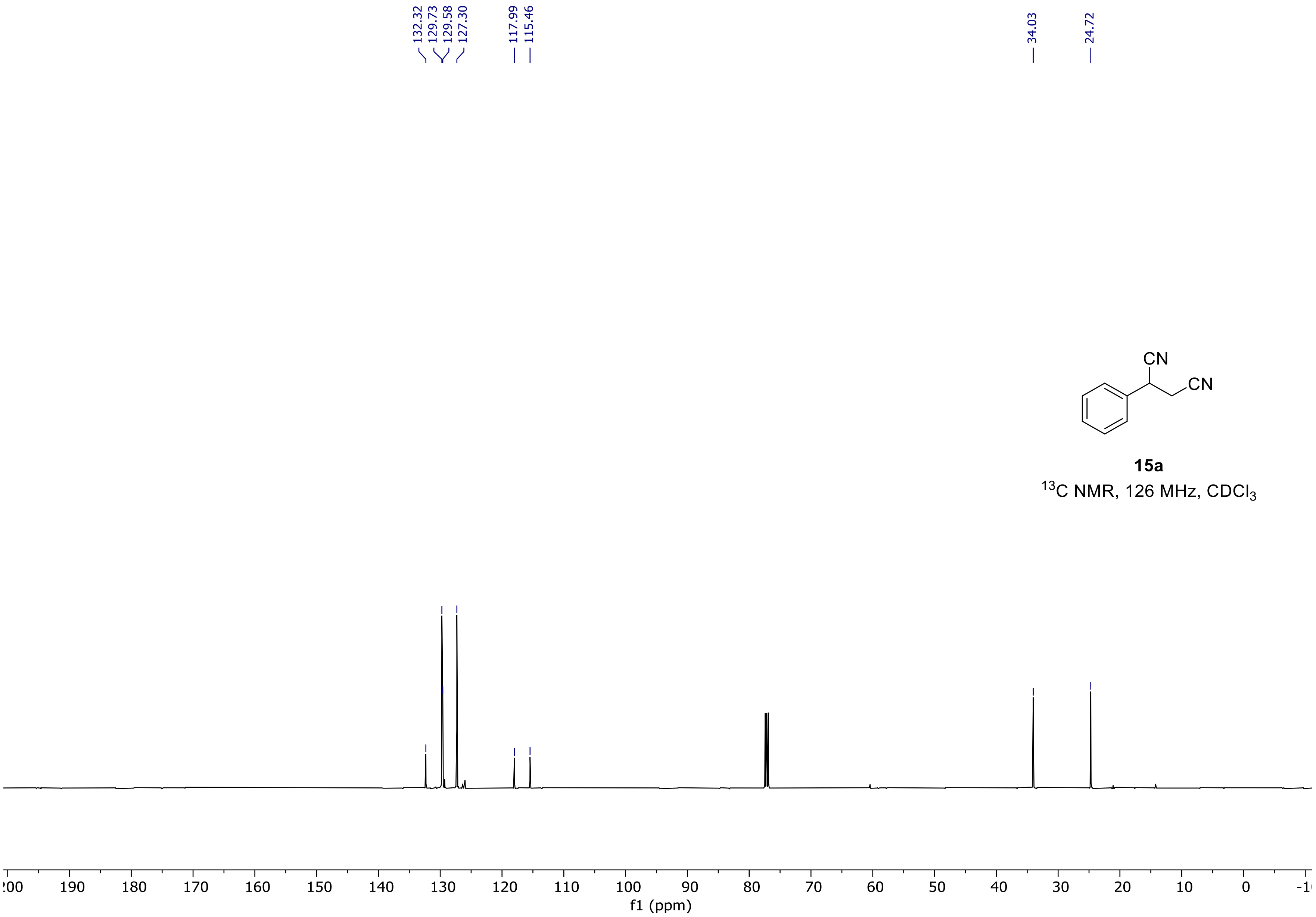

15a

^{13}C NMR, 126 MHz, $CDCl_3$

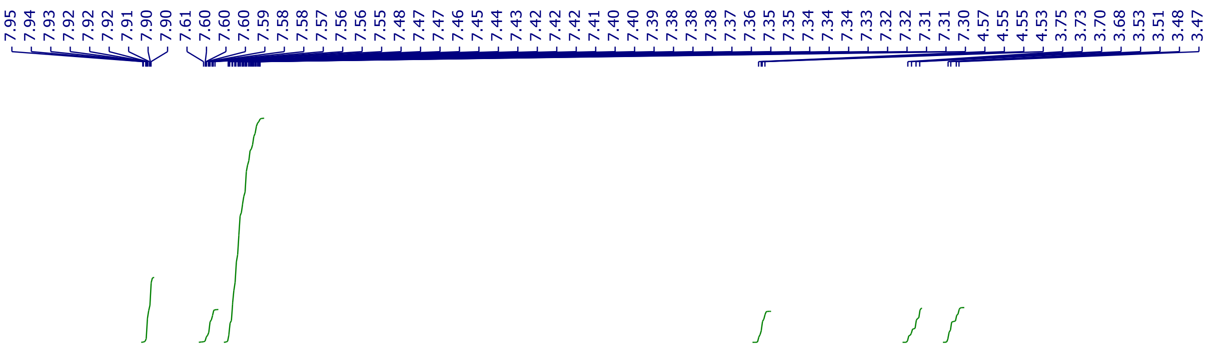

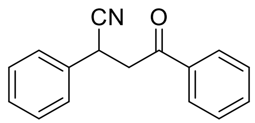

15b

^1H NMR, 400 MHz, $CDCl_3$

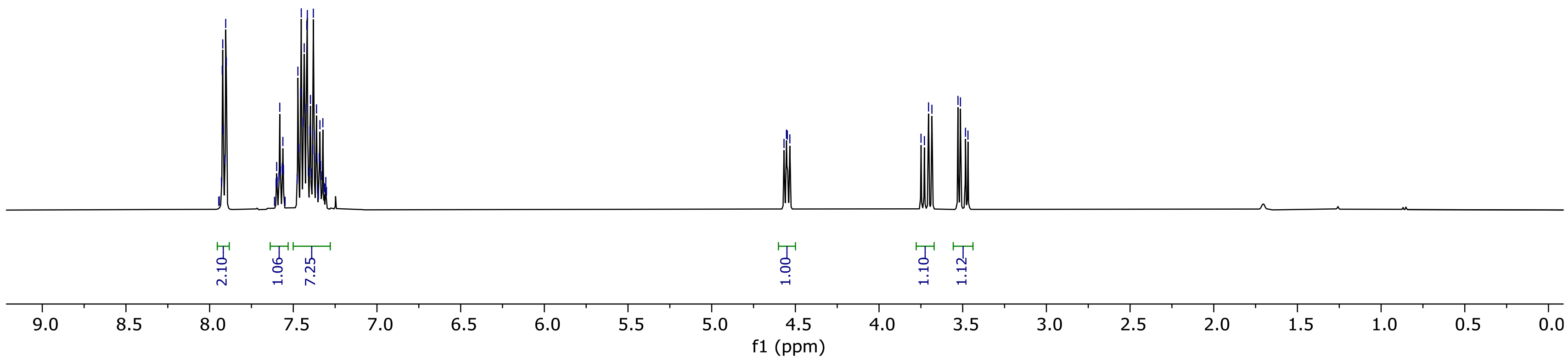

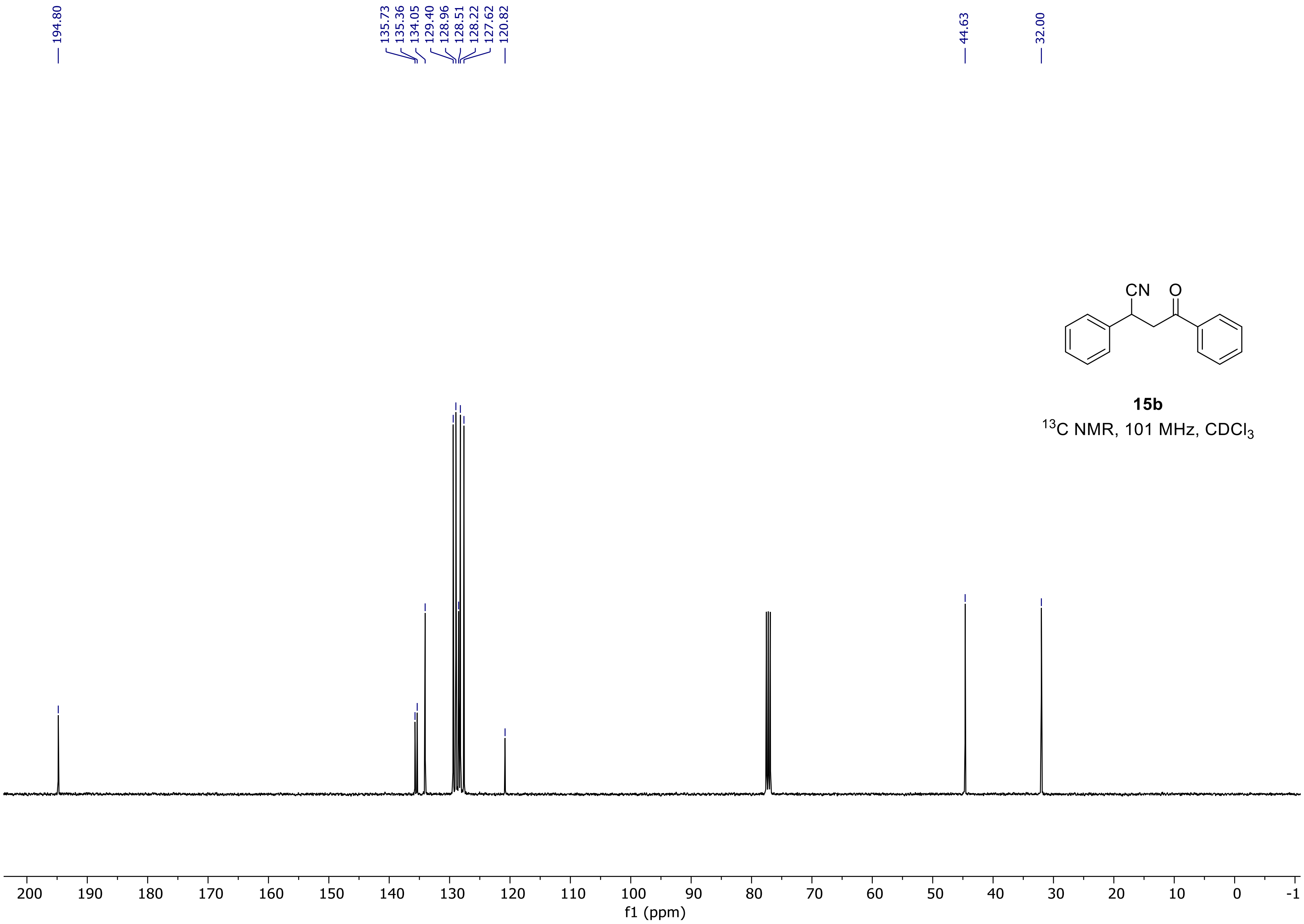

15b

^{13}C NMR, 101 MHz, $CDCl_3$

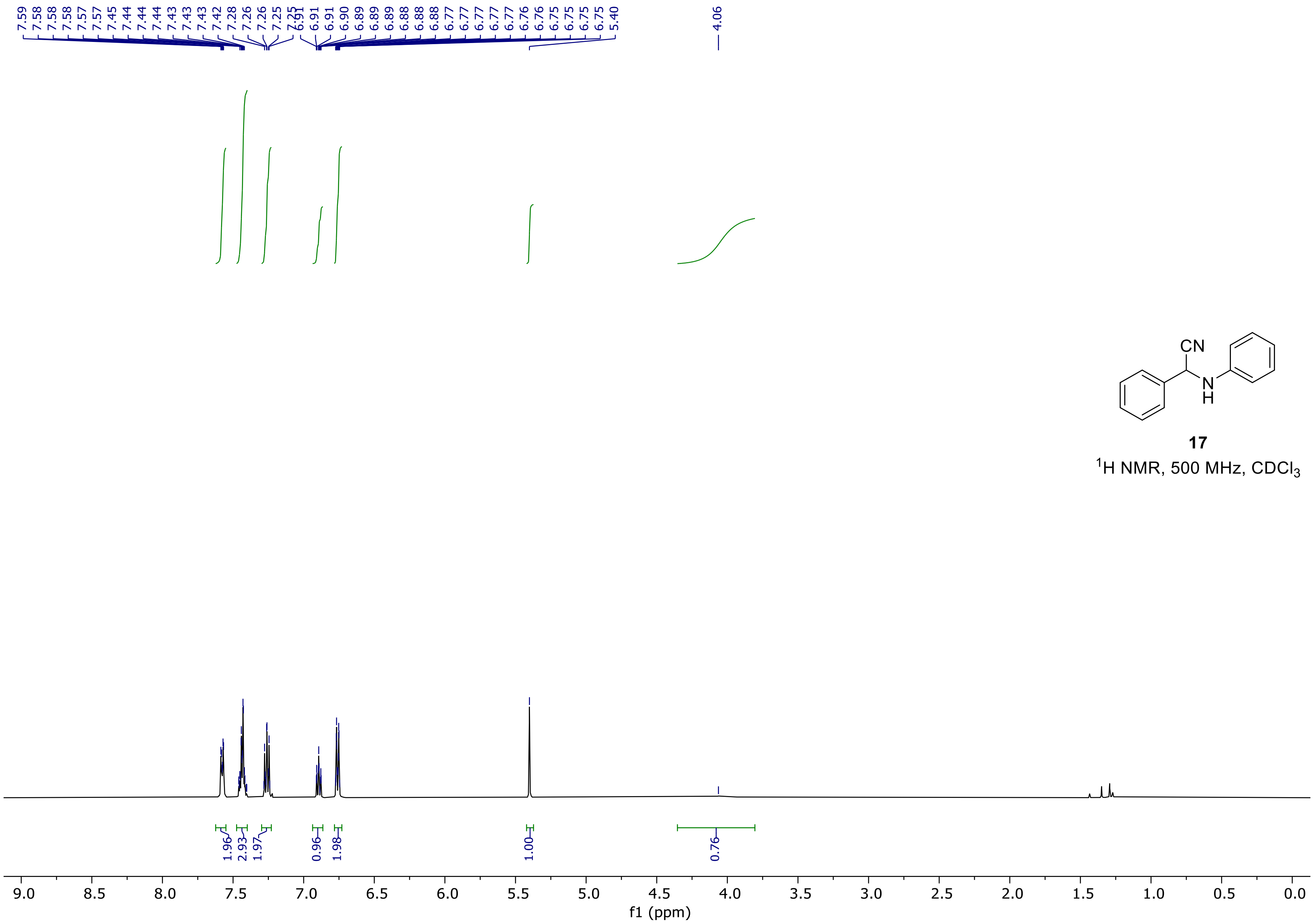

17

^{1}H NMR, 500 MHz, $CDCl_3$

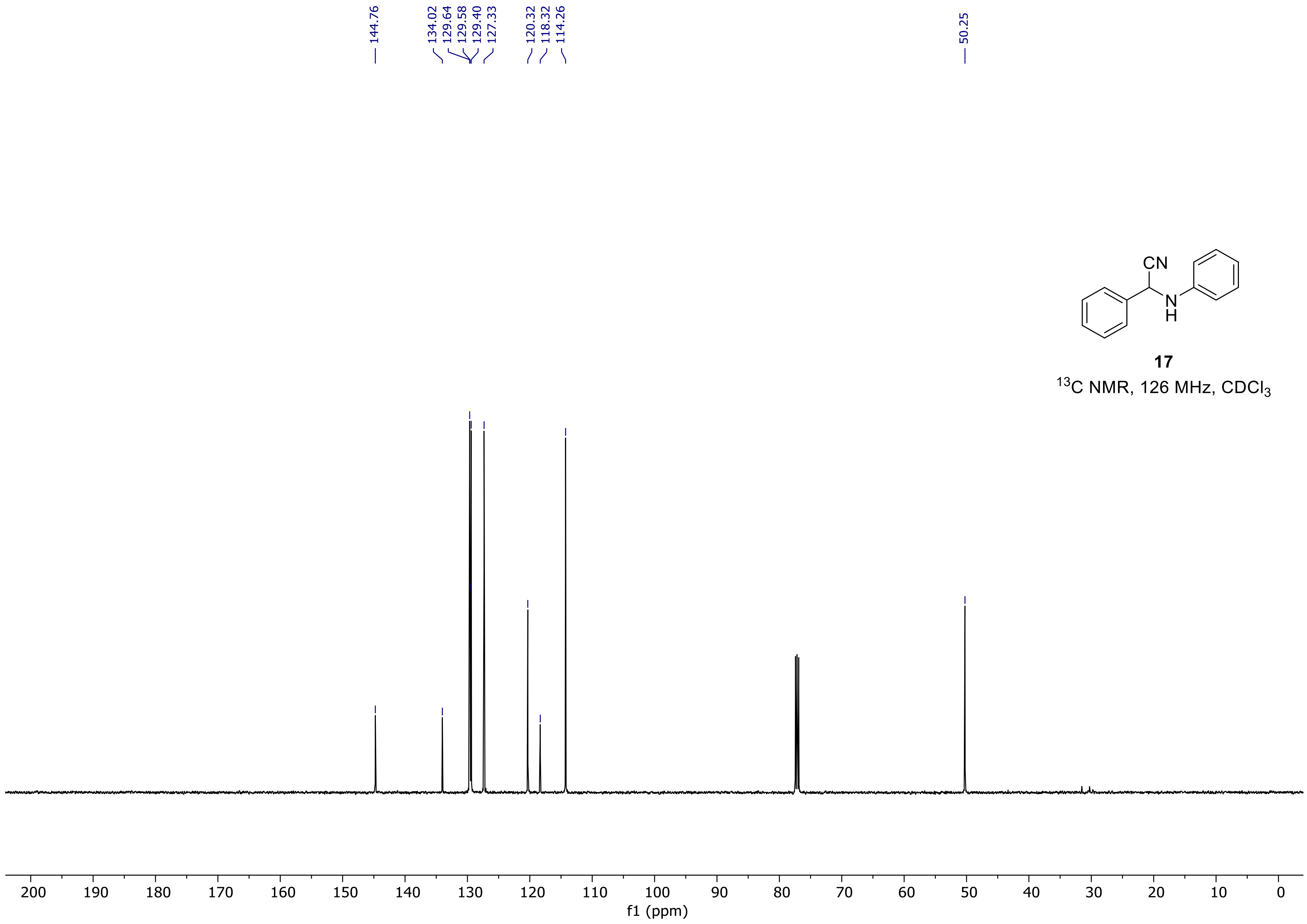

17

^{13}C NMR, 126 MHz, $CDCl_3$
